# Electrocatalytic Semihydrogenation of Alkynes with [Ni(bpy)_3_]^2+^

**DOI:** 10.1021/jacsau.1c00574

**Published:** 2022-02-22

**Authors:** Mi-Young Lee, Christian Kahl, Nicolas Kaeffer, Walter Leitner

**Affiliations:** Max Planck Institute for Chemical Energy Conversion, Stiftstrasse 34-36, 45470 Mülheim an der Ruhr, Germany

**Keywords:** molecular electrocatalysis, alkyne semihydrogenation, nickel catalysis, electrosynthesis, spectroelectrochemistry

## Abstract

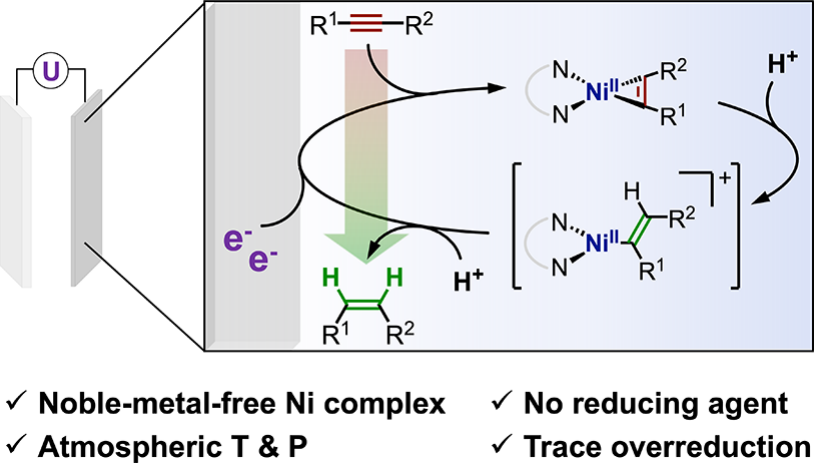

Electrifying the production of base and fine chemicals calls for the development of electrocatalytic methodologies for these transformations. We show here that the semihydrogenation of alkynes, an important transformation in organic synthesis, is electrocatalyzed at room temperature by a simple complex of earth-abundant nickel, [Ni(bpy)_3_]^2+^. The approach operates under mild conditions and is selective toward the semihydrogenated olefins with good to very good *Z* isomer stereoselectivity. (Spectro)electrochemistry supports that the electrocatalytic cycle is initiated in an atypical manner with a nickelacyclopropene complex, which upon further protonation is converted into a putative cationic Ni(II)–vinyl intermediate that produces the olefin after electron–proton uptake. This work establishes a proof of concept for homogeneous electrocatalysis applied to alkyne semihydrogenation, with opportunities to improve the yields and stereoselectivity.

The increasing electrification of the energy sector is expected to significantly impact process technologies in other sectors, including the chemical industry. Electrocatalysis is therein instrumental to facilitate the conversion of chemicals using renewable electricity as the energy income.^1−4^ This approach appears to be particularly valuable in widespread hydrogenation reactions, for which green electrons provide sustainable reducing equivalents to substitute for hydrogen or sacrificial reductants used in conventional routes. The semihydrogenation of alkynes to form alkenes (Figure 1a) is a prominent example of catalytic hydrogenation,^5,6^ used industrially in acetylene removal from ethylene streams^6,7^ but also in fine chemical synthesis.^5,8,9^ The reaction is conventionally performed over heterogeneous catalysts based on rare metals, such as Lindlar Pd,^10^ although efficient catalytic systems made of nanoparticles or molecular complexes of earth-abundant metals (e.g., Co,^11,12^ Cu,^13,14^ and Ni^15−20^) were recently disclosed. The electrochemical counterpart, electrocatalytic alkyne semihydrogenation, has to date been explored only with heterogeneous catalytic systems,^21−32^ often based on noble metals.^28−32^

**Figure 1:**
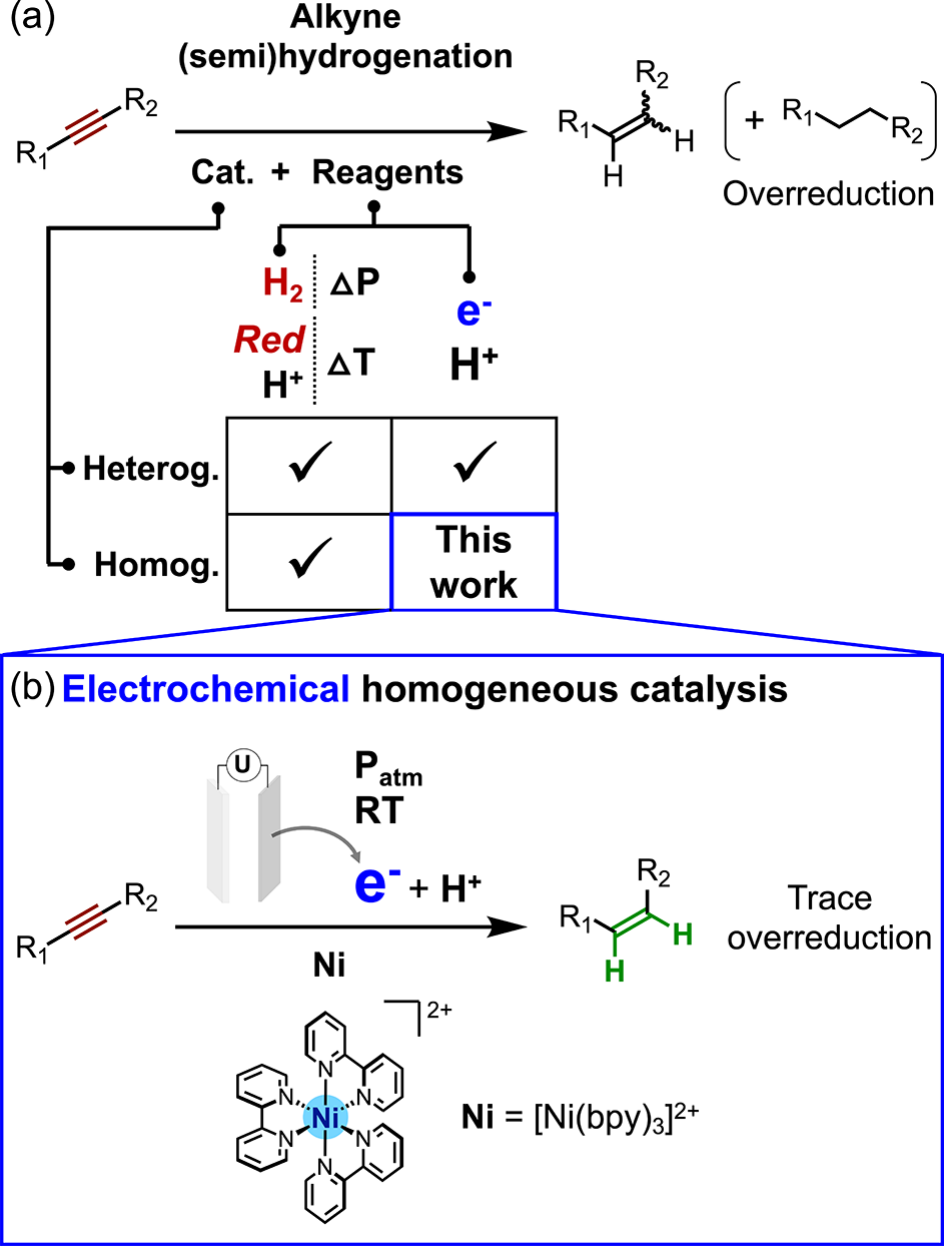
Alkyne semihydrogenation: (a) established approaches and (b) the new approach of electrochemical homogeneous catalysis developed here. (Cat., catalyst; heterog./homog., hetero/homogeneous; *Red*, reducing agent).

Here we report the first example of electrocatalytic alkyne semihydrogenation using a homogeneous molecular catalyst, the readily accessible complex [Ni(bpy)_3_](BF_4_)_2_ (**Ni**(BF_4_)_2_)^33^ (Figure 1b) based on an earth-abundant 3d metal. **Ni** is electrocatalytically active at room temperature and needs only a simple proton source to effectively release the (*Z*)-olefin. In addition, mechanistic investigations conducted by (spectro)electrochemical methods indicate the involvement of an nickelacyclopropene complex as a central intermediate, which is protonated to form a putative cationic Ni(II)–vinyl species.

An initial investigation of the electrochemical behavior of **Ni** by cyclic voltammetry (CV) in DMF (Figures 2a and S1) was in agreement with previous literature reports.^33−36^ A first reduction wave of *E*_p,c_= −1.64 V vs Fc^+/0^ (abbreviated as V_Fc_) (see Figure S1) is attributed to the two-electron reduction of [Ni(bpy)_3_]^2+^ into [Ni(bpy)_3_]^0^, followed by release of a bipyridine ligand to give [Ni(bpy)_2_]^0^ (Figure S41a).^33−35^ The following reversible waves at *E*_1/2_ = (E_p,a_+E_p,c_)/2 = −2.39 and −2.65 V_Fc_ (Figure S1) are assigned to the [Ni(bpy)_2_]^0/–^ couple and the bpy^0/–^ couple of released free bipyridine, respectively.^33,34^

The addition of 4-octyne (**1**) (10 equiv vs **Ni**) produces a positive shift of the [Ni(bpy)_3_]^2+^ reduction wave and the [Ni(bpy)_2_]^0/–^ couple (blue lines in Figure 2a; Figure S2), plausibly showing coordination of the alkyne to reduced **Ni**.^33^ Further addition of benzoic acid (**BA**) (50 equiv vs **Ni**) as a proton source results in an enhanced cathodic peak current for the reduction of [Ni(bpy)_3_]^2+^ (*E*_p,c_ = −1.64 V_Fc_) and the appearance of an irreversible reduction wave from ca. −1.80 V_Fc_ (red lines in Figure 2a), both suggesting electrocatalytic activity. CVs excluding the alkyne under otherwise identical conditions do not show magnification of the first reduction wave (green line in the inset of Figure 2a; Figure S5), discarding electrocatalytic proton reduction at that wave. We thus hypothesized that the larger cathodic currents building upon concomitant alkyne and H^+^ addition testify to electrocatalytic reduction of the alkyne.

**Figure 2:**
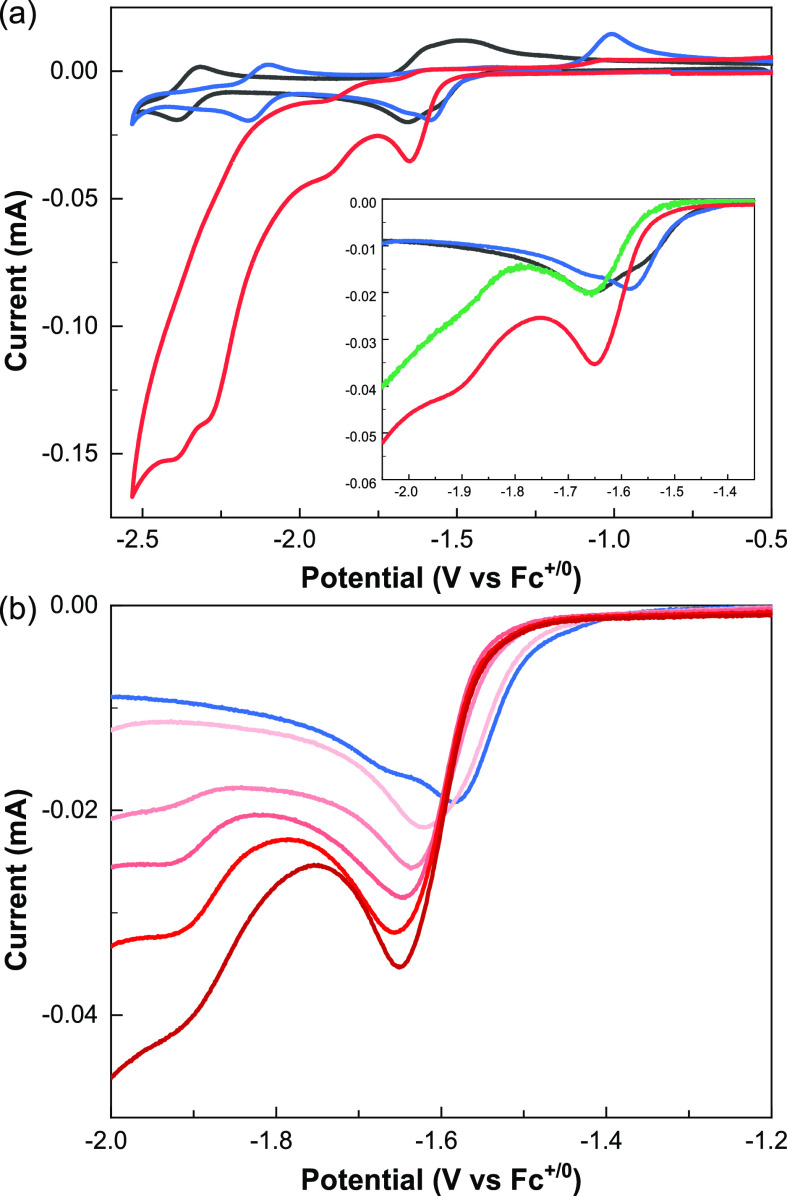
Voltammograms of **Ni** (1 mM) alone (dark gray) or in the presence of **1** only (blue), **BA** only (green, inset), or **1** and **BA** (red): (a) [**1**] = 10 mM, [**BA**] = 50 mM; (b) [**1**] = 100 mM, [**BA**] = 1, 5, 10, 20, 50 mM (from light-red to dark-red). The supporting electrolyte was DMF 0.1 M *n*Bu_4_NPF_6_. The scan rate (ν) was 0.1 V·s^–1^.

The standard potential of the alkyne/alkene (**1**/**1**H_2_) redox couple is ca. *E*°(**1**/**1**H_2_) = −0.82 V_Fc_ in our conditions. Alkyne semihydrogenation is thus thermodynamically feasible in the potential range −1.5 to −2.0 V_Fc_ considered here and even favored compared with the hydrogen evolution reaction (HER) (cf. Supporting Information (SI) section 2 for details).

The actual electrocatalytic activity of **Ni** toward alkyne conversion was then assessed by room-temperature electrolyses, applying potentials at or just cathodic to that for the two-electron reduction of **Ni** (Table S2).^37^ Under our standard conditions ([**Ni**] = 1 mM; [**1**] = 10 mM; [**BA**] = 100 mM; *E*_app_ = −1.93 V_Fc_^37^), we observed that **1** is successfully fully converted within 40 min to the respective (*Z*)-olefin in 69% yield with 53% Faradaic efficiency (F.E.), corresponding to a turnover number (TON) of 6.8 (Table S2). The same conditions exempt of **Ni** or **BA** showed only traces of activity (4% and 2% conversion, respectively). Substituting **Ni**(BF_4_)_2_ with the Ni(II) complex [Ni(MeCN)_6_](BF_4_)_2_ results in only 6% conversion (Figure S17). In addition, nonrinsed postactivity electrodes^38,39^ are barely active in the absence of **Ni**, despite the presence of residual adsorbed Ni (see SI section 3.2.3.6 for details). These points all strongly support that electrocatalysis occurs at well-defined molecular species of the type [Ni(bpy)_*n*_]^*q*^.

Screening for optimized reaction conditions evidenced that increasing alkyne concentration results in lower conversions, yields, F.E., and TONs, whereas the opposite is observed for increasing acid concentration, reaching up to 83% yield, 58% F.E., and a TON of 8.3 at 500 mM [**BA**] (Table S2). Applying larger or smaller overpotentials (*E*_app_ = −2.28 or −1.68 V_Fc_, respectively^37^) still afford full conversion of **1** but lower the F.E. and, for the more anodic conditions, also the yield (46%). Balancing the activity and excess acid equivalents, we thus selected the standard conditions summarized in Table 1 to screen other substrates.

**Table 1:**
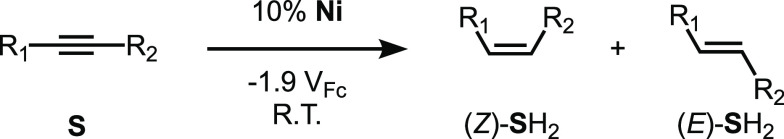

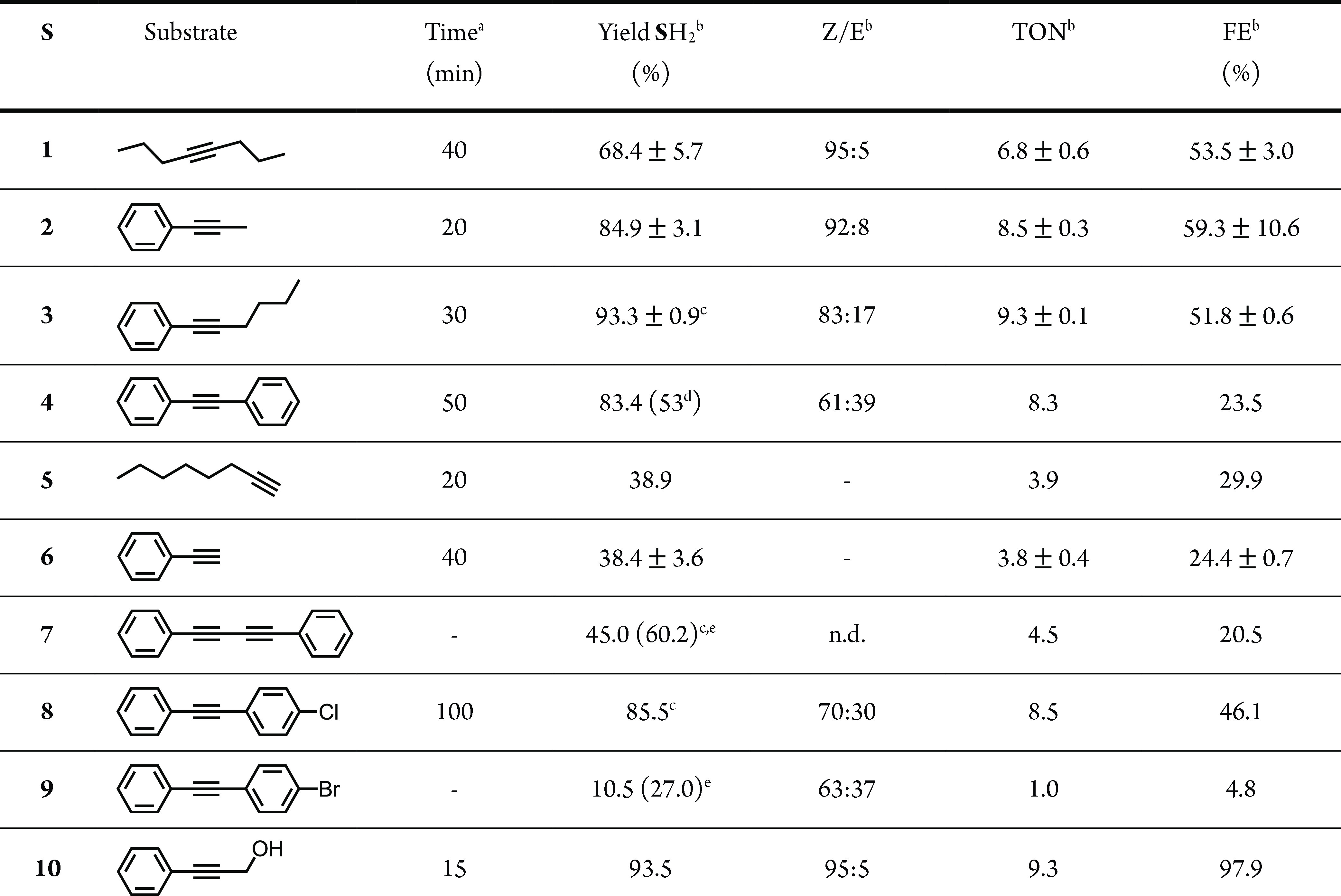
Electrocatalytic Hydrogenation of Alkynes with **Ni** at Room Temperature ([**Ni**] = 1 mM; [**S**] = 10 mM; [BA] = 100 mM; DMF; 0.1 M *n*Bu_4_NPF_6_; *E*_app_ = −1.93 V_Fc_;^37^ 2.5 h)

a Time to full conversion.

b At time to full conversion.

c Trace overhydrogenation (see SI section 3.2.4).

d Isolated yield.

e Not fully converted at 2.5 h; conversion in parentheses.

The electrocatalytic transformation of 10 terminal and internal alkynes **S** (**1** to **10**; Table 1) was investigated under these conditions. Full conversion is reached for simple internal alkynes (**1**–**4**), which produce the corresponding olefins in good to high yields (68–93%). The reaction is highly stereoselective toward *cis*-olefins (viz. 4-octene, β-methylstyrene, 1-phenyl-1-hexene) except in the case of diphenylacetylene, which evolves a 61:39 mixture of (*Z*)- and (*E*)-stilbene^40^ (cf. Figures S21–S28). Terminal alkynes (**5** and **6**) are also fully converted, although in limited olefin yields (39 and 38%, respectively), likely because of secondary reactions to give oligomeric species (vide infra).

The intermediate conversion of a conjugated diyne (**7**) predominantly produces the corresponding enynes. Chloro substitution (**8**) slows the conversion but still affords high olefin yield, whereas bromo substitution (**9**) quenches the reactivity. Good tolerance is observed for the propargylic alcohol of substrate **10**, which evolves the corresponding (*Z*)-olefin in high yield and F.E. Importantly, full alkyne conversion in general proceeds with overhydrogenation below traces, featuring intrinsic semihydrogenation selectivity of the system (see SI section 3.2.3.2). The F.E. values in olefin at full conversion are usually moderate, which result from the occurrence of side reductions, the most plausible being hydrogen evolution over Ni species.

We then investigated possible mechanisms for alkyne semihydrogenation electrocatalyzed by **Ni** through CV and in situ ultraviolet–visible (UV) and infrared (IR) spectroelectrochemistry (SEC). Upon addition of excess **1** (≤5 equiv), the CV wave for two-electron reduction of **Ni** to [Ni(bpy)_2_]^0^ (see above and refs (33−35)) becomes irreversible, shifts to a less negative cathodic peak potential (*E*_p,c_ = −1.60 V_Fc_) and triggers a reoxidation event at *E*_p,a_ = −1.40 V_Fc_ in the backward scan (Figure S2). In conjunction, the [Ni(bpy)_2_]^0/–^ couple at *E*_1/2_ = −2.36 V_Fc_ evolves into a reversible wave at *E*_1/2_ = −2.30 V_Fc_(Figure S2). A stoichiometric amount of **1** (**Ni**:**1** = 1:1) suffices to provoke these changes (Figure S2) and to induce the release of a second bipyridine per **Ni** upon two-electron reduction (see SI section 3.1.2.2 and Figures S3 and S4). We thus conclude that the complex evolved from the doubly reduced **Ni** and alkyne **1** is formulated as [Ni(bpy)**1**]^0^ (**Ni-I**) (Figure 3), consistent with previous literature reports.^33^ This intermediate is best described as a Ni(II) nickelacyclopropene complex.^41,42^ The in situ UV-SEC electrolysis of **Ni** and **1** at an applied potential of −1.93 V_Fc_ (Figure S39) evolves charge-transfer bands (at 438 and 695 nm) in spectral regions reported for the formation of nickelacyclopropene [Ni(bpy)(alkyne)] fragments.^41^

**Figure 3:**
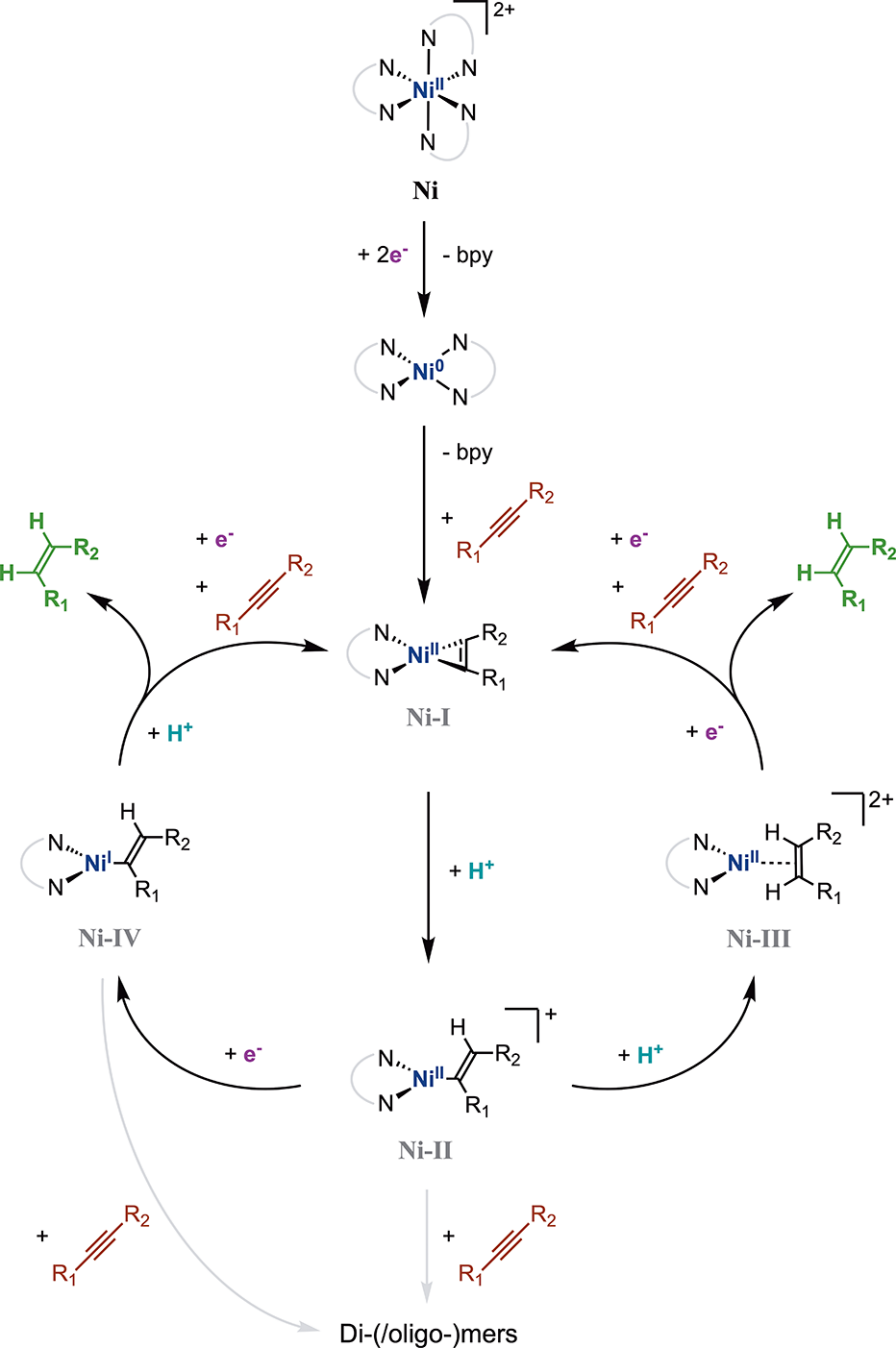
Proposed mechanistic pathways for the electrocatalytic semihydrogenation of alkynes using **Ni**.

Moreover, the corresponding IR-SEC experiment using alkyne **2** (to avoid overlay with the solvent signature) produces a band at 1924 cm^–1^ (Figure S40). We tentatively attribute this band to the C–C stretching of the nickelacycle in [Ni(bpy)**2**] by comparison with the literature (proposed at 1770 cm^–1^ in [Ni(bpy)**4**];^42^ see SI section 3.3 for details). These results further strengthen our assignment of the structure of the electrochemically generated **Ni-I**. The CV wave at *E*_1/2_ = −2.30 V_Fc_ is then attributed to a [Ni(bpy)**1**]^0/–^ couple and the reoxidation wave at *E*_p,a_ = −1.40 V_Fc_ to [Ni(bpy)**1**]^0^ oxidation (Figure S2).

When only **BA** is added to the reaction mixture, the two-electron-reduction wave of **Ni** is only marginally affected with regard to peak potential and current density (Figure S5). We thus discard the formation of a catalytically competent nickel hydride species at the potential of this wave,^43^ from which it follows that the electrocatalytic cycle is initiated via nickelacyclopropene **Ni-I** (Figures 3, S41b, and S42).

At fixed concentrations of **Ni** and alkyne **1**, raising the concentration of **BA** (**Ni**:**1**:**BA** = 1:100:0–50) was found to magnify the first reduction wave (Figures 2b and S6b,c), indicating that protonation of **Ni-I** is related to the buildup of electrocatalytic current. At the potential of this wave, we can rule out a stepwise electron transfer (ET) to **Ni-I** preceding protonation, as the reduction of [Ni(bpy)**1**] requires a more negative potential (*E*_1/2_([Ni(bpy)**1**]^0/–^) = −2.30 V_Fc_; vide supra). Rather, a plausible transformation involves opening of the **Ni-I** nickelacycle via proton transfer (PT) to form a cationic Ni(II)–vinyl complex **Ni-II** (Figures 3 and S42).^44^**Ni-II** can then bifurcate between a second PT (protonation-first pathway) to give the dicationic Ni(II) olefin complex [Ni(bpy)(alkene)]^2+^ (**Ni-III**) (Figure 3) or an ET step (reduction-first pathway) evolving the neutral Ni(I)–vinyl complex [Ni(bpy)(vinyl)]^0^ (**Ni-IV**) (Figure 3).

The occurrence of a protonation-first pathway is supported by the reported observation that twofold protonation of Ni(II) nickelacyclopropene [Ni(bpy)**4**] results in release of the respective olefin **4**H_2_.^42,45^ Furthermore, the absence of electrocatalytic activity for olefin hydrogenation (Figure S14) points to rapid and irreversible olefin release from the Ni center (presumably via exchange with solvent or bpy). Once the olefin is displaced, the complex can then re-engage in reduction and alkyne activation to form **Ni-I**.

We note that metallacyclopropene pathways in homogeneous alkyne semihydrogenation, such as the one proposed here, are uncommon but have precedents (with Y–Ni,^19^ Ti,^46^ Zn,^47^ and Nb^48^ complexes).

Finally, we observed that the electrocatalytic waves at −1.65 and from −1.80 V_Fc_ are partially inhibited in excess alkyne, e.g., **2** (Figures S8 and S9), evidencing a competition between binding of additional alkyne(s) and protonation in the catalytic cycle. In the absence of a proton source, the CV signature of **Ni-I** observed from a **Ni**/**2** stoichiometric mixture remains unmodified in the presence of excess **2** (Figure S7a). We thus deduce that additional alkyne coordination does not occur at **Ni-I** but at a later stage of the catalytic cycle. In particular, increasing [**BA**] in the presence of large excess of **2** (100 equiv of **BA**; Figure S8b,c) gradually restores the inhibited electrocatalytic waves. These results indicate that excess alkyne(s) coordination occurs at stages preceding protonation, likely Ni–vinyl species **Ni-II** and **Ni-IV** (Figure S41c). Additional alkyne binding to Ni–vinyl intermediates is also supported by the GC-MS analysis of the electrolytic mixture of phenylacetylene (**6**), which shows the formation of unsaturated dimeric coupling products (Figure S31). Such di/oligomerization processes are known with similar Ni species^49,50^ and here constitute side reactions competing with the desired hydrogenation to olefins.

In summary, we have shown here that the semihydrogenation of internal and terminal alkynes is electrocatalyzed by [Ni(bpy)_3_]^2+^ at room temperature using a simple organic proton donor. The system generally discards overhydrogenation and predominantly produces the semihydrogenated olefins with good to very good *Z* stereoselectivity. (Spectro)electrochemical experiments support that the electrocatalytic cycle is entered via a nickelacyclopropene intermediate, which is a rather atypical initiation route in homogeneous alkyne hydrogenation. This intermediate opens upon protonation to give a putative cationic Ni(II)–vinyl intermediate, from which either a second protonation or a reduction–protonation sequence evolves the olefin. Additional alkyne insertion competes with protonation steps at on-cycle intermediates and leads to oligomeric byproducts detrimental to the olefin yield. This proof of concept for homogeneous electrocatalytic alkyne semihydrogenation offers the potential to operate under mild conditions using readily accessible (Ni) complexes without precautions for the handling of sensitive catalysts or the use of hydrogen. Studies are ongoing to suppress undesired oligomerizations and to control the stereoselectivity by structural optimization of the molecular catalyst.

## Supporting Information Available

Detailed experimental procedures, thermodynamic calculations of standard potentials, additional electrochemical data, NMR spectra, GC and GC-MS data, UV and IR-SEC data, and additional mechanistic discussion (PDF)
