# Supplementary material for: Electrocatalytic Semihydrogenation of Alkynes with [Ni(bpy)3]2+
Source: JACS Au. 2022 Feb 22;2(3):573–8. doi: 10.1021/jacsau.1c00574 (PMC8970006; doi:10.1021/jacsau.1c00574)
Supplement: Supplementary file 1 — au1c00574_si_001.pdf [file au1c00574_si_001.pdf]

Supporting Information

Electrocatalytic Semihydrogenation of Alkynes with $[Ni(bpy)_3]^{2+}$

Mi-Young Lee, Christian Kahl, Nicolas Kaeffer,* Walter Leitner

Max Planck Institute for Chemical Energy Conversion, Stiftstrasse 34-36, 45470 Mülheim an der Ruhr, Germany.

*Corresponding author:

Nicolas Kaeffer (nicolas.kaeffer@cec.mpg.de)

Contents	Pages
**1. Experimental procedures**	S3
1.1 General considerations	S3
1.2 Synthesis of [Ni(bpy)$_3$](BF$_4$)$_2$	S3
1.3 Electrochemical experiments	S4
1.4 Analytical methods	S6
**2. Thermodynamic calculation of standard potential**	S8
**3. Supplemental results**	S10
3.1 Electrochemical analysis	S10
*3.1.1 Electrochemical behavior of **Ni***	S10
*3.1.2 Electrochemical behavior of **Ni** with **1***	S11
*3.1.2.1 **Ni** with **1***	S11
*3.1.2.2 Calculation of bipyridine released from **Ni***	S12
*3.1.2.3 **Ni** with **BA***	S14
*3.1.2.4 **Ni** with **1** and **BA***	S15
*3.1.3 Electrochemical behavior of **Ni** with **2***	S16
*3.1.3.1 **Ni** with **2***	S16
*3.1.3.2 **Ni** with **2** and **BA***	S17
*3.1.3.3 **Ni** with **2** and **TFA***	S19
3.2 Electrochemical hydrogenation of alkynes	S20
3.2.1 Screening of conditions	S20
3.2.2 iR-corrected potentials applied in electrolysis	S23
3.2.3 Electrolysis control experiments	S24
3.2.3.1 Extended electrolysis	S24
3.2.3.2 With olefin substrate	S25
3.2.3.3 Without and with applied potential	S26
3.2.3.4 Under H$_2$	S27
3.2.3.5 Using [Ni(MeCN)$_6$](BF$_4$)$_2$	S28
3.2.3.6 With post-activity electrodes	S29
3.2.4 Substrate scope	S33
3.2.5 Isolated yield for stilbene	S52
3.3 SEC analysis	S53
3.4 Additional mechanistic discussion	S55
**4. References**	S57

1. Experimental procedures

1.1 General considerations

Synthetic manipulations were performed under inert conditions of argon atmosphere either in an MBRAUN UNILAB Plus glove box or using standard Schlenk techniques, in oven-dried glassware. Organic solvents (analytical grade; Carl Roth) other than *N,N*-Dimethylformamide (DMF) were dried and degassed by passage over an MBRAUN SPS-7 solvent purification system, handled under argon atmosphere and stored over molecular sieves. Deionized water was obtained using a Merck Millipore system. DMF (99.8%, Extra Dry over Molecular Sieve) was obtained from ACROS Organics. 2,2-Bipyridine (≥ 99%) and cis-4-octene (97%) were purchased from Alfa Aesar (Germany). Tetrabutylammonium hexafluorophosphate ($n\mathrm{Bu_4NPF_6}$) (for electrochemical analysis, ≥ 99.0%), 4-octyne (99%), 1-octyne (97%), 1-phenyl-1-propyne (99%), diphenylacetylene (98%), phenylacetylene (98%), 3-phenyl-2-propyn-1-ol (96%), 1-chloro-4-(phenylethynyl)benzene (98%), 1-octene (98%), trans-β-methylstyrene (99%), propylbenzene (> 98%), cis-stilbene (96%), styrene (≥ 99%), benzoic acid (≥ 99.5%), chloroform-d (99.8 atom % D) and silica gel (pore size 60 Å, 40-63 µm particle size) were purchased from Merck (Darmstadt, Germany). 1-phenyl-1-hexyne (> 98%), 1-bromo-4-(phenylethynyl)benzene (98%), (E)-cinnamyl alcohol (97%), n-octane (97%), dibenzyl (99%) were obtained from TCI Deutschland GmbH (Germany). 1,4-diphenyl-butadiyne (99%) was purchased from Thermo Scientific (Germany). Dichloromethane-d2 (99.8 atom % D) was purchased from abcr GmbH (Germany). H_2 (99.999 mol%, ALPHAGAZ™ 1 Hydrogen) was obtained from Air Liquide (Germany). The chemicals in the solid state were dried under vacuum at 110°C over 6 hours, and the chemicals in the liquid state were filtered over a pad of silica gel before use. $\mathrm{[Ni(MeCN)_6](BF_4)_2}$, was prepared following literature procedure.[1]

1.2 Synthesis of $\mathrm{[Ni(bpy)_3](BF_4)_2}$

$\mathrm{[Ni(MeCN)_6](BF_4)_2}$ (199.6 mg, 0.417 mmol, 1 equiv.) and 2,2-bipyridine (196.5 mg, 1.258 mmol, 3 equiv.) were introduced in a Schlenk tube in the glovebox. Taken out of the glovebox, the Schlenk tube was connected to a Schlenk line and 10 mL of dichloromethane were added. The resulting suspension was stirred at room temperature overnight. The solvent was evaporated under vacuum and the obtained solid washed 3 times with diethyl ether and dried under vacuum at least 3 hours to afford 186.4 mg of $\mathrm{[Ni(bpy)_3](BF_4)_2}$ (**Ni**$\mathrm{(BF_4)_2}$) as a pink solid (yield = 63.7%).

Elemental analysis found (calculated for $\mathrm{C_{30}H_{24}N_6B_2F_8Ni}$): C: 49.7±1.2% (51.4%), H: 3.7±0.4% (3.5%), N: 11.7±0.4% (12.0%). **HRMS** found (calculated for $\mathrm{C_{30}H_{24}N_6Ni^{2+}}$): m/z = 263.07031 (263.070796).

1.3 Electrochemical experiments

All electrochemical experiments were performed outside of the glovebox, in DMF 0.1 M *n*Bu_4NPF_6 electrolyte solution at room temperature with a **Ni** concentration (when present) of [**Ni**] = 1 mM. A $AgNO_3$/Ag electrode, made of a silver wire (0.5 mm diameter, 99.99%, Goodfellow Cambridge Ltd, UK) in contact with a 10 mM $AgNO_3$ solution in DMF 0.1 M *n*Bu_4NPF_6 bridged to the electrochemical cell via a Vycor frit (092-VYC4, Biologic Science Instruments) was used as reference electrode unless otherwise specified. The electrolyte and analyte mixtures were introduced in the electrochemical cell under Ar flow.

Cyclic voltammetry. Cyclic voltammograms (CVs) were recorded with a VSP-300 (Biologic Science Instruments, France) potentiostat equipped with an analogic ramp generator module. A glassy carbon disk (3 mm diameter, ALS Co., Ltd, Japan) and a coiled platinum wire (0.5 mm diameter, ALS Co., Ltd, Japan) were used as working and counter electrodes, respectively. The one-compartment electrochemical glass cells were filled with 5 mL of the electrolyte solution, and purged with Ar at least for 10 min. The working electrode was polished over a polishing pad using a polishing alumina (0.05 μm, ALS Co., Ltd) suspension, followed by rinsing with deionized water and ethanol. Reference and counter electrodes were rinsed with ethanol. The electrodes were dried under a stream of argon prior to insertion in the cell. After each CV measurements, the working electrode was taken from the cell and freshly polished. Unless otherwise noted, the CVs were recorded at a scan rate (v) of 0.1 $V \cdot s^{-1}$. Ohmic drop compensation (85%) was applied. Reported potentials in CVs are referenced to the ferrocenium/ferrocene ($Fc^{+/0}$) couple (abbreviated to V_{Fc}) by adding ferrocene at the end of the measurements.

Electrolysis. Electrolyses were performed using a SP-300 (Biologic Science Instruments, France) potentiostat equipped with a high current/high voltage (1A/48V) module. Electrolyses were performed in a customized H-type glass cell, having anode and cathode chambers separated by the glass frit (P3 pore size). A carbon foam (0.6 cm × 0.6 cm × 2.4 cm; VC003825, Goodfellow Cambridge Ltd, UK) and platinum mesh (20 x 20 mm, 0.1 mm thickness, Goodfellow Cambridge Ltd, UK) and the $AgNO_3$/Ag electrode described above were used as working, counter and reference electrodes, respectively. First, the appropriate volume of electrolyte was introduced in each chamber of the electrochemical cell and sparged with Ar for 5 min. In a typical experiment, 5 μmol (3.5 mg) of **Ni** (final concentration 1 mM) and 50 μL of a 1 M mesitylene (internal standard) solution in DMF 0.1 M *n*Bu_4NPF_6 (final concentration of mesitylene 10 mM) were then introduced in the cathodic chamber, followed by the additions of electrolyte solutions 1 M in the alkyne under consideration and 1 M in benzoic acid. The anodic chamber was filled with the DMF 0.1 M *n*Bu_4NPF_6 electrolyte solution. The final volume of solution in each chamber was set to 5 mL.

Right before electrolyses, the ohmic drop in the cell was determined and linear sweep voltammetry (LSV) at 0.1 $V \cdot s^{-1}$ from 0 V to -2.5 V vs. $AgNO_3/Ag$ was recorded. Then, electrolyses were performed under potentiostatic conditions without ohmic drop compensation. The cathodic electrolyte was continuously purged with Ar (1 $mL \cdot min^{-1}$) during the run of electrolysis. Aliquots of ca 200 μL from the cathode chamber were collected during the electrolysis at times based on the charge passed (at 0 C and roughly 1, 2.5, 5, 10, 15, 20, and 25 C) and analyzed by gas chromatography (GC). In addition, the cathodic chamber was sampled (150 μL aliquots) prior to and at the end of the electrolysis, the aliquots mixed with 250 μL deuterated chloroform and analyzed by ^1H nuclear magnetic resonance (NMR). Potentials reported for electrolyses are referenced to $Fc^{+/0}$ by external measurement in an independent cell of this couple versus the $AgNO_3/Ag$ electrode used for electrolyses (found at $E_{1/2}(Fc^{+/0})$ = 0.03 V vs $AgNO_3/Ag$).

For the electrolysis under H_2 atmosphere, the cathodic electrolyte containing the alkyne and **Ni** was sparged with a flow of Ar for 20 min, then with a flow of H_2 for 30 min prior to the experiment and constantly during electrolysis.

For experiments assessing post-activity electrodes, at the end of a standard electrolysis, the working electrode (carbon foam) was quickly disconnected, taken out of the solution and was not rinsed. The final electrolyte solution was removed from both chambers of the electrolysis cell. The cell, which was not rinsed, was then filled with the same electrolyte containing the alkyne and acid but exempt of **Ni**. The nonrinsed, previously used working electrode was plunged in the fresh electrolyte and a new electrolysis performed.

Isolation procedure. The electrolysis was performed as described above but with following alteration: the concentrations in **Ni**, alkyne (**4**) and acid (**BA**) were scaled-up by a factor 10: [**Ni**] = 10 mM (50 μmol); [**4**] = 100 mM (500 μmol; 89.1 mg); [**BA**] = 1 M (1 mmol). No internal standard was added in the cell. The electrolysis was performed for 100 min, at which point the current and charge are plateauing.

At the end of the electrolysis, the cathodic electrolyte (5 mL) was mixed with 5 ml deionized water. The mixture warms following water addition and was allowed to cool down to room temperature. The DMF/water phase was extracted 3 times with 10 mL pentane. The combined pentane phases were washed with 10 mL distilled water and directly filtered over a short silica pad (ca 9 g). Full elution of the product with additional volumes of pentane was followed by thin layer chromatography. The desired fractions were combined and the solvent removed on rotary evaporator. The obtained oil was taken in ca 5 mL of diethyl ether and the solvent removed on rotary evaporator to afford the final product as an oily colorless solid (48.2 mg; 53% yield).

Spectroelectrochemical experiments (SEC). The ultraviolet-visible (UV) and infrared (IR) spectra were recorded with an Agilent spectrophotometer (Cary 8454) and a Shimadzu spectrometer (IRAffinity-1S), respectively. The potentiostat

(VSP-300, Biologic Science Instruments, France) was connected to the UV or IR spectroelectrochemical cell. Spectroscopic measurements were recorded during chronoamperometry at –1.9 V (vs reference electrode).

UV-SEC experiments were performed in a thin-layer quartz glass cell (1 mm optical path length, 013511 spectroelectrochemical cell kit, ALS Co., Ltd, Japan) using a gold mesh in the optical path, a platinum wire, and the $AgNO_3$/Ag electrode described above as working, counter, and reference electrodes, respectively. The cell was filled with 1 mL of the electrolytic solution under investigation. The spectra were recorded every 10 s for 10 min.

IR-SEC experiments were conducted in an optically transparent thin-layer electrochemical (OTTLE; Department of Chemistry, University of Reading) cell fitted with NaCl windows, equipped with a gold mesh working electrode in the optical path, a Ag wire pseudo-reference electrode, and a platinum mesh counter electrode.[2] The OTTLE cell was filled with 0.3 mL of the solution under investigation. The spectra were recorded every 30 s for 5 min.

1.4 Analytical methods

Samples were analyzed by gas chromatography using gas chromatographs equipped with a flame ionization detector (GC-FID; Nexis GC-2030, Shimadzu, Japan) with elution over a Rtx-1 column (30 m × 0.25 mm with 0.5 μm film thickness, Restek Corp., USA) with He as a carrier gas and a gas chromatograph equipped with a mass spectrometer (GC-MS; QP2020 NX, Shimadzu, Japan) with elution over a Rtx-1 column (30 m × 0.25 mm with 0.5 μm film thickness, Restek Corp., USA) with He as a carrier gas. ^{1}H NMR was recorded on a Bruker Avance Neo 400 (^{1}H: 400 MHz) spectrometer at room temperature. The chemical shifts (δ) are given versus tetramethylsilane (TMS), using the solvent residual signals (DMF δ = 8.03 ppm, $CDCl_3$ δ = 7.26 ppm or CD_2Cl_2 δ = 5.32 ppm) to reference.

Integrals of the GC-FID and NMR peaks of the substrates and products were normalized over the one of the internal standard (mesitylene) for quantification. The quantification of carbon balance, alkyne conversion, alkene yield, faradaic efficiency (F.E) toward alkenes and turnover numbers (TONs) were calculated using the following equations:

$$\text{Carbon balance (\%)} = \frac{C_t(\mathbf{S}) + C_t(\mathbf{SH_2})}{C_i(\mathbf{S}) + C_i(\mathbf{SH_2})} \times 100 \qquad \textbf{(1-1)}$$

$$\text{Conversion (\%)} = \frac{C_i(\mathbf{S}) - C_t(\mathbf{S})}{C_i(\mathbf{S})} \times 100 \qquad \textbf{(1-2)}$$

$$\text{Selectivity} = \frac{C_t(\mathbf{SH_2})}{C_i(\mathbf{S}) - C_t(\mathbf{S})} \qquad \textbf{(1-3)}$$

$$\text{Yield (\%)} = \text{conversion} \times \text{selectivity} \qquad \textbf{(1-4)}$$

$$\text{TON} = \frac{n_t(\mathbf{SH_2})}{n_i(\mathbf{Ni})} \qquad \textbf{(1-5)}$$

$$\text{FE}\ (\%) = \frac{2 \times n_t(\mathbf{S}\text{H}_2) \times \text{F}}{Q_t} \times 100 \qquad \textbf{(1-6)}$$

Where $C_i(\mathbf{S})$, $C_t(\mathbf{S})$, $C_i(\mathbf{S}H_2)$ and $C_t(\mathbf{S}H_2)$ are concentrations in alkyne **S** or alkene **S**H_2 at the beginning of reaction (C_i) and at the given time (C_t), $n_t(\mathbf{S}H_2)$ is the amount of alkene at a given time, $n_i(\mathbf{Ni})$ is the amount of **Ni** at the beginning of the reaction, Q_t is the charge passed through the system at a given time and F is the Faraday constant (96485 $C{\cdot}mol^{-1}$). Carbon balance, alkyne conversion, yields in alkenes were quantified from GC-FID measurements, unless otherwise noted. The reported *Z*/*E* ratios of products are evaluated based on the integration of the signals of the olefinic protons in ^1H NMR, unless otherwise noted. In the case of 4-octenes, the *Z*/*E* ratio was calculated from the GC-FID chromatograms using two standard isomers. The presence of detectable amounts of alkane products was assessed using GC-FID when reference alkane compounds are available. When reference alkane compounds are not available, GC-MS was used to provide a qualitative evaluation of the presence of alkane.

Analysis of the post-activity electrodes was performed following methods established in literature.[3, 4] The working electrode was namely disconnected right after electrolysis and taken out of the solution.

For electron microscopy, the working electrode was dried under a N_2 stream overnight and the dried sample was mounted on an aluminum stub with gold holder and introduced in the electron microscope. Scanning electron microscopy (SEM) and energy dispersive X-ray spectroscopy (EDX) were recorded on S-5500 (Hitachi, Japan) microscope at an acceleration voltage of 30 kV.

For metal trace titration, the working electrode was digested in a 69% HNO_3 solution for 15 min at 200°C using a microwave oven (MARS6, CEM, USA) and the resulting solution submitted to analysis by inductively coupled plasma mass spectrometry (ICP-MS; ICPMS-2030, Shimadzu, Japan).

2. Thermodynamic calculation of standard potential

The interconversion of an alkyne **S** with the corresponding alkene **S**H2 in a solvent (*s*) with the presence of an acid (HA) is described by:

$$\mathbf{S}\ (s) + 2\ \mathrm{HA}\ (s) + 2e^{-} \rightleftharpoons \mathbf{S}H_2\ (s) + 2\ \mathrm{A}^{-}\ (s)$$

with the corresponding standard potential $E^0_{\mathbf{S}/\mathbf{S}H_2,HA,s}$.

Two consecutive reactions (**2-1** and **2-2**) are considered.

$$2\ \mathrm{HA}\ (s) + 2\ e^{-} \rightleftharpoons H_2\ (s) + 2\ \mathrm{A}^{-}\ (s) \qquad E^0_{HA/H_2,s} \qquad \textbf{(2-1)}$$

$$\mathbf{S}\ (s) + H_2\ (s) \rightleftharpoons \mathbf{S}H_2\ (s) \qquad \Delta G^0_{Hydrogenation(\mathbf{S}),s} \qquad \textbf{(2-2)}$$

$E^0_{\mathbf{S}/\mathbf{S}H_2,\ HA,\ s}$ can thus be calculated following equation **2-3**:

$$E^0_{\mathbf{S}/\mathbf{S}H_2,HA,s} = E^0_{HA/H_2,s} - \frac{\Delta G^0_{Hydrogenation(\mathbf{S}),s}}{2F} \qquad \textbf{(2-3)}$$

The standard potential for HA/H2 interconversion in solvent *s* with the acid HA ($E^0_{HA/H_2,s}$ in equation 1) is given by equation **2-4**:[5]

$$E^0_{HA/H_2,s} = E^0_{H^+/H_2,s} - \ln(10)\frac{RT}{F}pK_{a,HA,s} \qquad \textbf{(2-4)}$$

With $E^0_{H^+/H_2,s}$ the standard potential for H^+/H_2 interconversion in solvent *s* and $pK_{a,HA,s}$ the pK_a of the HA/A^- couple in solvent *s*.

Working at room temperature, we take T = 298.15 K.

In the case of benzoic acid (**BA**) as a proton source in DMF, the parameters are as follow:

$E^0_{H^+/H_2,DMF} = -0.62$ V vs $Fc^{+/0}$ (ref.[5])

$pK_{a,\mathbf{BA},DMF} = 12.2$ (ref.[5])

Thus:

$E^0_{\mathbf{BA}/H_2,DMF} = -1.34$ V vs $Fc^{+/0}$

In first approximation here, we do not account for the homoconjugation of the acid with the corresponding base, although the phenomenon is known and quantified for **BA** in DMF.[5]

The Gibbs energy for the semihydrogenation of **S** in the solvent *s* ($\Delta G^0_{Hydrogenation(\mathbf{S}),\ s}$ in equation **2-2**) can be calculated from the Gibbs energies of formation (ΔG^0_f) in gas phase and the Gibbs energies of solvation in solvent *s* ($\Delta G^0_{solv,s}$) of the substrates and product (eq. **2-5**).

$$\Delta G^0_{\text{Hydrogenation}(\mathbf{S}),s} = (\Delta G^0_{\text{f}(\mathbf{S}\text{H}_2)} - \Delta G^0_{\text{f}(\mathbf{S})} - \Delta G^0_{\text{f}(\text{H}_2)}) + (\Delta G^0_{\text{solv}(\mathbf{S}\text{H}_2),s} - \Delta G^0_{\text{solv}(\mathbf{S}),s} - \Delta G^0_{\text{solv}(\text{H}_2),s}) \quad \textbf{(2-5)}$$

In the case of the hydrogenation of 4-octyne **1** to 4-octene **1**H_2 in DMF, the parameters are as follow:

$\Delta G^0_{\text{f}}(H_2) = -39.0$ kJ·mol^{-1} (ref.[6])

$\Delta G^0_{\text{f}}(\mathbf{1}) = 219.3$ kJ·mol^{-1} (calculated from values in ref.[7])

$\Delta G^0_{\text{f}}(\mathbf{1}H_2) = 96.7$ kJ·mol^{-1} (calculated from values in ref.[7])

$\Delta G^0_{\text{solv}(\text{H}_2),\text{DMF}} = 21.9$ kJ·mol^{-1} (from ref.[8])

$\Delta G^0_{\text{solv}(\mathbf{1}),\text{DMF}} = 15.7$ kJ·mol^{-1} (estimated from the gas-solute partition coefficient of log $K_{\text{solv}} = 3.65$ reported for 1-octyne in ref.[9])

$\Delta G^0_{\text{solv}(\mathbf{1}\text{H}_2),\text{DMF}} = 20.2$ kJ·mol^{-1} (estimated from the gas-solute partition coefficient of log $K_{\text{solv}} = 2.85$ reported for 1-octene in ref.[9])

Thus:

$\Delta G^0_{\text{Hydrogenation}(\mathbf{1}),\text{DMF}} = -83.6 - 17.4 = -101.0$ kJ·mol^{-1}

$E^0_{\mathbf{1}/\mathbf{1}\text{H}_2,\mathbf{BA},\text{DMF}} = -0.82$ V vs $Fc^{+/0}$.

3. Supplemental results

3.1 Electrochemical analysis

3.1.1 Electrochemical behavior of ***Ni***

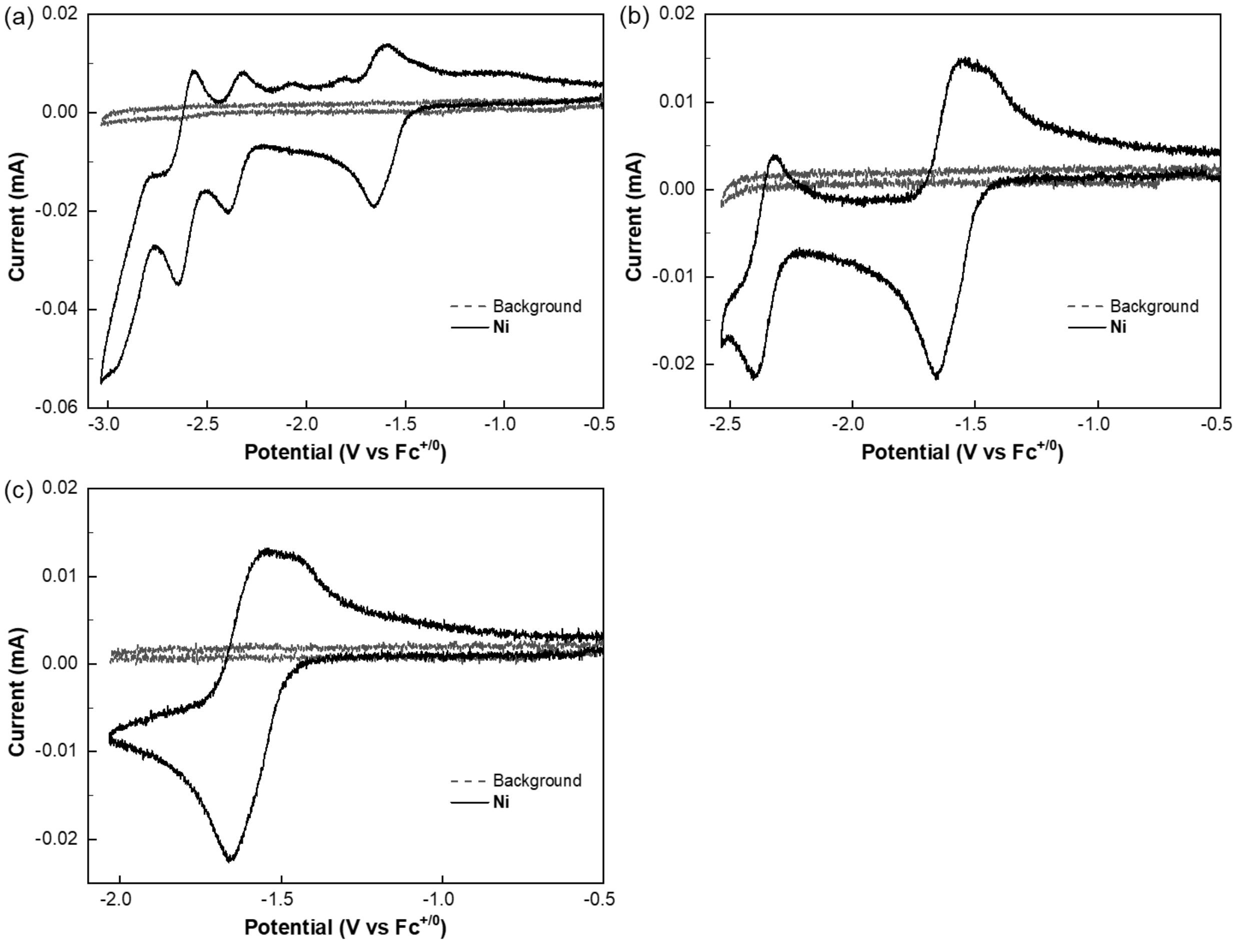

Fig. S1 CVs of **Ni**.

*3.1.2 Electrochemical behavior of **Ni** with **1***

*3.1.2.1 **Ni** with **1***

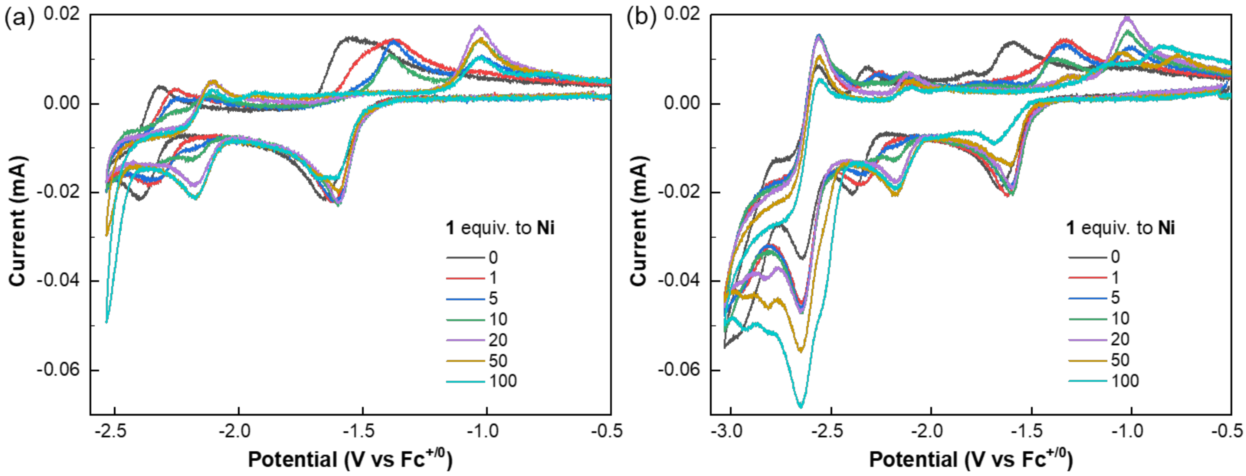

Fig. S2 CVs of **Ni** upon addition of **1** under potentials ranging (a) from –2.5 to –0.5 V_{Fc} and (b) from –3.0 to –0.5 V_{Fc}.

*3.1.2.2 Calculation of bipyridine released from **Ni***

The peak current at a reversible CV wave of a freely diffusing species is given by equation **3-1**:[10]

$$i_{p,c} = 0.446FSC^0\sqrt{D}\sqrt{\frac{F\nu}{RT}} \quad \textbf{(3-1)}$$

where $i_{p,c}$ is peak current, F is Faraday constant (96485.3 C·mol^{-1}), S is the surface area of working electrode, C^0 is concentration of the analyte in bulk, D is diffusion coefficient of the analyte, ν is scan rate, R is universal gas constant (8.314 J·K^{-1}·mol^{-1}), and T is absolute temperature.

From the cathodic peak current at the bpy$^{0/-}$ couple ($E_{1/2}$ = –2.60 V_{Fc}) observed in CVs of bipyridine at different concentrations (Fig. S3), we estimated a diffusion coefficient of bipyridine under our conditions at:

D(bpy) = 6.08 10^{-5} cm^2·s^{-1}.

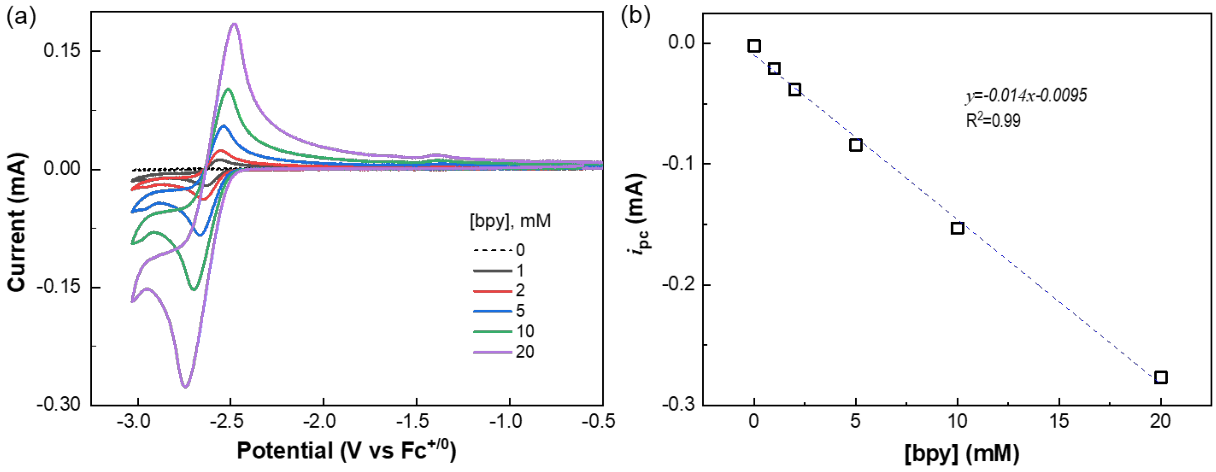

Fig. S3 (a) CV of bipyridine (**bpy**) at different concentrations and (b) the cathodic peak current ($i_{p,c}$) for bpy$^{0/-}$ couple as a function of concentration of bpy (data from (a)).

Using that value, the concentration of bipyridine released from **Ni** upon addition of **1** could be recovered from the cathodic peak current at –2.65 V_{Fc} (Fig. S2b). The values are reported in Table S1 and Fig. S4.

Table S1. Concentration of released bipyridine from **Ni** upon addition of **1**.

[**1**] (mM)	$i_{p.c}$ (mA)	Released [**bpy**] from Ni (mM)
0	-0.019	1.3
1	-0.031	2.1
5	-0.030	2.0
10	-0.031	2.1
20	-0.033	2.2
50	-0.043	2.9
100	-0.055	3.7

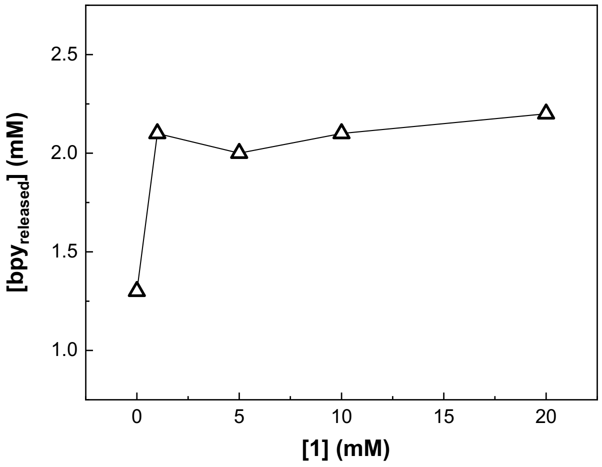

Fig. S4 Concentration of released bpy from **Ni** upon addition of **1**.

3.1.2.3 ***Ni*** *with* ***BA***

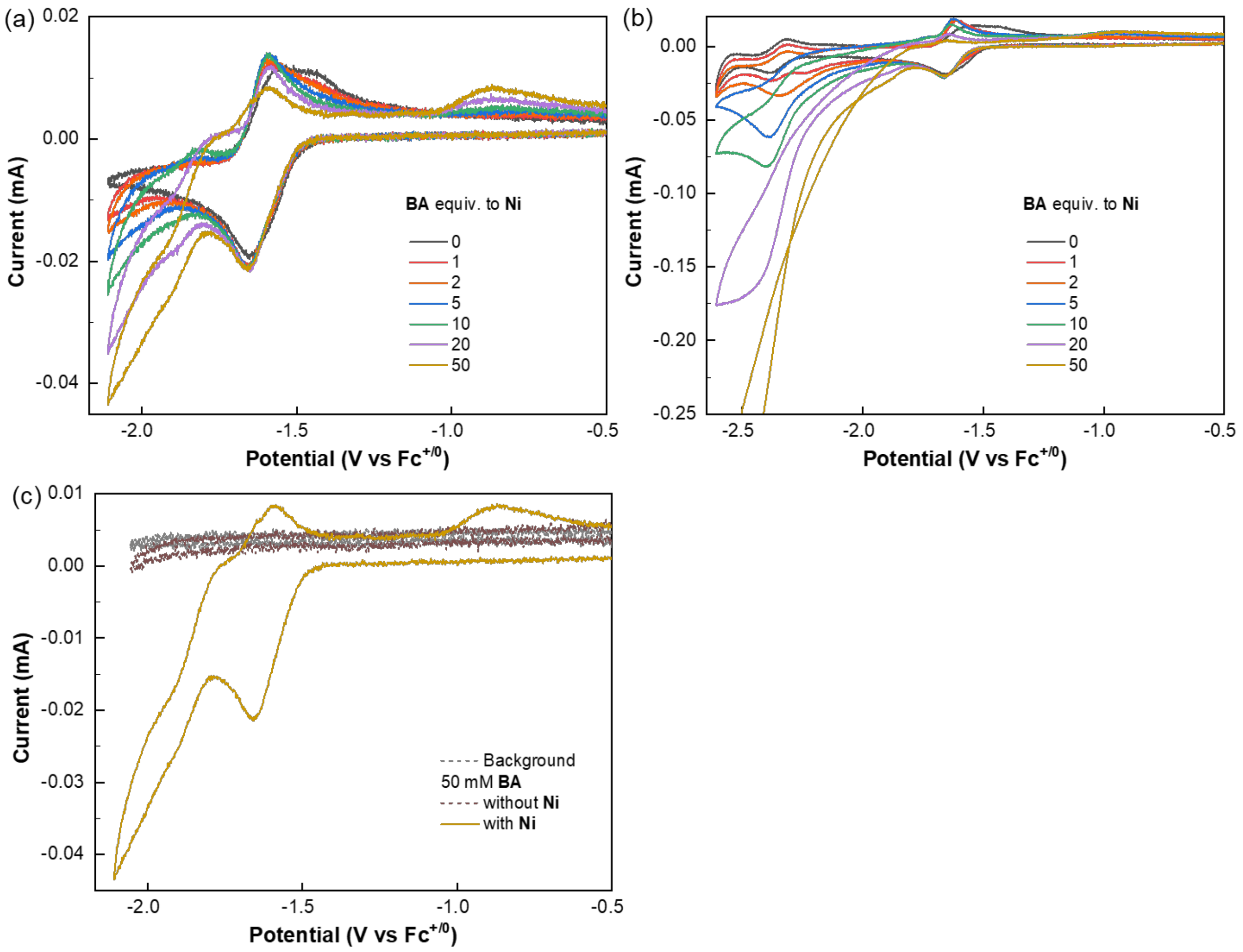

Fig. S5 (a,b) CVs of **Ni** upon addition of **BA** under potentials ranging (a) from –2.0 to –0.5 V_{Fc} and (b) from –2.5 to –0.5 V_{Fc}. (c) CVs at glassy carbon electrode in an electrolyte blank (gray dotted line), containing **BA** (wine dotted line) and containing **BA** and **Ni** (yellow plain line).

*3.1.2.4 **Ni** with **1** and **BA***

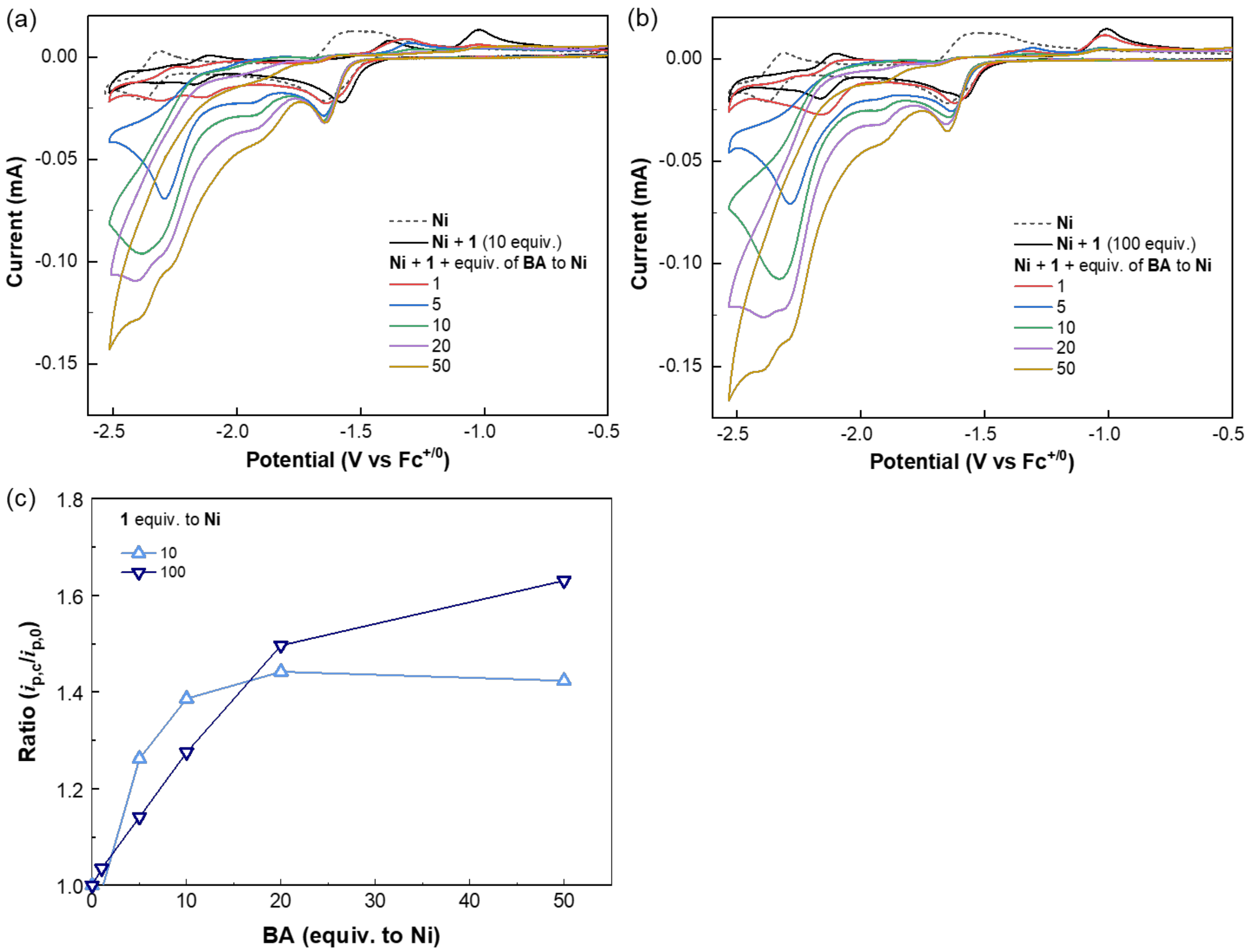

Fig. S6 CVs of **Ni** with **1** upon addition of **BA**: [**1**] = (a) 10 and (b) 100 mM. (c) Ratio of the cathodic peak current at –1.65 V_{Fc} upon addition of **BA** over the one in the absence of **BA** (data from (a) and (b)).

*3.1.3 Electrochemical behavior of **Ni** with **2***

*3.1.3.1 **Ni** with **2***

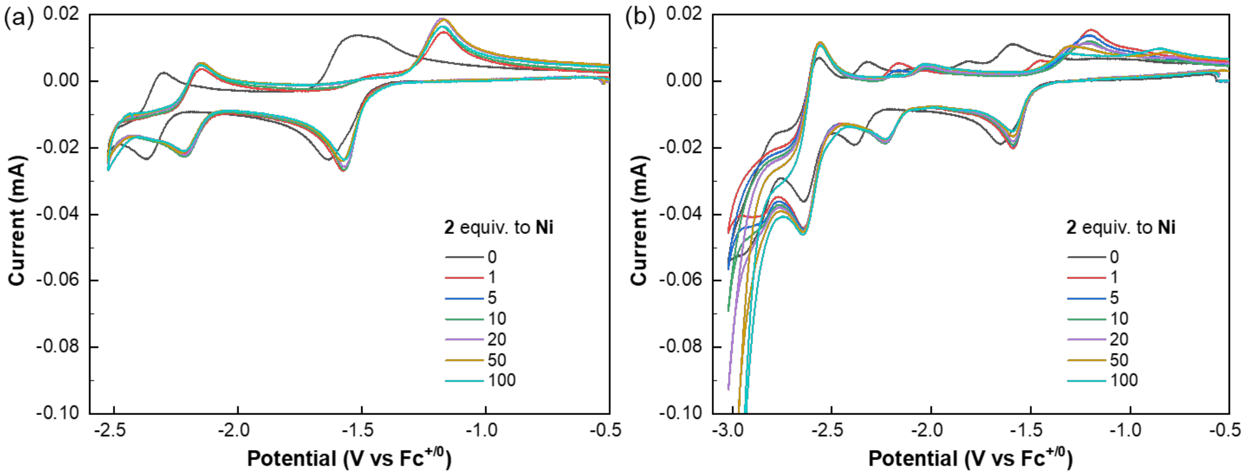

Fig. S7 CVs of **Ni** upon addition of **2** under potentials ranging (a) from –2.5 to –0.5 V_{Fc} and (b) from –3.0 to –0.5 V_{Fc}.

3.1.3.2 ***Ni*** *with* ***2*** *and* ***BA***

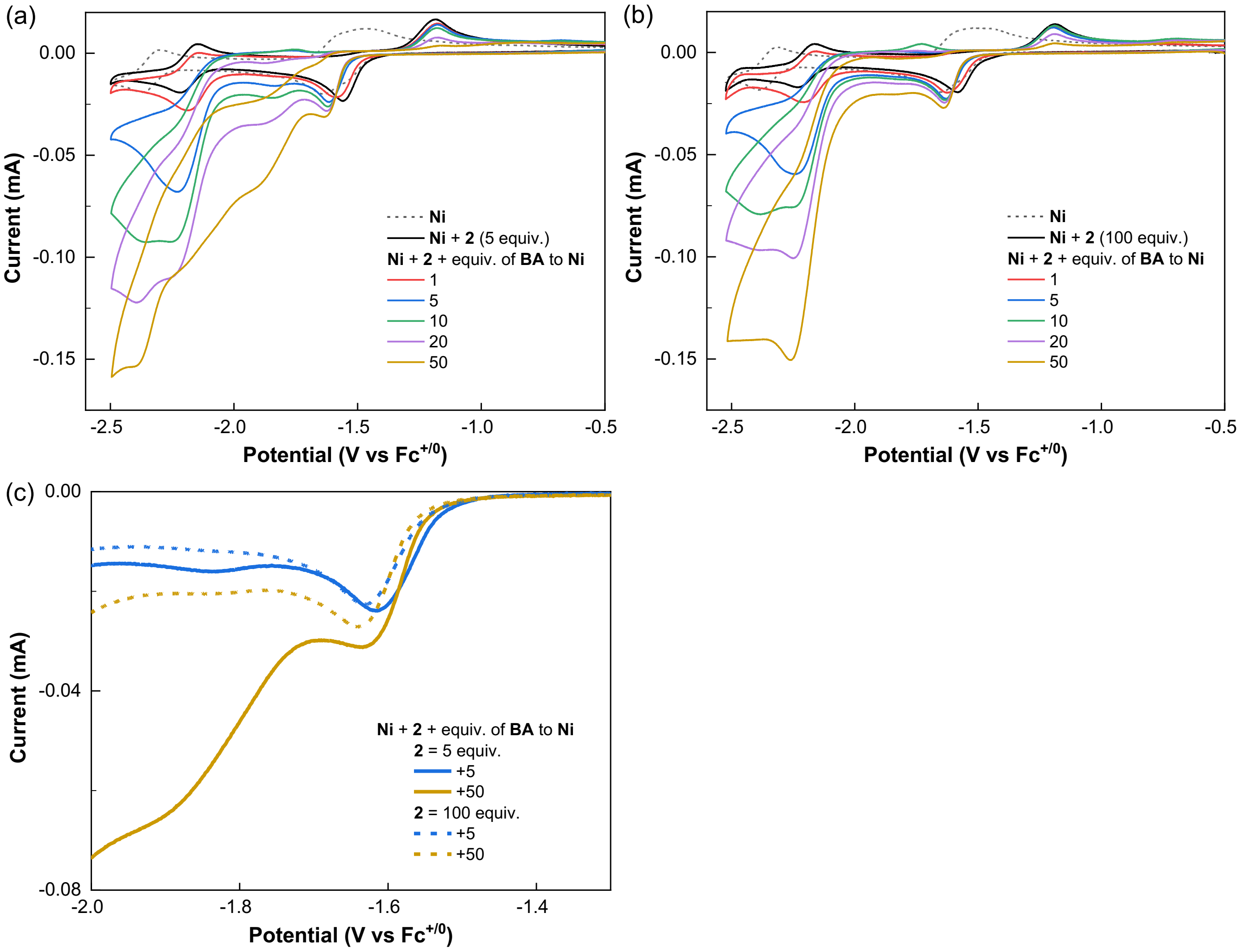

Fig. S8 CVs of **Ni** with **2** upon addition of **BA**: [**2**] = (a) 5 and (b) 100 mM. (c) Comparison of CVs (data from (a) and (b)) upon addition of **BA** (5 and 50 mM for blue and yellow lines, respectively) using different initial concentrations of **2** (5 and 100 mM for plain and dashed lines, respectively).

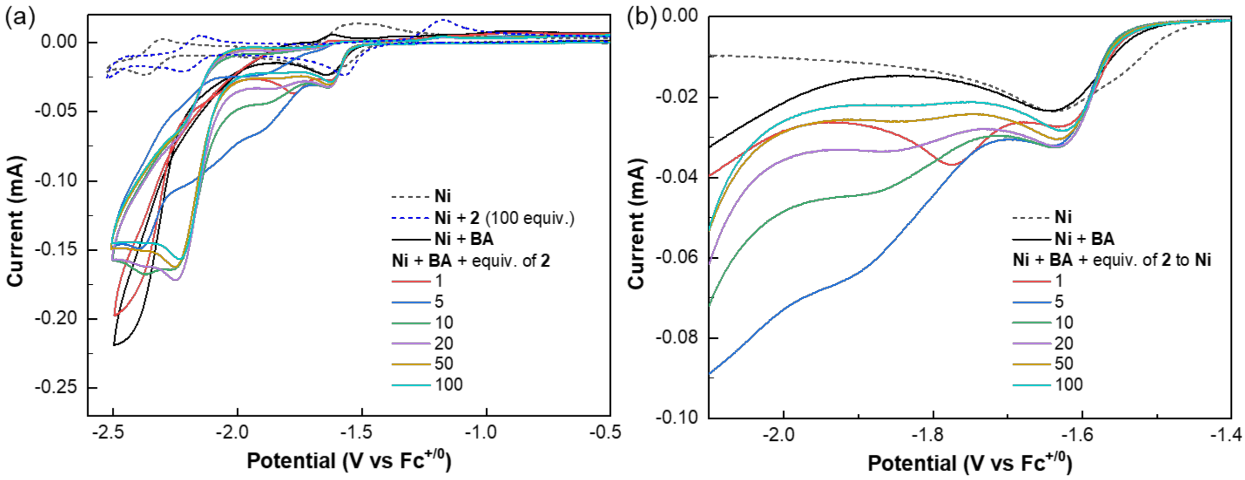

Fig. S9 CVs of **Ni** with **BA** upon addition of **2** ([**BA**] = 50 mM).

*3.1.3.3 **Ni** with **2** and **TFA***

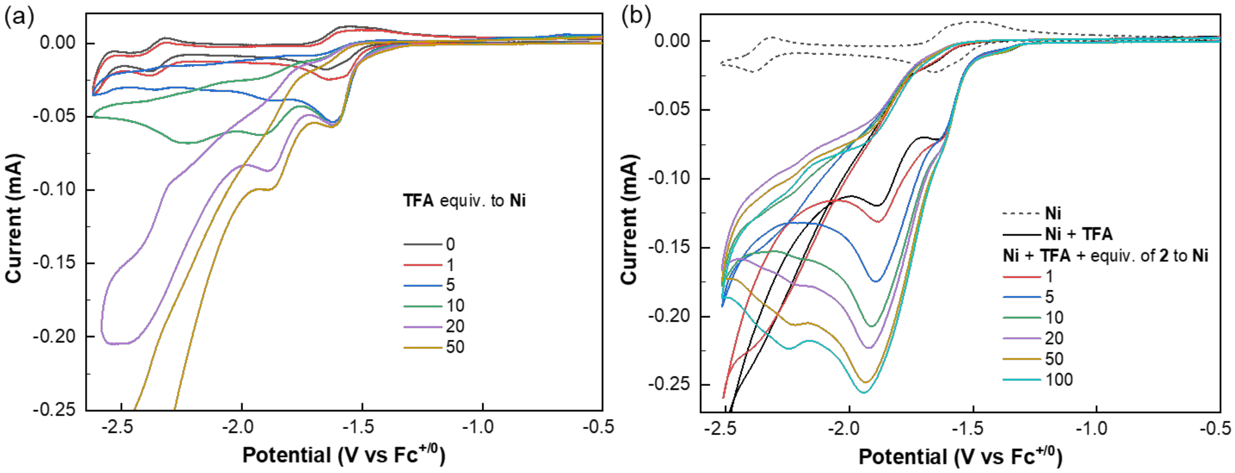

Fig. S10 CVs of **Ni**: (a) upon addition of **TFA** and (b) with **TFA** upon addition of **2** ([**TFA**] = 50 mM).

3.2 Electrochemical hydrogenation of alkynes

3.2.1 Screening of conditions

Table S2. Electrolysis optimization. Conditions unless otherwise stated: [**Ni**] = 1 mM; DMF; 0.1 M *n*Bu_4NPF_6; 2.5 h. Yields, TON, and F.E. are given for the maximum value at time to full conversion. [a]*Applied potentials without iR correction; iR-corrected values estimated in section* 3.2.2. [b]*Electrolysis without **Ni**.* [c]*Electrolysis using* $[Ni(MeCN)_6](BF_4)_2$ *instead of **Ni**.* [d]*H_2-saturated electrolyte.* [e]*Z/E = 96:4.* [f]*Z/E = 95:5.*

[**1**] (mM)	[**BA**] (mM)	E_{app}[a] (V *vs*. $Fc^{+/0}$)	Ar flow rate ($mL \cdot min^{-1}$)	Conversion (%)	Yield (%)	TON	F.E. (%)
1	50	–1.93	1	>99	59.8	0.6	38.6
5				>99	41.2	2.1	36.6
10				>99	57.8	5.8	32.5
20				62.8	27.3	5.5	15.4
100				14.3	4.4	4.4	9.5
10	0	–1.93	1	1.8	<1	<1	<1
	5			19.6	6.6	0.7	1.8
	50			>99	57.8	5.8	32.5
	100			>99	68.4 ± 5.7	6.8 ± 0.6	53.5 ± 3.0
	200			>99	75.3	7.5	68.8
	500			>99	83.6	8.4	58.1
10[b]	100	–1.93	1	4.4	<1	<1	<1
10[c]	100	–1.93	1	6.4	2.1	0.2	3.4
10	0	–1.93	0[d]	3.4	<1	<1	<1
10	100	–1.68	1	>99	45.7 ± 2.5[e]	5.3 ± 0.5	39.8 ± 3.3
		–1.93		>99	68.4 ± 5.7[f]	6.8 ± 0.6	53.5 ± 3.0
		–2.28		>99	72.1 ± 3.2[e]	7.2 ± 0.3	29.5 ± 5.1
10	100	–1.93	1	>99	68.4 ± 5.7	6.8 ± 0.6	53.5 ± 3.0
			10	>99	83.0 ± 4.1	8.3 ± 0.4	85.0 ± 3.2
	[**TFA**] (mM)						
10	100	–1.93	1	52.9	37.5	3.8	26.7
	[H_2O] (wt%)						
10	5	–1.93	1	4.5	1.0	0.1	1.8

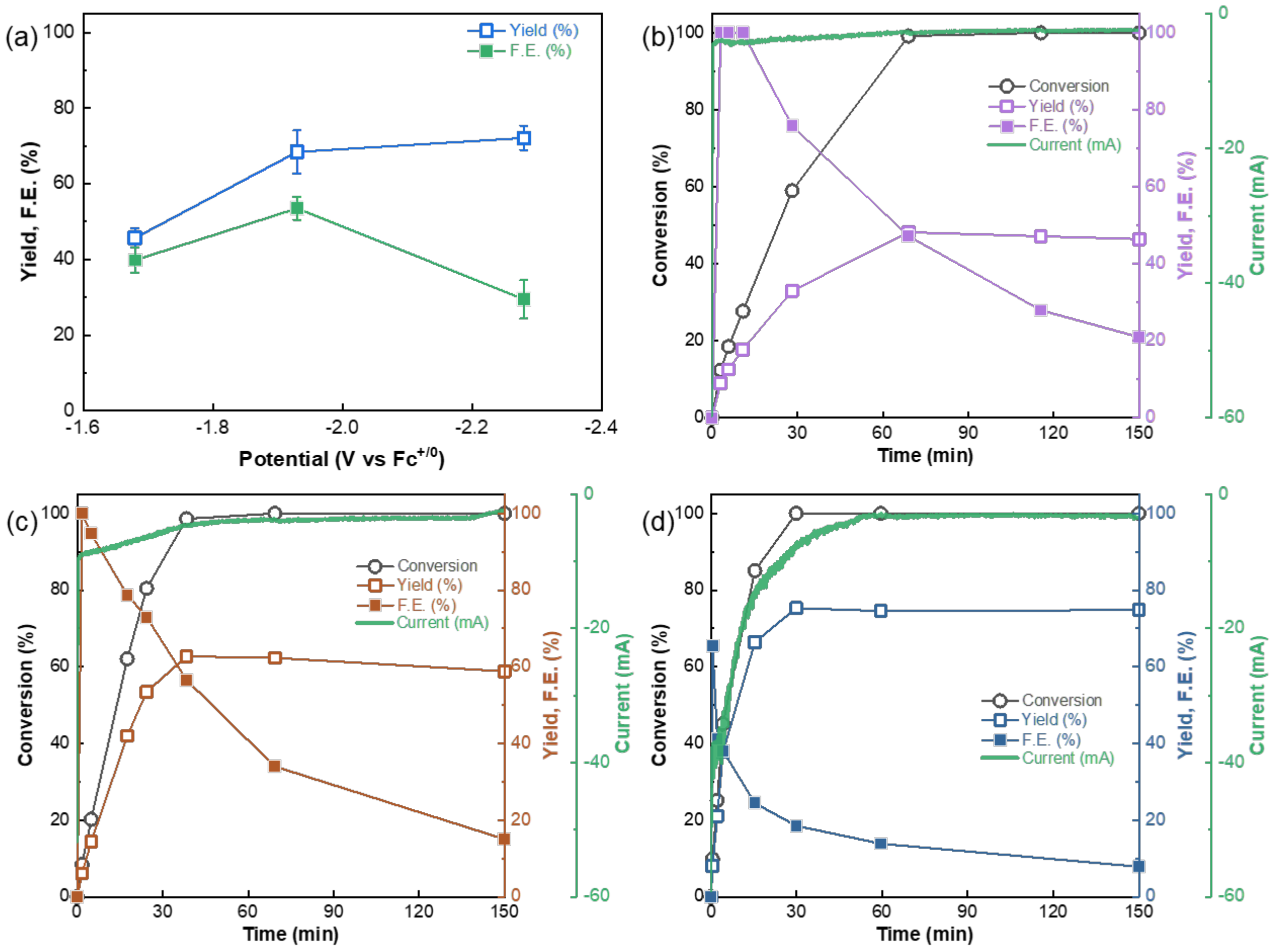

Fig. S11 (a) Yield and F.E. in **1**H$_2$ at time to full conversion of **1**, as a function of applied potential. Time-resolved current, conversion of **1**, yield and F.E. in **1**H$_2$ at applied potential of (b) –1.68, (c) –1.93 and (d) –2.28 V_{Fc}. Conditions: [**Ni**] = 1 mM; [**1**] = 10 mM; [**BA**] = 100 mM; DMF; 0.1 M *n*Bu$_4$NPF$_6$.

Plotting the yield and faradaic efficiency in **1**H$_2$ as a function of the applied potential (Fig. S11a) shows an increase of the yield up to a plateau value (ca 70%) as potential becomes more negative and a F.E. that compromises at the most negative potential. The increase in olefin yield with increasing overpotential (more negative potentials) may result from a faster turnover frequency towards the desired electrocatalytic transformation outcompeting (non-redox) side-reactions. On the other side, the decrease in F.E. at the most negative potential indicates a larger share of side reductions, most likely hydrogen evolution. This observation is in line with the proposed hypothesis of Ni-hydride species forming at more negative potentials (see section *3.4*) and from which electrocatalytic hydrogen evolution can proceed. The observed trade-off between yield and F.E. initially brought us to select an intermediate potential for screening the electro-conversion of alkyne substrates.

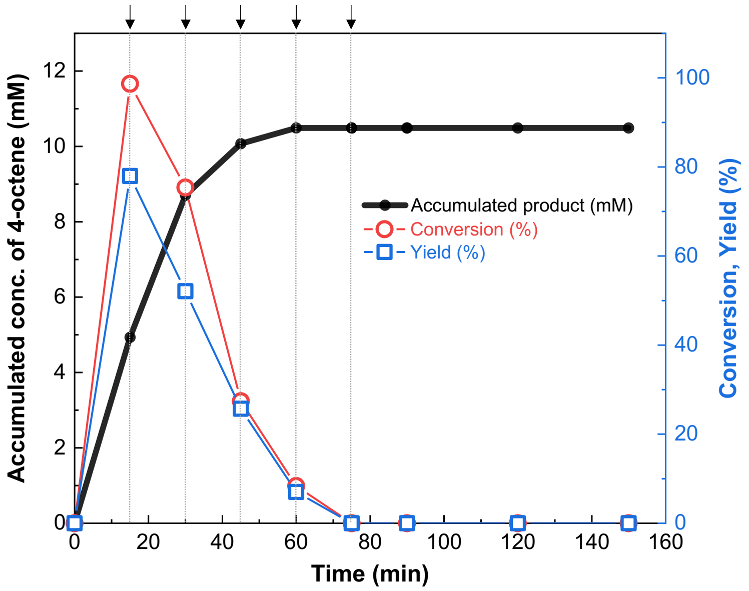

Fig. S12 Conversion of **1** and yield in **1**H_2 for each portion of added **1** and total cumulated concentration in **1**H_2. Conditions: [**Ni**] = 1 mM; **1** = 25 µmol (initial amount; 5 mM); [**BA**] = 100 mM; DMF; 0.1 M *n*Bu_4NPF_6; –1.93 V_{Fc}; addition of 25 µmol of **1** (5 mM) every 15 min.

3.2.2 iR-corrected potentials applied in electrolysis

In our experimental electrolytic setup (two-compartment cell split by a P3 frit), the application of iR compensation during electrolysis led to an oscillating behavior. For that reason, electrolyses were performed without compensating for ohmic drop. Nevertheless, an estimation of the potentials corrected from ohmic drop can be obtained using the ohmic drop measured prior to electrolysis (R_{cell}), the potential applied during electrolysis (E_{app}) and the current averaged during electrolysis (<i>).

An estimate of the iR-corrected potential is then given by: $E_{app,corr} = E_{app} – \langle i \rangle \cdot R_{cell}$. The corresponding values are reported in Table S3. We note that these $E_{app,corr}$ values represent conservative negative estimates of the potentials applied at initial time, since the magnitude in current decays as the electrolyses proceed and the alkyne is consumed.

Table S3. Ohmic drop-corrected potentials estimated for electrolyses. Conditions: [**Ni**] = 1 mM; [**1**] = 10 mM; [acid] = 100 mM; DMF; 0.1 M nBu$_4$NPF$_6$; time to full conversion. [a]*Electrolysis using [Ni(MeCN)$_6$](BF$_4$)$_2$ instead of* ***Ni****.* [b]*5 wt% H_2O.*

E_{app} (V vs $Fc^{+/0}$)	Acid	R_{cell} (Ω)	<i> (mA)	$E_{app,\ corr}$ (V vs $Fc^{+/0}$)
–1.68	**BA**	39.6 ± 2.7	− 3.2 ± 0.1	–1.55
–1.93	**BA**	56.4 ± 2.7	–4.4 ± 0.3	− 1.68
	**BA**[a]	104.7	–2.2	− 1.70
	**TFA**	77.9	–3.5	–1.66
	H_2O[b]	59.2	–2.4	–1.79
–2.28	**BA**	73.3 ± 3.7	–6.5 ± 0.3	–1.80

3.2.3 Electrolysis control experiments

3.2.3.1 Extended electrolysis

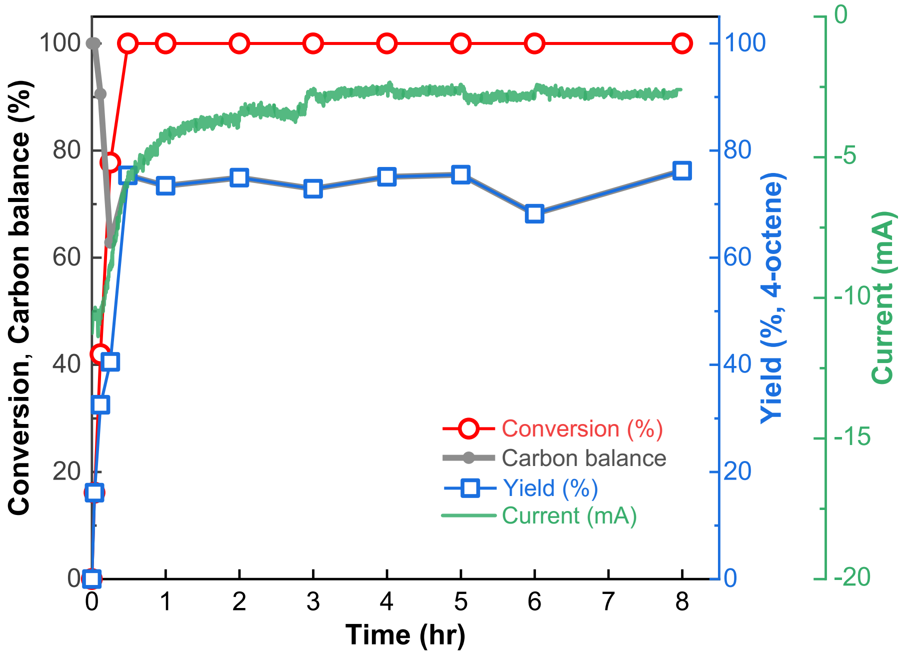

Fig. S13 Current, conversion of **1**, yield in **1**H_2 and carbon balance during long-term electrolysis. Conditions: [**Ni**] = 1 mM; [**1**] = 10 mM; [**BA**] = 100 mM; DMF; 0.1 M *n*Bu_4NPF_6; –1.93 V_{Fc}.

3.2.3.2 With olefin substrate

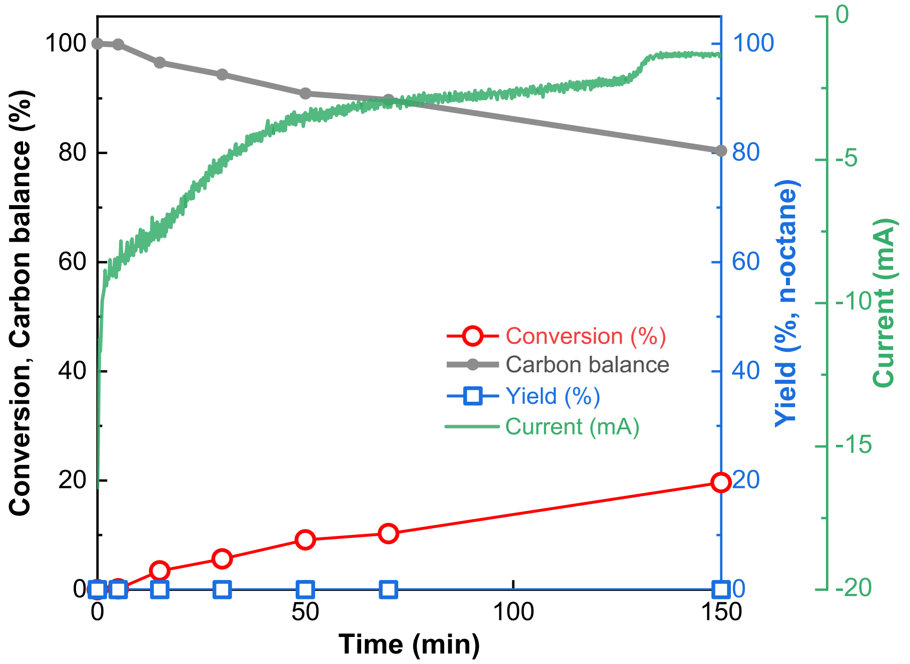

Fig. S14 Current, conversion of **1**H_2, yield in **1**H_4 and carbon balance during electrolysis. Conditions: [**Ni**] = 1 mM; [(*Z*)-**1**H_2] = 10 mM; [**BA**] = 100 mM; DMF; 0.1 M *n*Bu_4NPF_6; –1.93 V_{Fc}.

The electrolysis using an olefin ((*Z*)-octene; (Z)-**1**H_2) instead of an alkyne substrate under conditions otherwise identical to our standard ones (Fig. S14) shows minor conversion of the olefin and no evolution of the alkane product (**1**H_4 below traces). This result demonstrates that, under these conditions, our system is not active towards olefin hydrogenation and has thus intrinsic selectivity for the semihydrogenation of alkyne.

3.2.3.3 Without and with applied potential

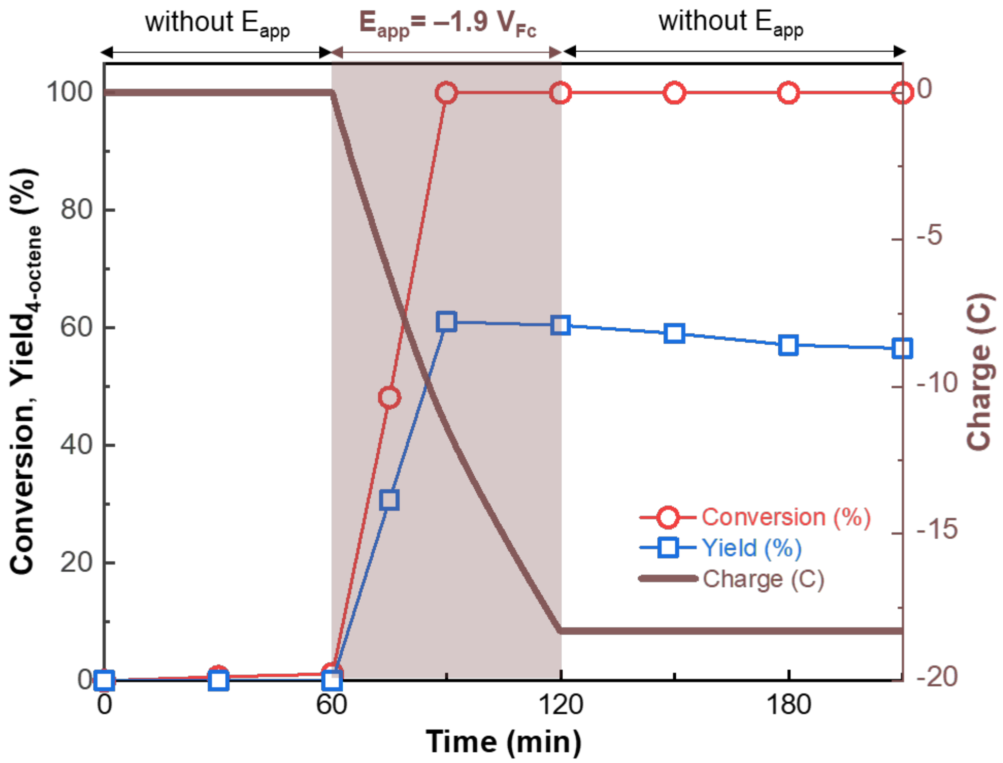

Fig. S15 Charge, conversion of **1** and yield in **1**H_2 without and with applied potential. Conditions: [**Ni**] = 1 mM; [**1**] = 10 mM; [**BA**] = 100 mM; DMF; 0.1 M $n$$Bu_4NPF_6$; –1.93 V_{Fc}.

3.2.3.4 Under H_2

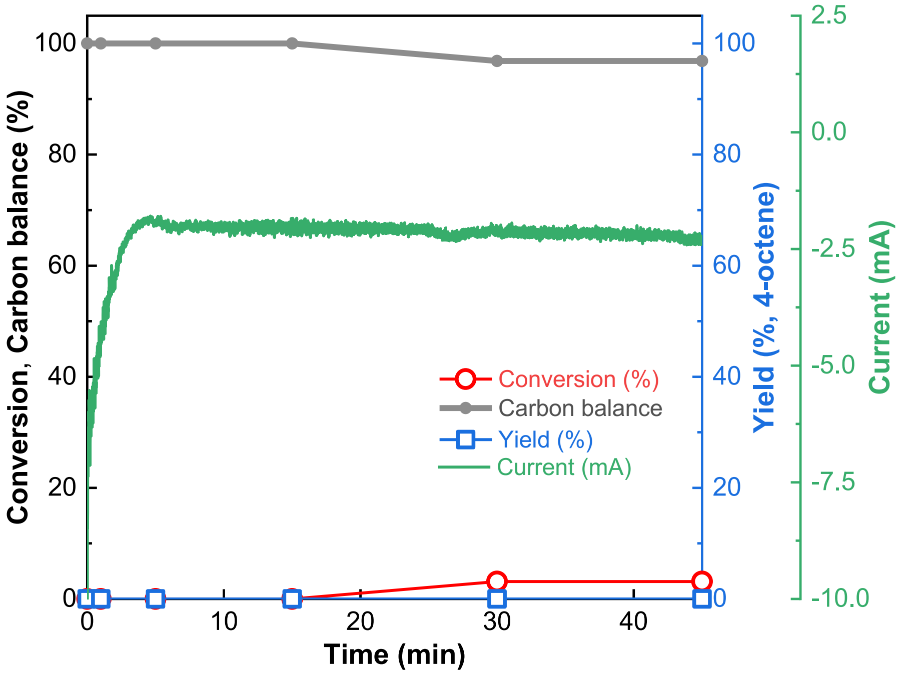

Fig. S16 Current, conversion of **1**, yield in **1**H_2 and carbon balance during electrolysis in H_2-saturated electrolyte. Conditions: [**Ni**] = 1 mM; [**1**] = 10 mM; DMF; 0.1 M *n*Bu_4NPF_6; –1.93 V_{Fc}; H_2 bubbling.

The electrolysis in an electrolyte saturated with H_2 but exempt of acid (**BA**) under conditions otherwise identical to our standard ones shows only minor conversion of the alkyne **1** (3.4%), with olefin evolution below traces (Fig. S16). This result further assesses that, in our system, the nature of the conversion process is an electrocatalytic hydrogenation and not an electrochemically-assisted hydrogenation.

3.2.3.5 Using [Ni(MeCN)$_6$](BF$_4$)$_2$

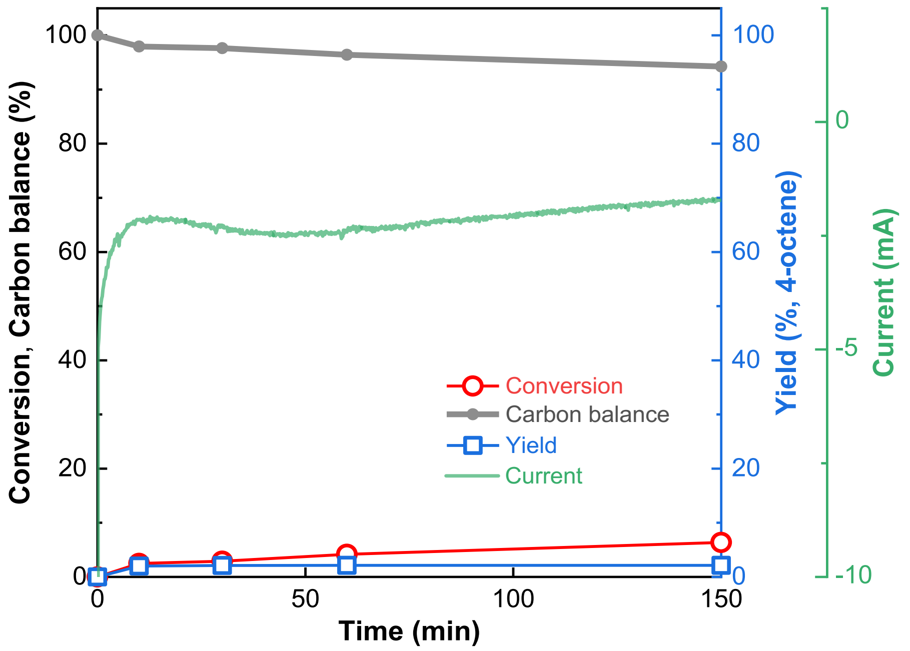

Fig. S17 Current, conversion of **1**, yield in **1**H$_2$ and carbon balance during electrolysis with [Ni(MeCN)$_6$](BF$_4$)$_2$. Conditions: [Ni(MeCN)$_6$](BF$_4$)$_2$ = 1 mM; [**1**] = 10 mM; [**BA**] = 100 mM; DMF; 0.1 M *n*Bu$_4$NPF$_6$; –1.93 V$_{Fc}$.

The electrolysis of **1** using [Ni(MeCN)$_6$](BF$_4$)$_2$ under conditions otherwise identical to that using **Ni** resulted in 6% conversion with 2% yield and 3% F.E. (Fig. S17).

3.2.3.6 With post-activity electrodes

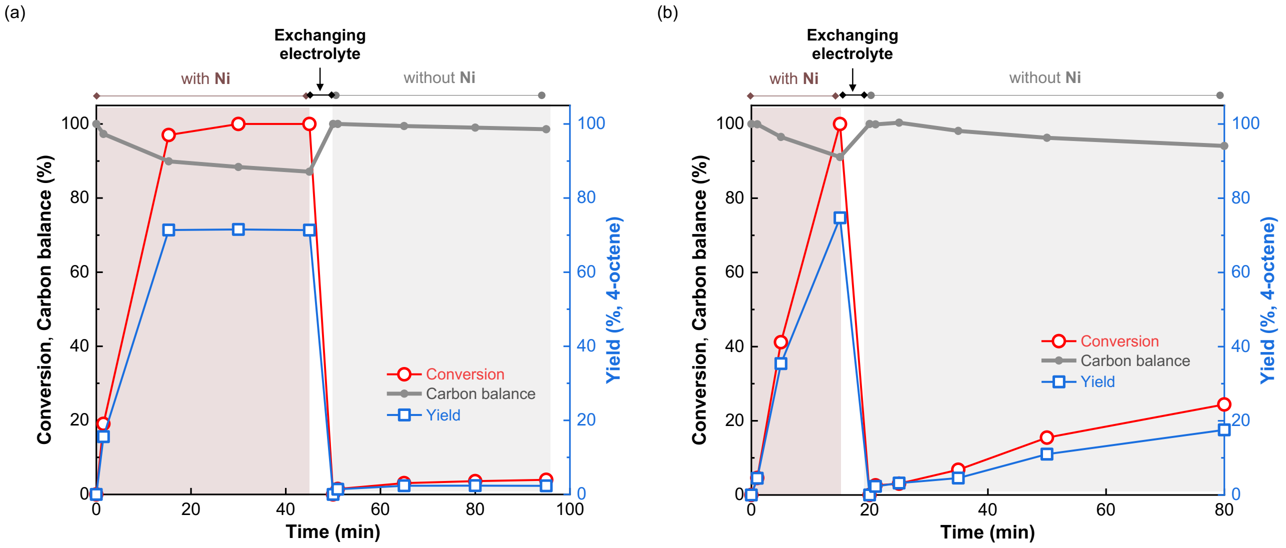

Fig. S18 Conversion of **1**, yield in **1**H_2 and carbon balance during electrolyses in the following conditions: [**Ni**] = 1 mM (first run; mauve areas) then 0 mM (second run; grey areas); [**1**] = 10 mM (both runs); [**BA**] = 100 mM (both runs); DMF; 0.1 M nBu$_4$NPF$_6$; –1.93 V_{Fc}.

At the end of an electrolysis run under our standard conditions for 45 min (Fig. S18a, mauve area), the working electrode (carbon foam) was quickly disconnected, taken out of the solution and was not rinsed. This procedure is aimed to prevent the re-dissolution of any Ni deposits that can occur in the absence of (cathodic) applied potential and lead to false negative rinse-tests.[4] The final electrolyte solution was removed from both chambers of the electrolysis cell (also not rinsed) and replaced by fresh electrolyte containing **1** and **BA** but exempt of **Ni**. The non-rinsed, previously used working electrode was plunged in the fresh electrolyte and a new electrolysis performed. This second electrolysis produces only traces of conversion (3.9 % at 45 min; Fig. S18a, grey area). A similar experiment but stopping the first electrolysis at 15 min (this duration being our shortest estimate to reach full alkyne conversion) produces a similar result (Fig. S18b), although with a remainder of activity in the second electrolysis (at a rate at least 20 times slower) likely due to the presence of active Ni complex in the remainder of initial electrolyte carried over by the non-rinsed electrode and cell.

We also note that no induction period is observed prior to activity during electrocatalytic runs with **Ni** (see Fig. S11,15), which would indicate the degradation of the molecular complex into another species responsible for catalysis.

Finally, in our first experiment (45 min electrolysis), we submitted the working electrode after the second electrolysis (withdrawn from the electrolyte following the same procedure as above) to digestion for ICP-MS analysis. The ICP-MS analysis shows the presence of Ni on the electrode in an amount of n_{elec}(Ni) = 0.1 µmol. For comparison, the amount engaged in the cathodic electrolyte of the first electrolysis run is n_i(Ni) = 5 µmol (5 mL at [**Ni**] = 1 mM). As the electrode

was not rinsed, such results can indicate a deposit of Ni strongly adsorbed on the electrode or Ni species dissolved in the remaining film of electrolyte covering the electrode. Although we cannot conclude on the exact speciation of these deposits (adsorbed molecular complexes, clusters or nanoparticles), SEM pictures of an electrode following an electrolysis under our standard conditions (including [**Ni**]) and disconnected as described above (Fig. S20) do not evidence Ni deposits, which suggest that these deposits would be of small size (< 10 nm).

Collectively, these results conclusively demonstrate that, if Ni deposits may form during electrolysis, these deposits are not the species responsible for electrocatalytic alkyne semihydrogenation in our conditions.

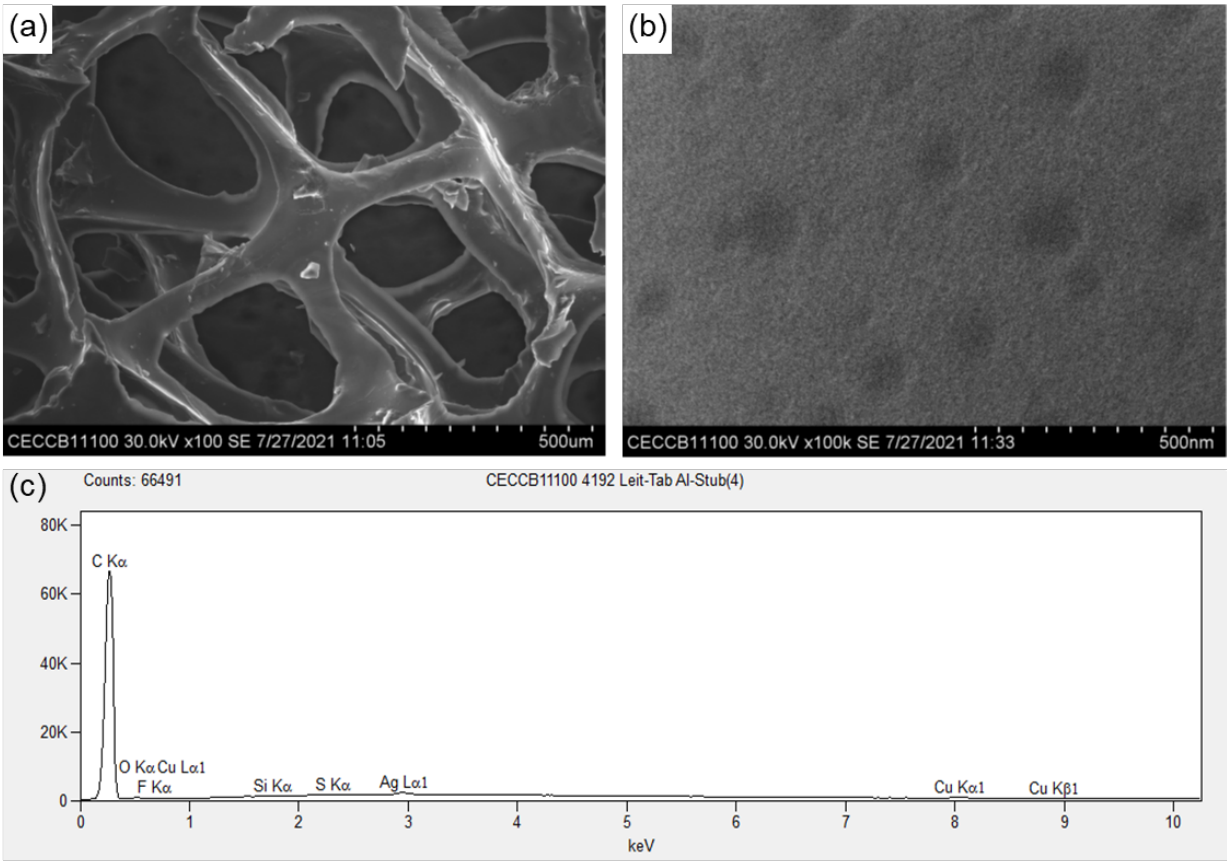

Element	Net intensity	Weight %	Atom %
C K	489085	94.5 ± 0.4	96.4 ± 0.4
O K	1815	3.3 ± 0.2	2.5 ± 0.2
F K	1345	1.4 ± 0.1	0.9 ± 0.0
Si K	0	0.0	0.0
S K	0	0.0	0.0
Ni K	506	0.0	0.0
Cu K	4416	0.4 ± 0.0	0.1 ± 0.0
Ag L	6350	0.3 ± 0.0	0.1 ± 0.0
**Total**		**100.0**	**100.0**

Fig. S19 SEM images (a)–(b) and EDX analysis (c) of a carbon form working electrode before electrolysis.

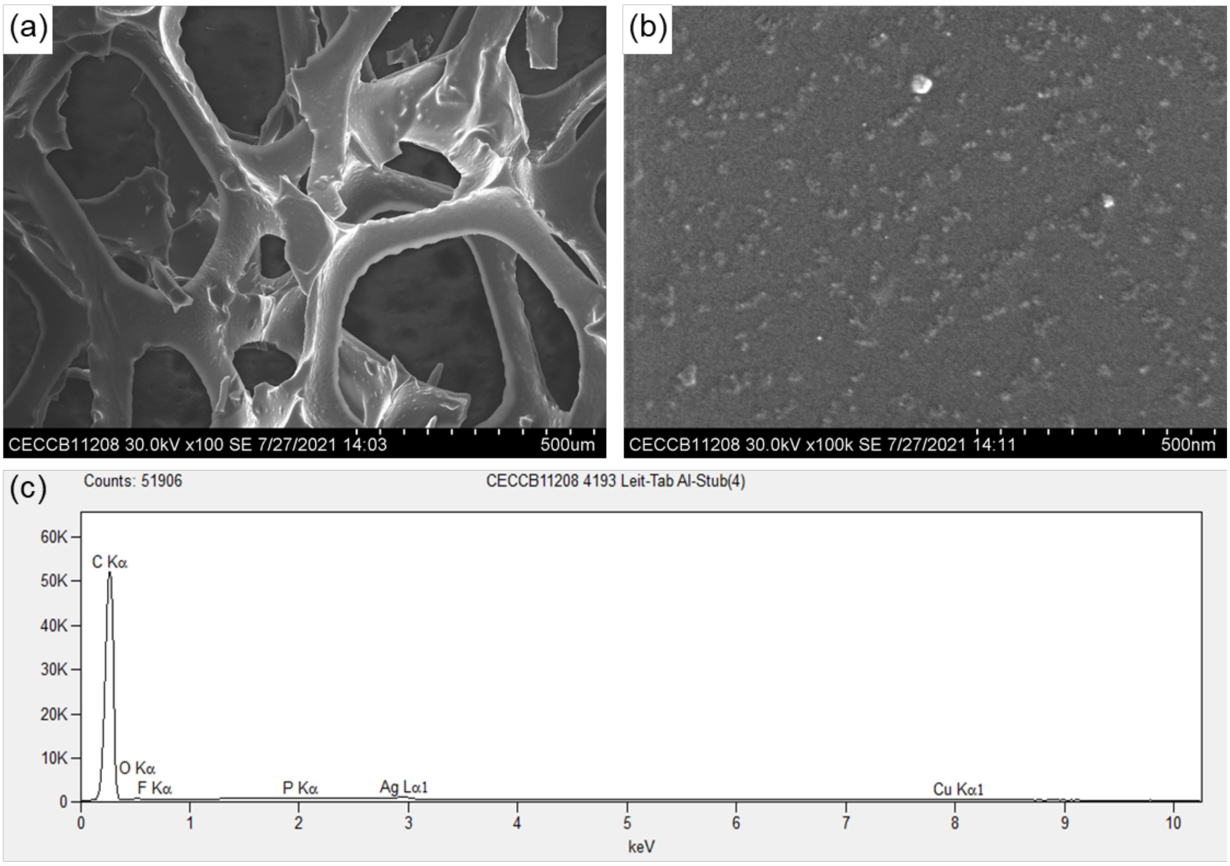

Element	Net intensity	Weight %	Atom %
C K	386156	88.0 ± 0.4	91.3 ± 0.4
O K	4634	8.3 ± 0.2	6.5 ± 0.1
F K	2867	3.3 ± 0.1	2.2 ± 0.0
P K	2720	0.1 ± 0.0	0.0
Ni K	15	0.0	0.0
Cu K	61	0.0	0.0
Ag L	5809	0.3 ± 0.0	0.0
**Total**		**100.0**	**100.0**

Fig. S20 SEM images (a)–(b) and EDX analysis (c) of a carbon form working electrode after electrolysis (conditions: [**Ni**] = 1 mM; [**1**] = 10 mM; [**BA**] = 100 mM; DMF; 0.1 M nBu$_4$NPF$_6$; –1.93 V$_{Fc}$; 2.5 h.).

3.2.4 Substrate scope

Table S4. Electrocatalytic hydrogenation of alkynes with **Ni** at room temperature ([**Ni**] = 1 mM; [**S**] = 10 mM; [**BA**] = 100 mM; DMF; 0.1 M *n*Bu_4NPF_6; E_{app} = –1.93 V_{Fc}; 2.5 h). [a]*Time to full conversion in parenthesis.* [b]*At time to full conversion.* [c]*Traces detected in GC-MS.* [d]*Dienes and monoenes observed.* [e]*Conversion and yields obtained by NMR.*

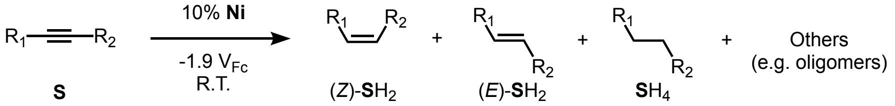

S	Substrate	Conversion[a] (%)	Yield **S**H_2[b] (%)	Z/E[b]	Yield **S**H_4[b] (%)	FE[b] (%)
**1**	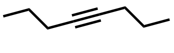	>99 (40)	68.4 ± 5.7	95:5	<1	53.5 ± 3.0
**2**	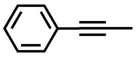	>99 (20)	84.9 ± 3.1	92:8	<1	59.3 ± 10.6
**3**	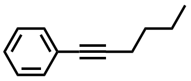	>99 (30)	93.3 ± 0.9	83:17	Traces[c]	51.8 ± 0.6
**4**	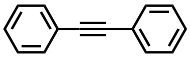	>99 (50)	83.4	61:39	<1	23.5
**5**	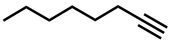	>99 (20)	38.9	-	n.d.	29.9
**6**	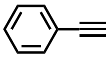	>99 (40)	38.4 ± 3.6	-	<1	24.4 ± 0.7
**7**	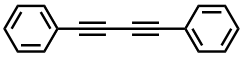	60.2[d]	45.0	n.d.	Traces[c]	20.5
**8**	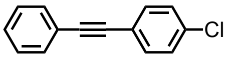	>99 (100)	85.5	70:30	Traces[c]	46.1
**9**	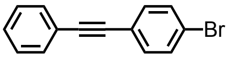	27.0	10.5	63:37	<1	4.8
**10**	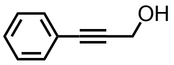	>99[e] (15)	93.5[e]	95:5	n.d.	97.9

1: 4-octyne

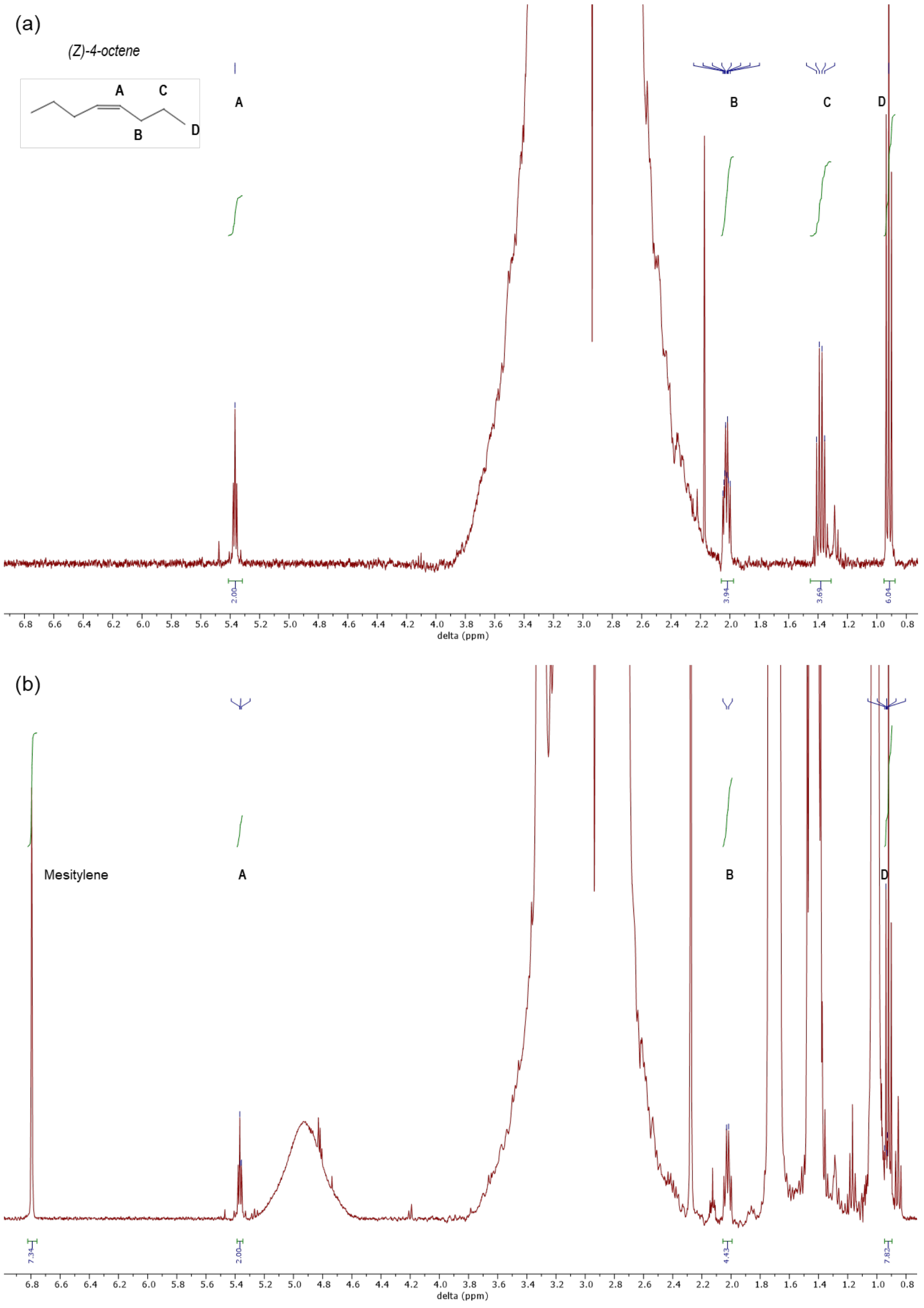

Fig. S21 ^{1}H NMR spectra of (a) (*Z*)-4-octene ((*Z*)-**1**H_2) standard in DMF and (b) electrolytic mixture after 60 min of electrolysis of 4-octyne (**1**).

(*Z*)-4-octene ((Z)-**1**H_2): δ_H = 5.37 (m, 2H; **H$_A$**), 2.06 – 1.97 (m, 4H; **H$_B$**), 1.45 – 1.32 (m, 4H; **H$_C$**), 0.92 (t, *J* = 7.5 Hz, 6H; **H$_D$**).

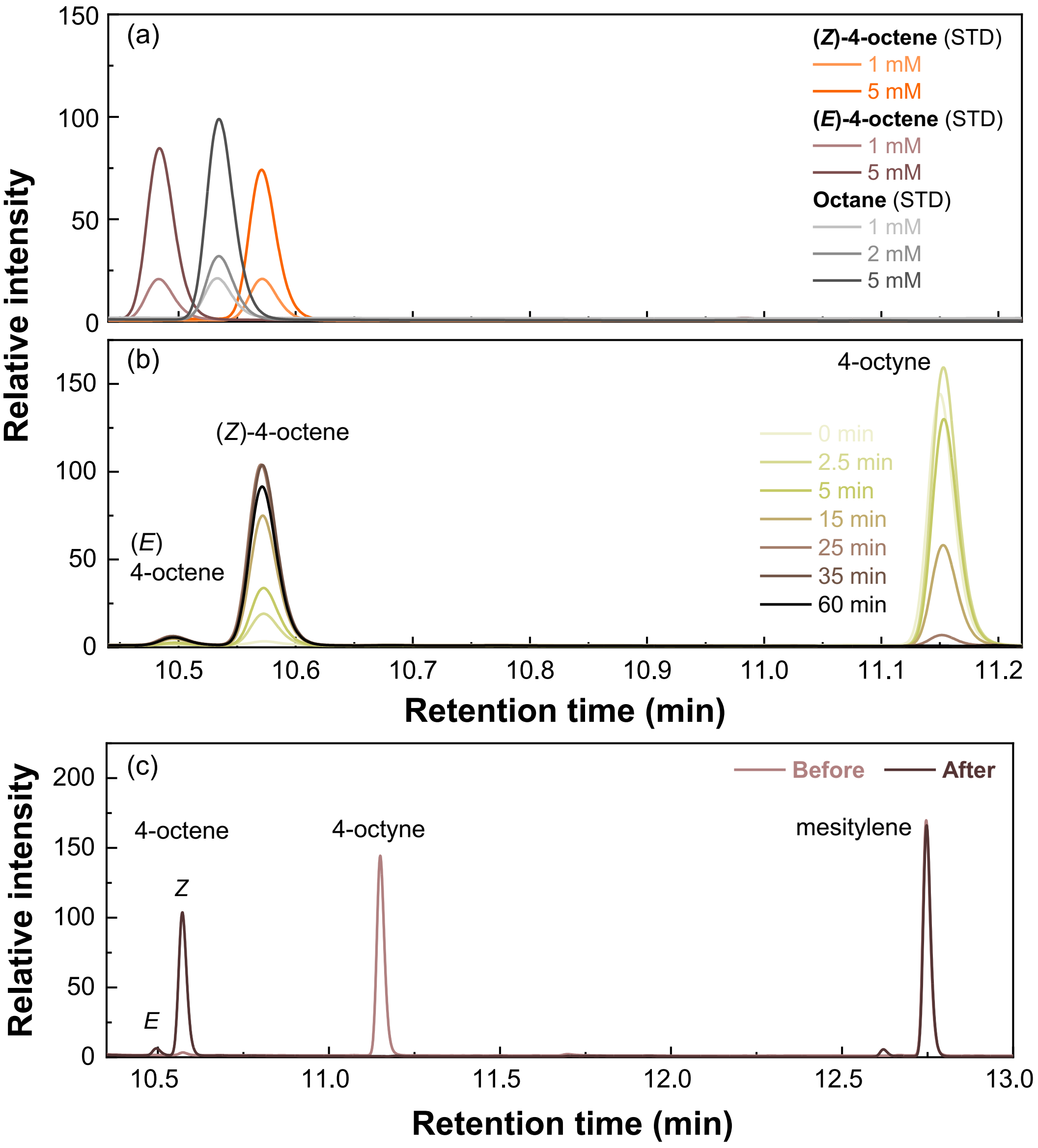

Fig. S22 GC-FID chromatograms: (a) Octane (**1**H_4), (*Z*)- and (*E*)-4-octene ((Z)- and (*E*)-**1**H_2) reference products in DMF, (b) electrolytic mixture at various times during electrolysis of 4-octyne (**1**) and (c) electrolytic mixture before and after electrolysis with quantification of 4-octyne (**1**) and 4-octene (**1**H_2) relative to mesitylene (internal standard).

2: 1-phenyl-1-propyne

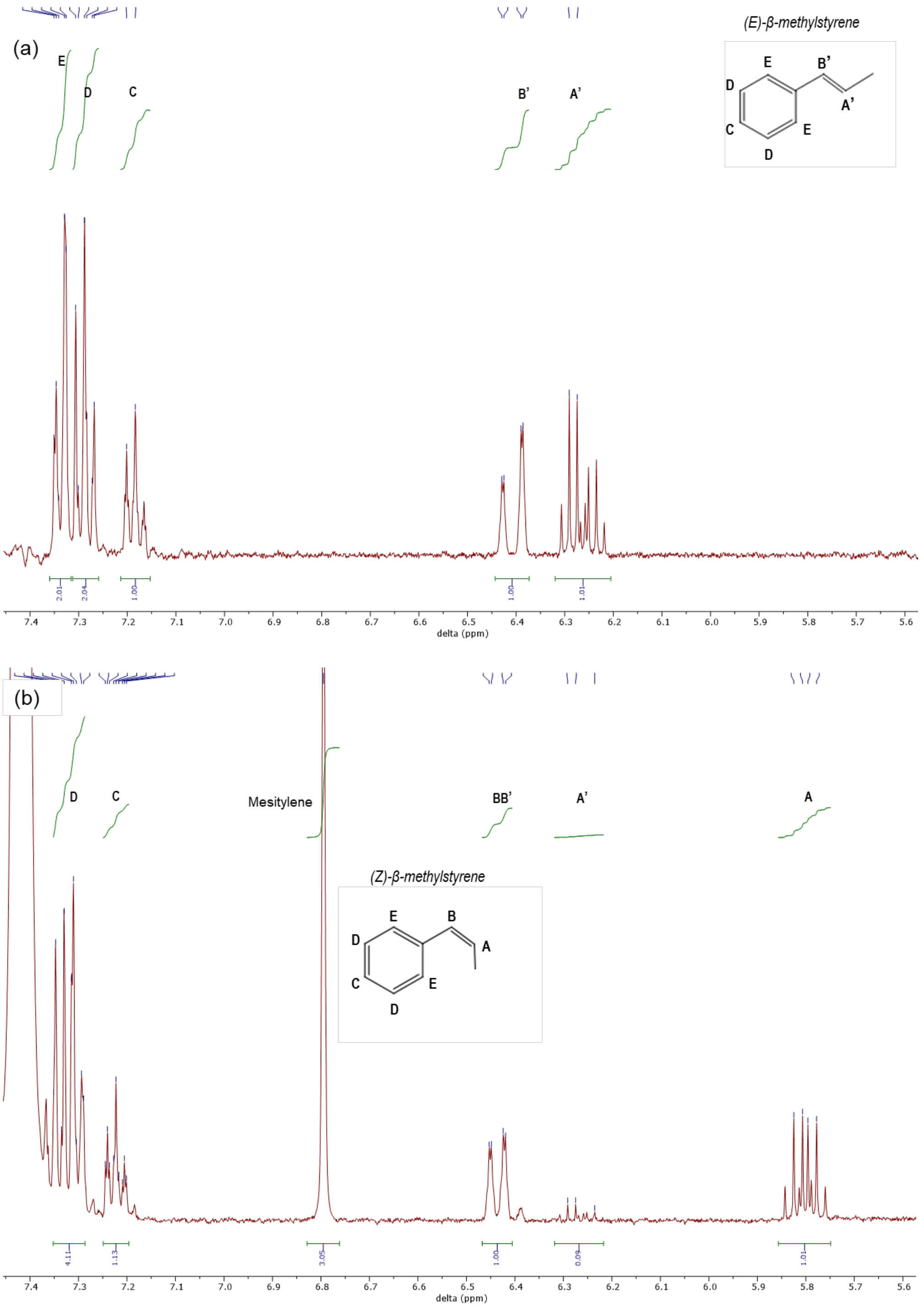

Fig. S23 ^{1}H NMR spectra of (a) (*E*)-*β*-methylstyrene ((*E*)-**2**H_2) standard in DMF and (b) electrolytic mixture after 20 min of electrolysis of 1-phenyl-1-propyne (**2**).

(*E*)-*β*-methylstyrene ((*E*)-**2**H_2): δ_H = 7.36 – 7.32 (m, 2H; **H$_E$**), 7.31 – 7.26 (m, 2H; **H$_D$**), 7.21 – 7.16 (m, 1H; **H$_C$**), 6.39 (dd, 1H; **H$_{B'}$**), 6.28 (dq, 1H; **H$_{A'}$**).

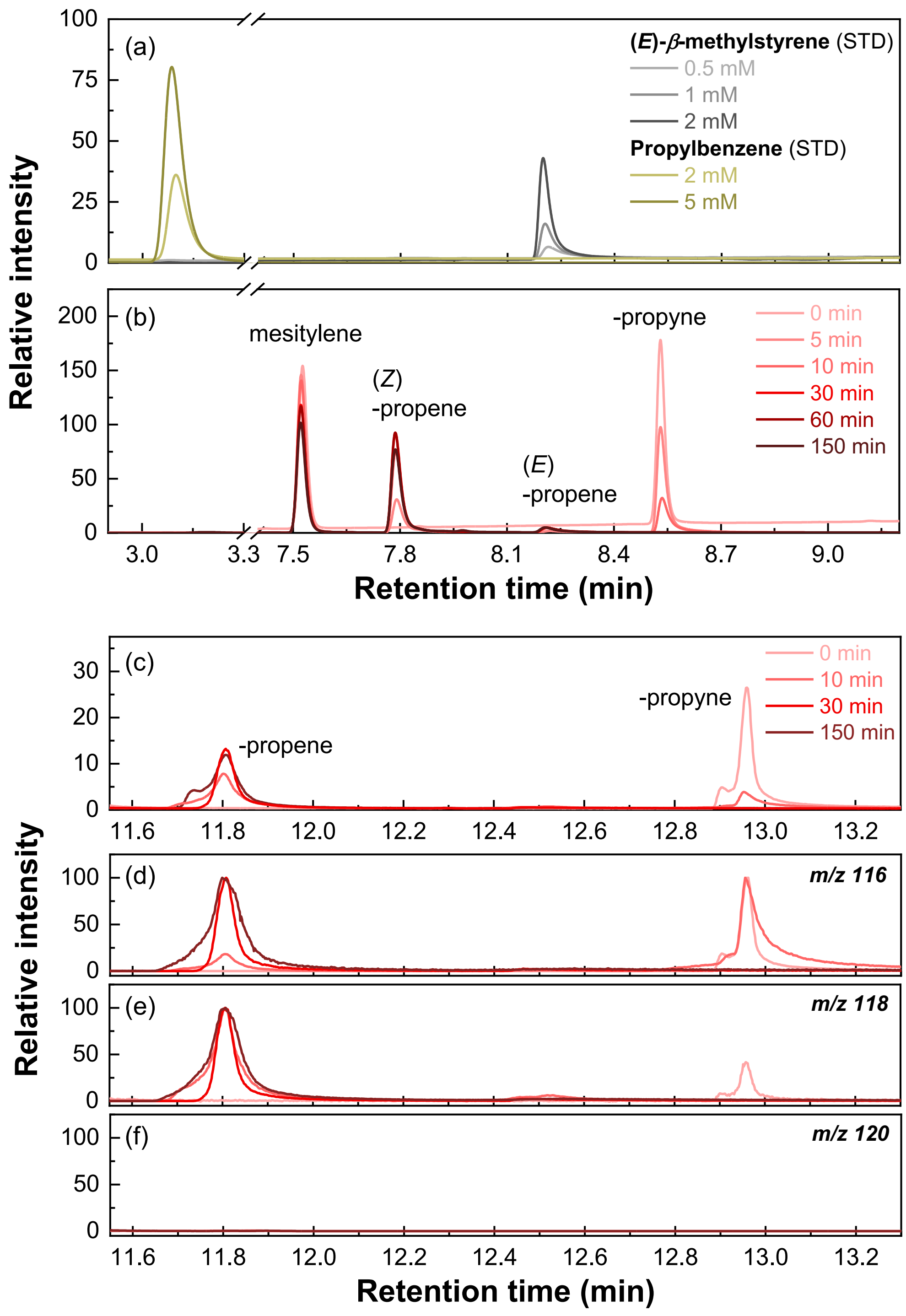

Fig. S24 (a) GC-FID chromatograms of propylbenzene (**2**H_4) and (*E*)-*β*-methylstyrene ((*E*)-**2**H_2) reference products in DMF. GC chromatograms of the electrolytic mixture during electrolysis of 1-phenyl-1-propyne (**2**, labelled -propyne) to *β*-methylstyrene (**2**H_2, labelled -propene): (b) GC-FID and (c-f) GC-MS.

3: 1-phenyl-1-hexyne

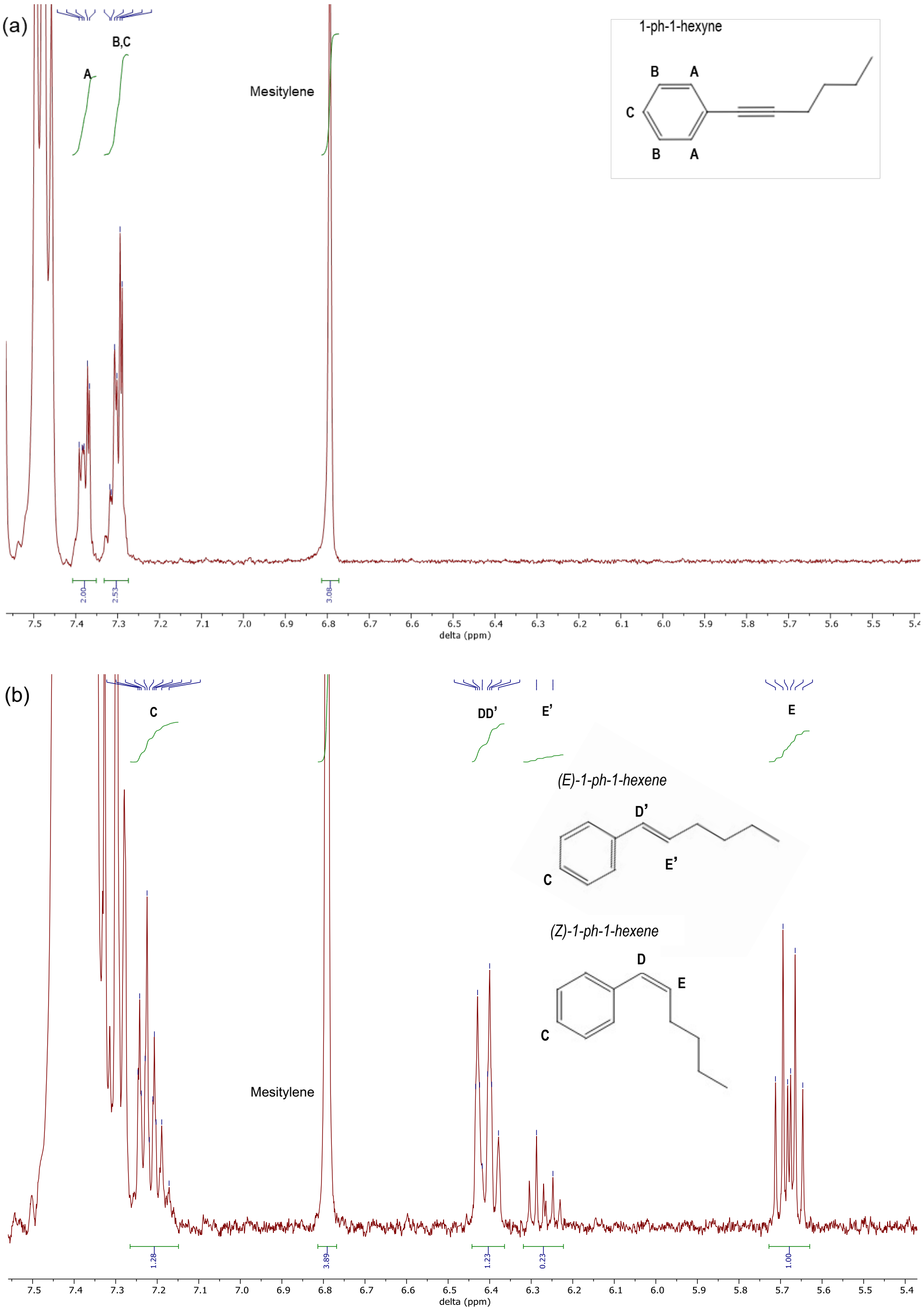

Fig. S25 ^{1}H NMR spectra of crude samples of the electrolytic mixture of 1-phenyl-1-hexyne (**3**): (a) before electrolysis and (b) after 30 min.

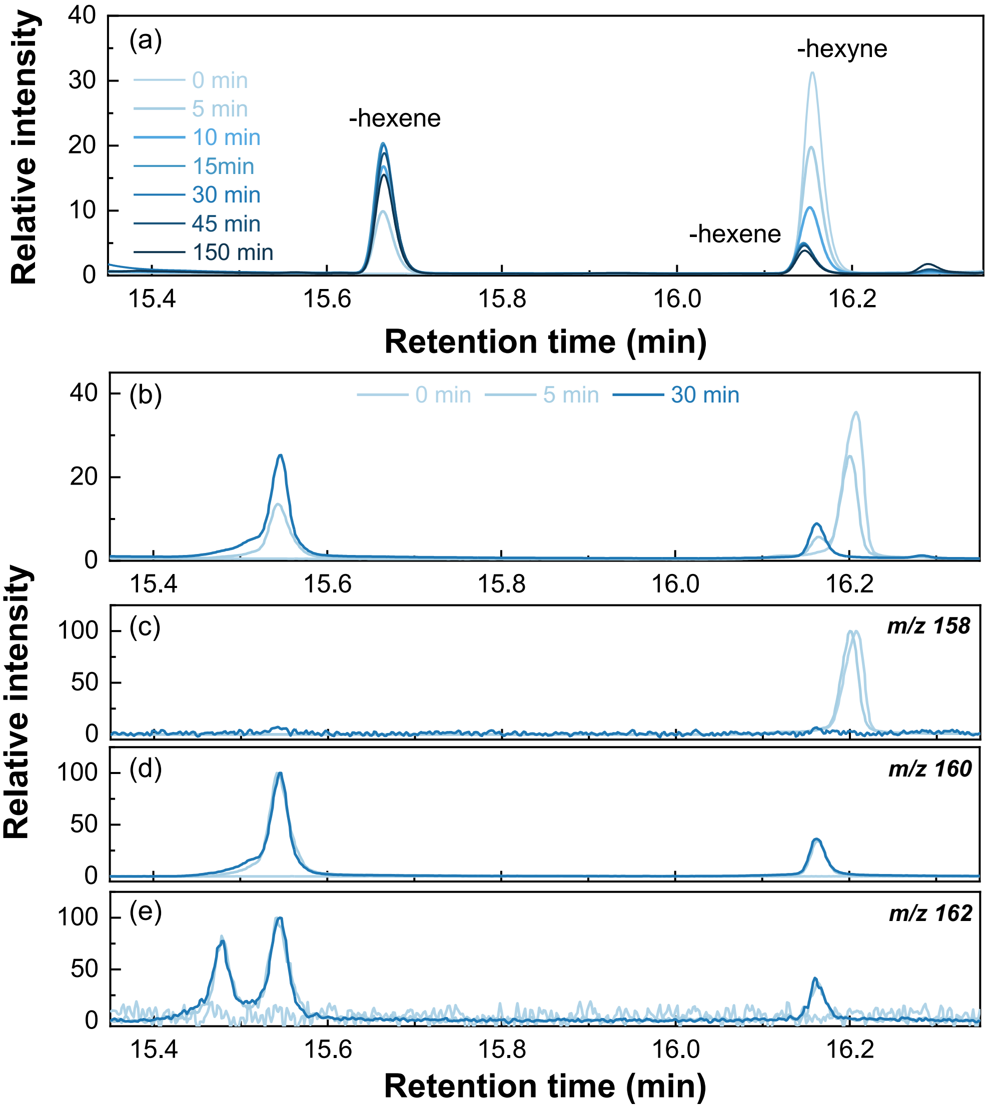

Fig. S26 GC chromatograms for hydrogenation of 1-phenyl-1-hexyne (**3**, labelled -hexyne) to 1-phenyl-1-hexene (**3**H_2, labelled -hexene) during electrolysis: (a) GC-FID and (b-e) GC-MS.

4: diphenylacetylene

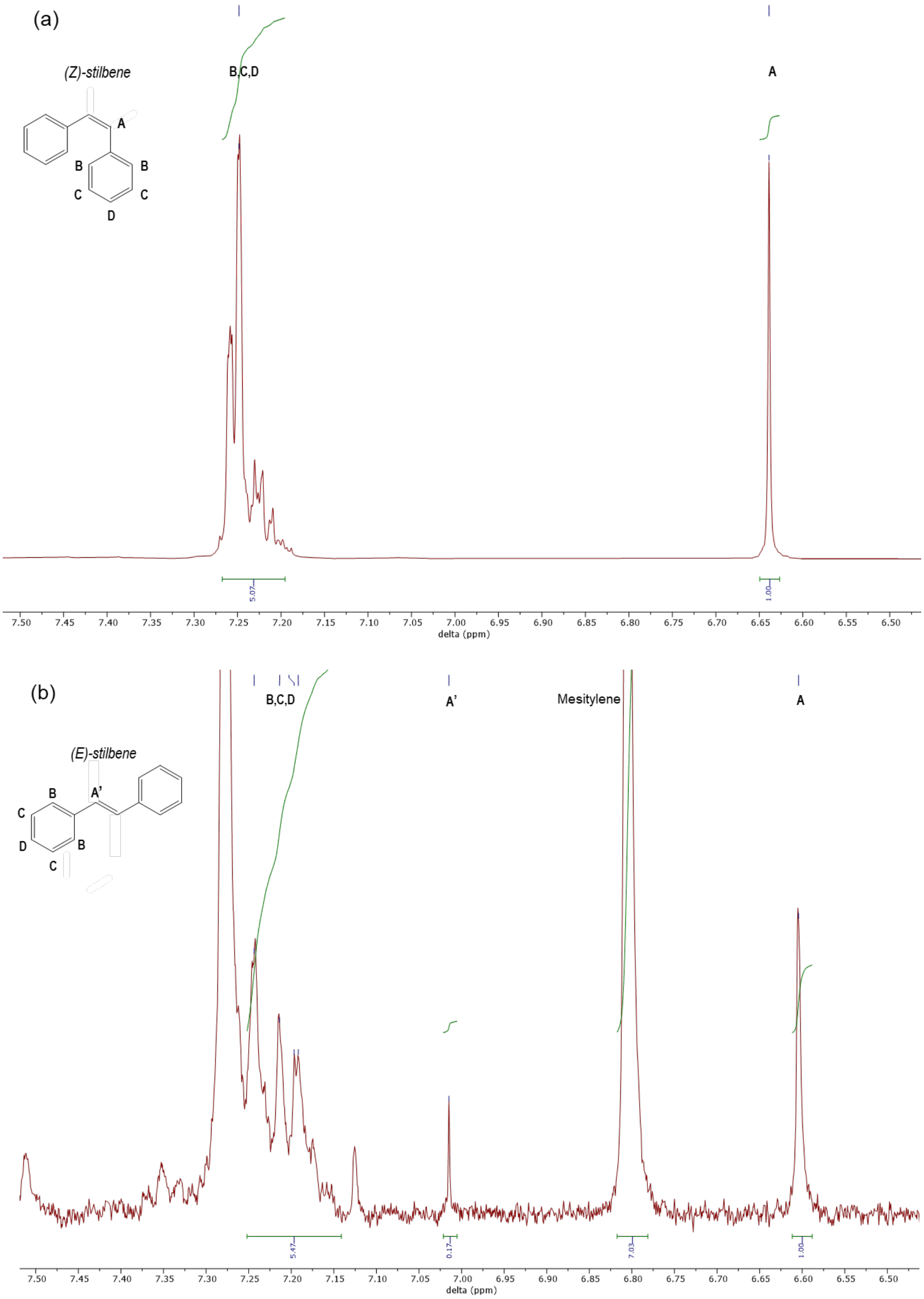

Fig. S27 ^{1}H NMR spectra of (a) (*Z*)-stilbene ((*Z*)-**4**H_2) standard in DMF and (b) electrolytic mixture after 50 min of electrolysis of diphenylacetylene (**4**).

(*Z*)-stilbene ((*Z*)-**4**H_2): δ_H = 7.27 – 7.20 (m, 10H; $\mathbf{H_{B,C,D}}$), 6.64 (s, 2H; $\mathbf{H_A}$).

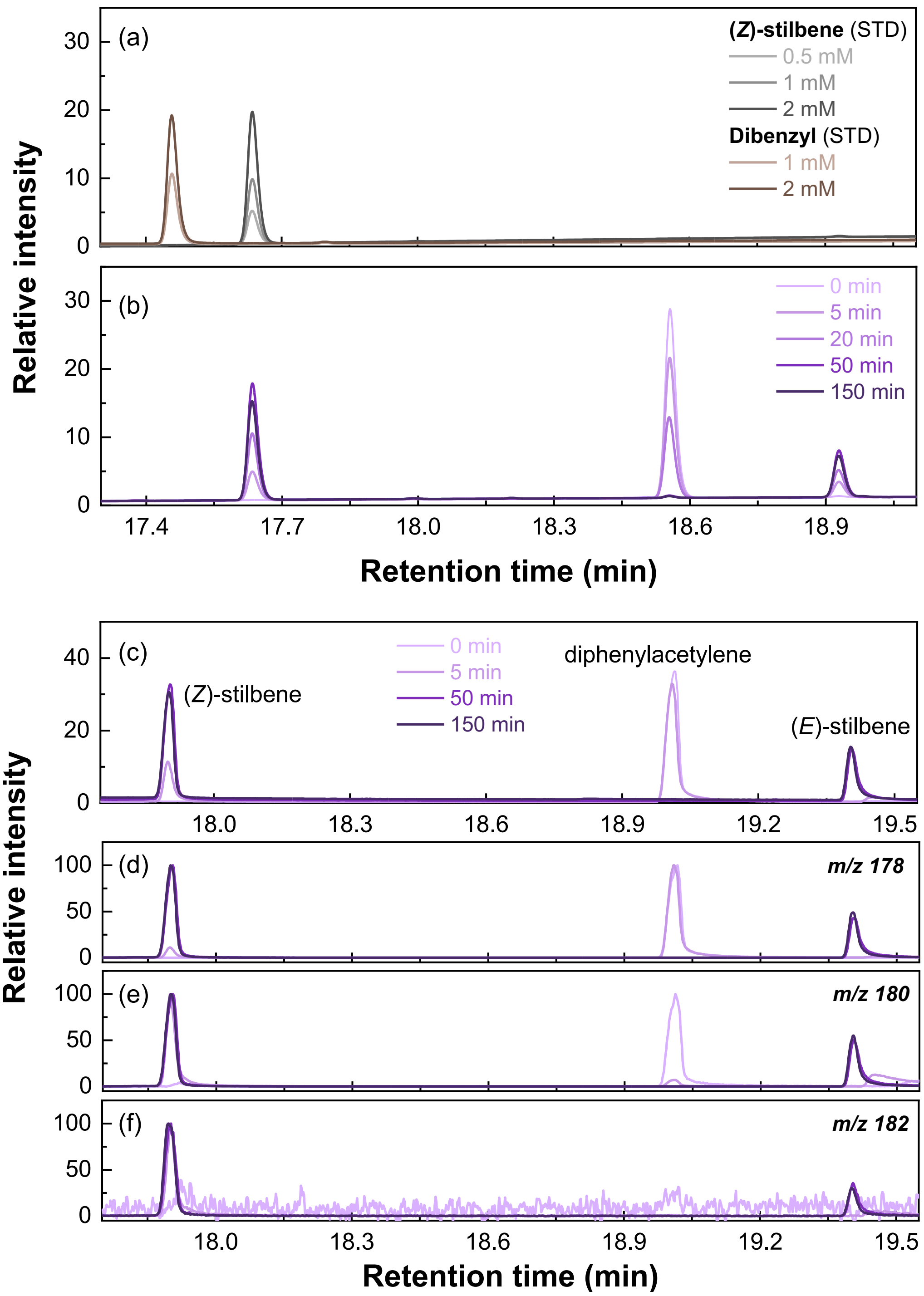

Fig. S28 (a) GC-FID chromatograms of dibenzyl (**4**H_4) and (*Z*)-stilbene ((*Z*)-**4**H_2) reference products in DMF. GC chromatograms of the electrolytic mixture during electrolysis of diphenylacetylene (**4**) to stilbene (**4**H_2): (b) GC-FID and (c-f) GC-MS.

5: 1-octyne

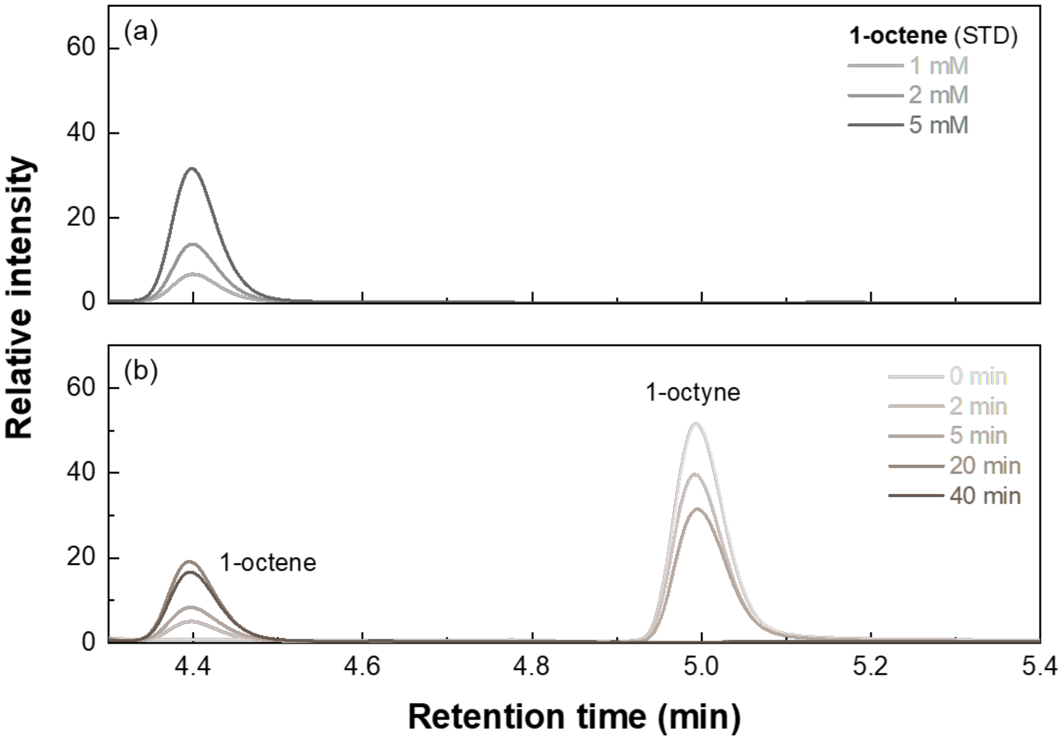

Fig. S29 GC-FID chromatograms: (a) 1-octene (**5**H_2) reference product in DMF and (b) the electrolytic mixture at various times during electrolysis of 1-octyne (**5**).

6: phenylacetylene

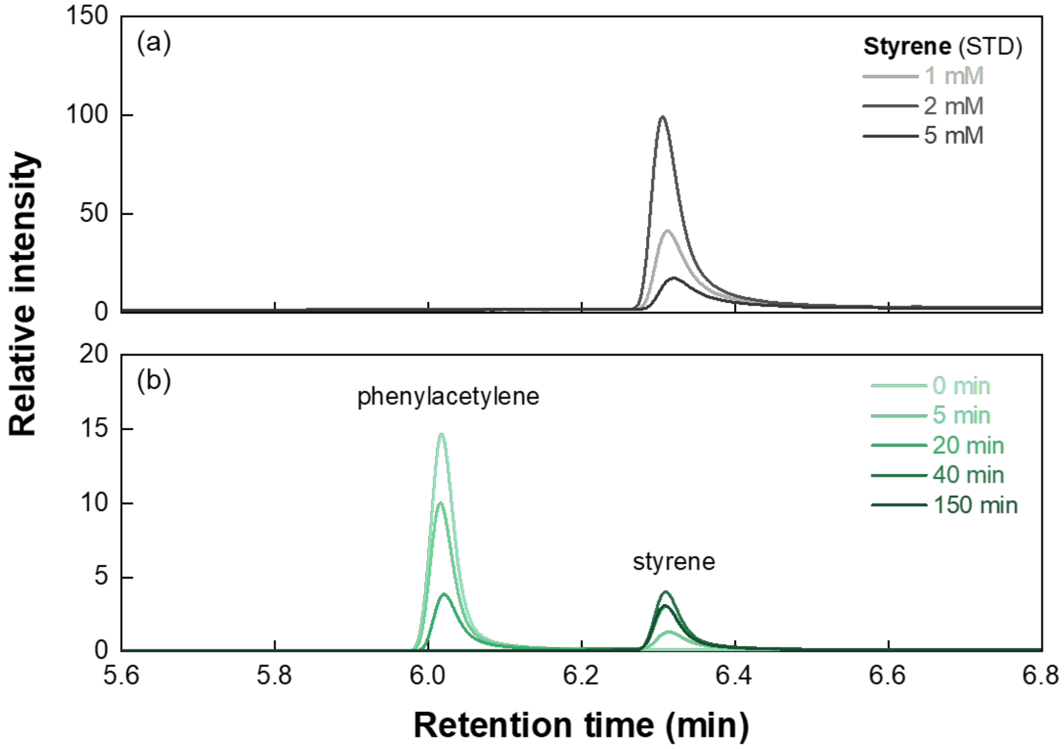

Fig. S30 GC-FID chromatograms: (a) styrene (**6**H_2) reference product in DMF and (b) the electrolytic mixture at various times during electrolysis of phenylacetylene (**6**).

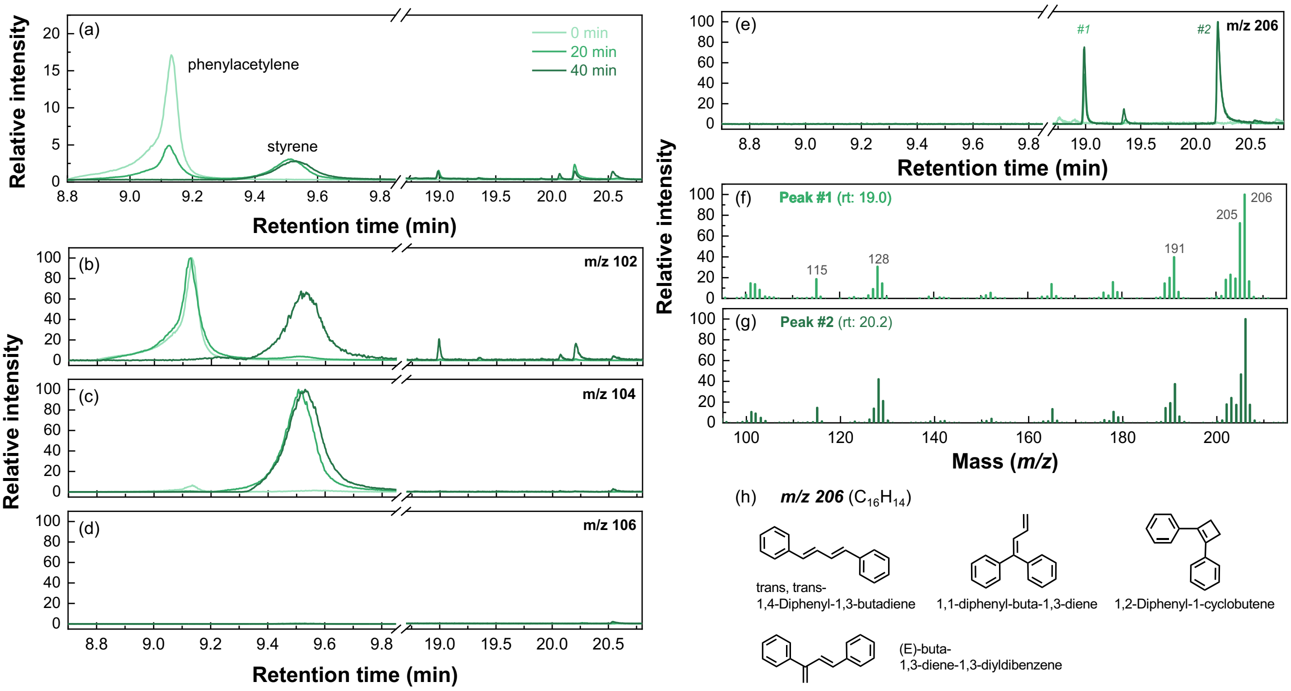

Fig. S31 GC-MS chromatograms: (a) electrolytic mixture during electrolysis of phenylacetylene (**6**) and (b)-(d) specific traces for m/z =102 (b), 104 (c), 106 (d) and 206 (e). (f, g) Mass spectra for peaks at m/z = 206 with retention time at 19.0 min (f) and at 20.2 min (g), and (h) possible fragments of m/z = 206.

7: 1,4-diphenylbutadiyne

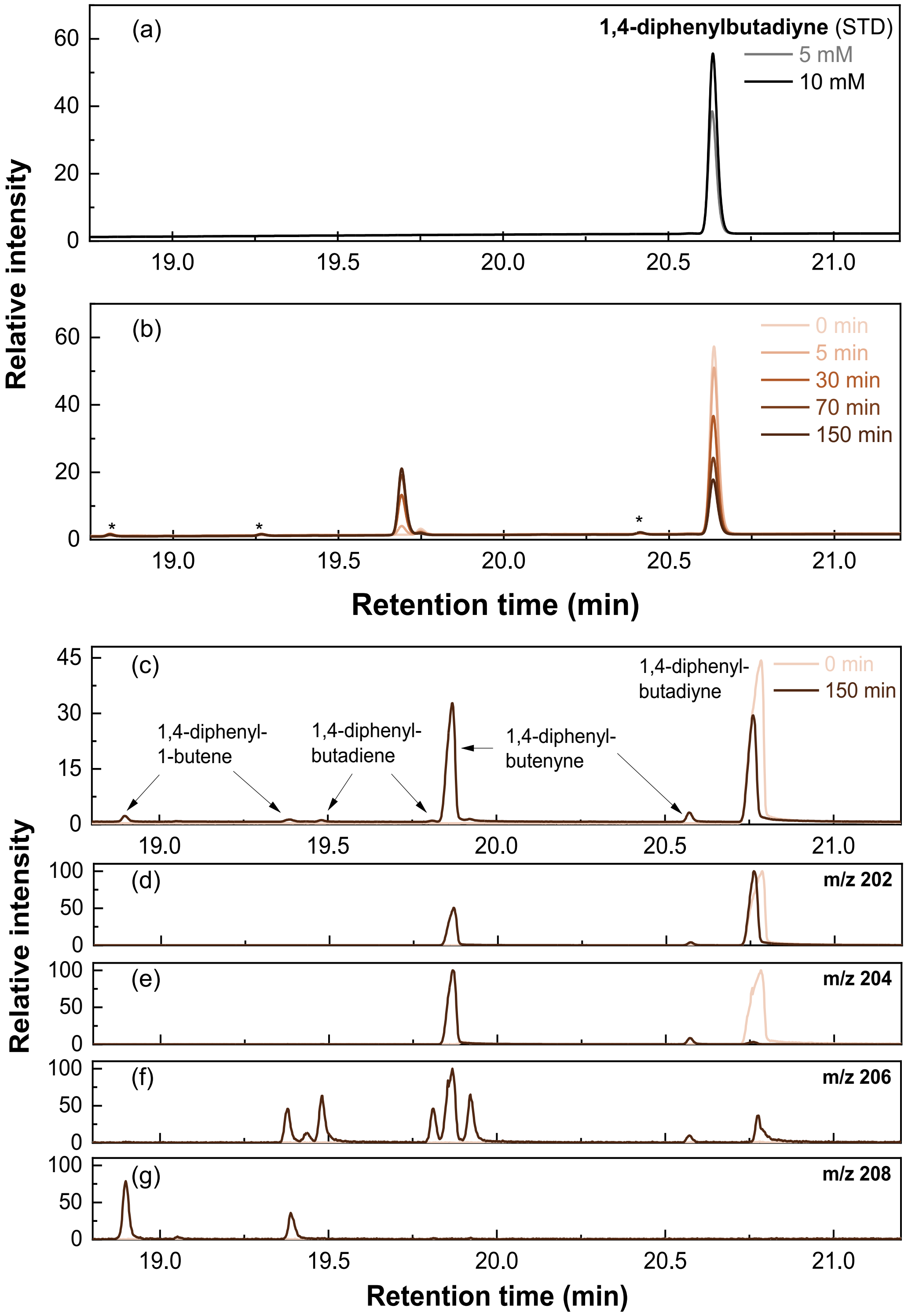

Fig. S32 (a) GC-FID chromatogram of 1,4-diphenylbutadiyne (**7**) reference product in DMF. GC chromatograms of the electrolytic mixture during electrolysis of 1,4-diphenylbutadiyne (**7**): (b) GC-FID and (c-g) GC-MS.

8: 1-chloro-4-(phenylethynyl)benzene (4-chloro-diphenylacetylene)

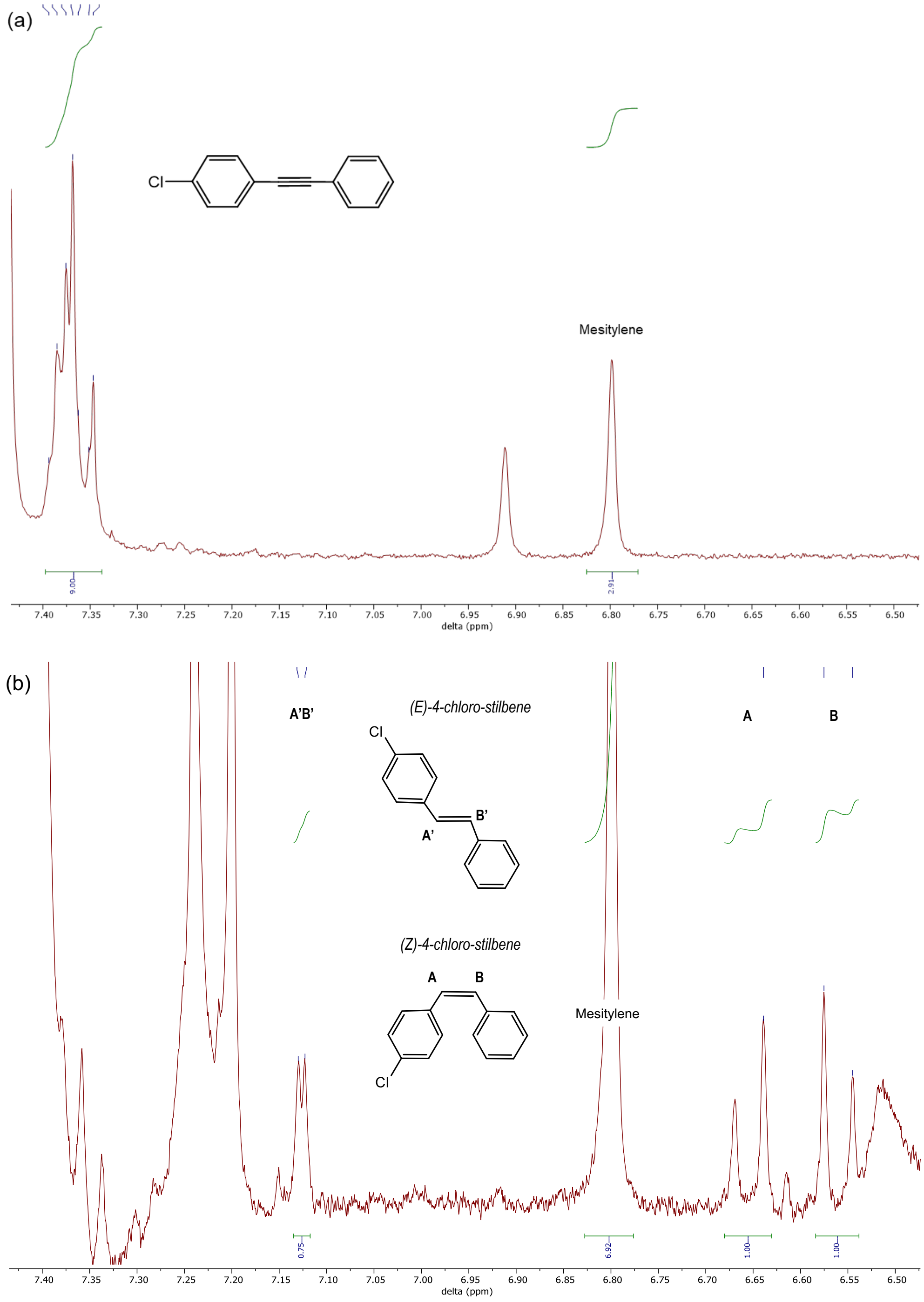

Fig. S33 ^{1}H NMR spectra of crude samples of the electrolytic mixture of 4-chloro-diphenylacetylene (**8**): (a) before electrolysis and (b) after 100 min.

4-chloro-stilbene (**8**H_2) diastereoisomers attributed from literature data.[11]

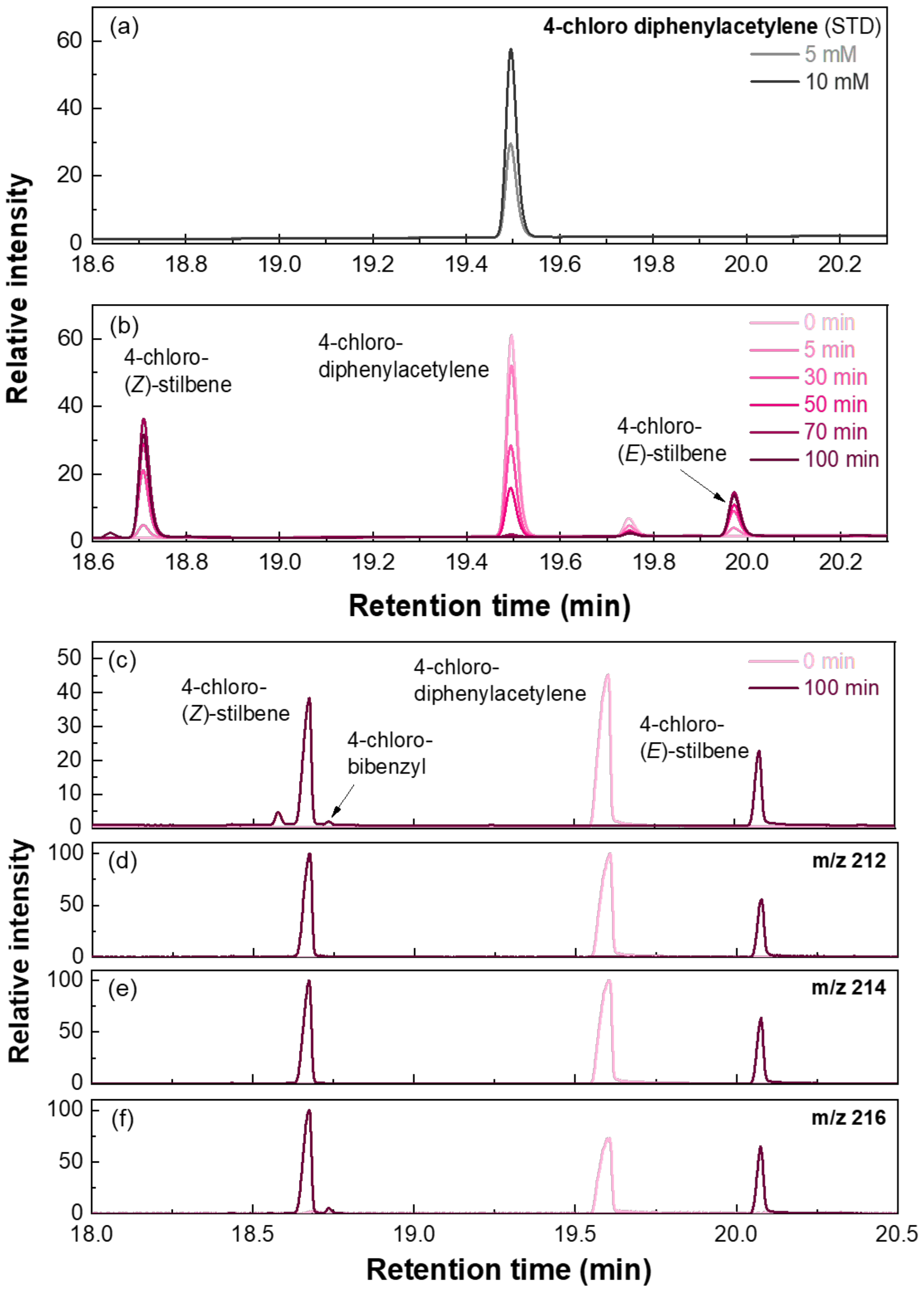

Fig. S34 (a) GC-FID chromatogram of 4-chloro-diphenylacetylene (**8**) reference product in DMF. GC chromatograms of the electrolytic mixture during electrolysis of 4-chloro-diphenylacetylene (**8**) to 4-chloro-stilbene (**8**H_2): (b) GC-FID and (c-f) GC-MS.

9: 1-bromo-4-(phenylethynyl)benzene (4-bromo-diphenylacetylene)

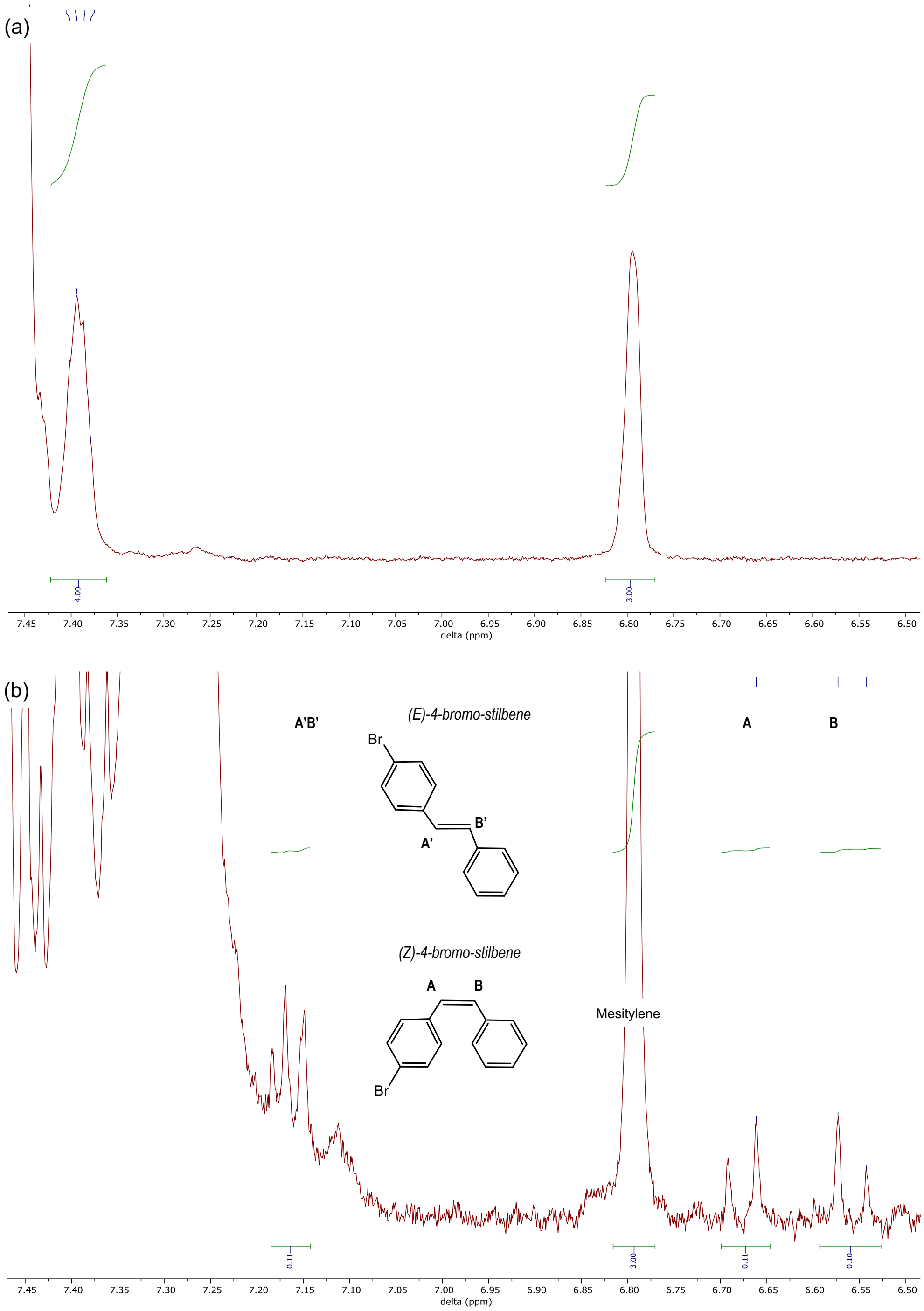

Fig. S35 ^{1}H NMR spectra of crude samples of the electrolytic mixture of 4-bromo-diphenylacetylene (**9**): (a) before electrolysis and (b) after 100 min.

Attribution of 4-bromo-stilbene (**9**H_2) diastereoisomers proposed based on literature data for (*E*)-**9**H_2.[12]

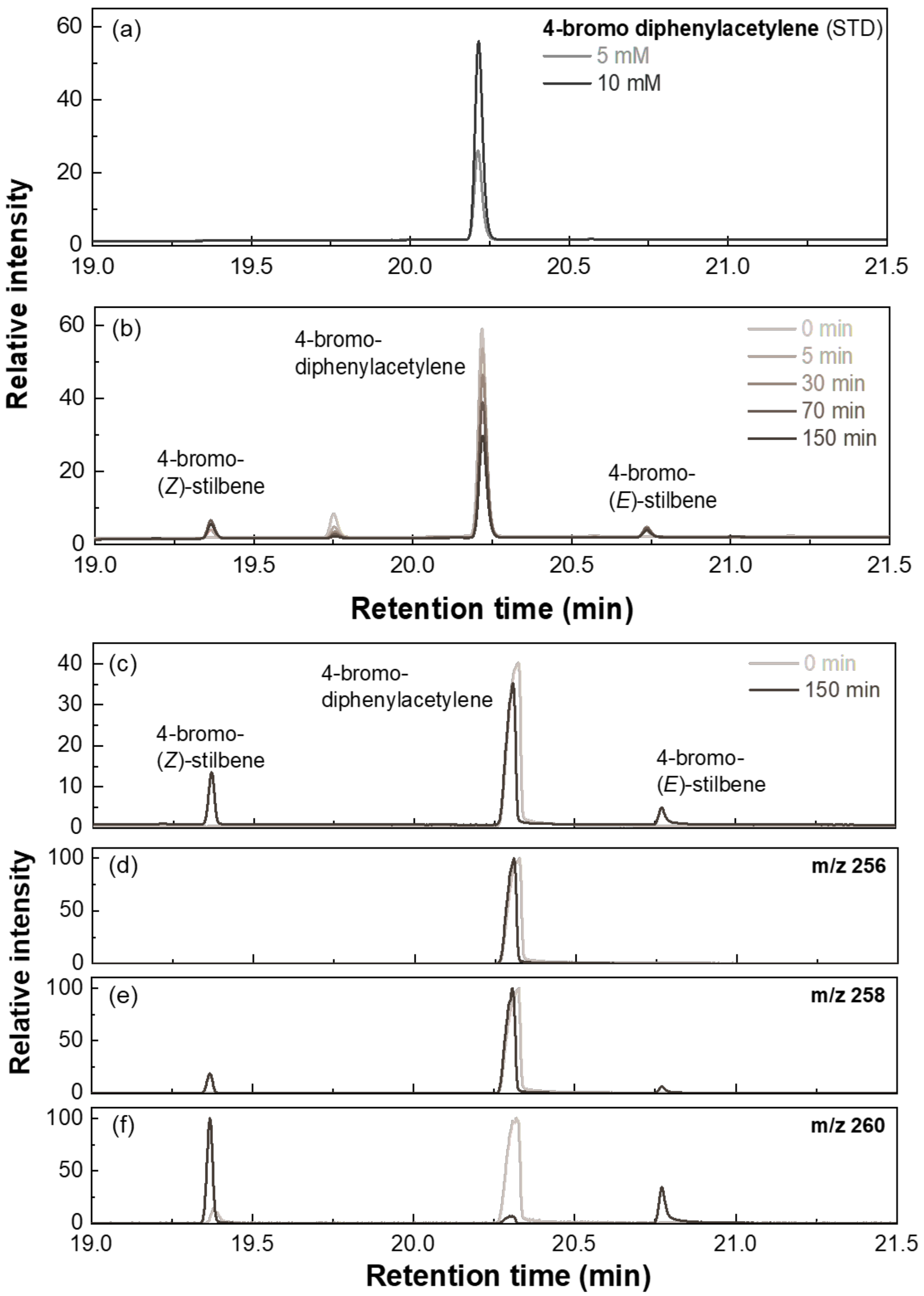

Fig. S36 (a) GC-FID chromatogram of 4-bromo-diphenylacetylene (**9**) reference product in DMF. GC chromatograms of the electrolytic mixture during electrolysis of 4-bromo-diphenylacetylene (**9**) to 4-bromo-stilbene (**9**H_2): (b) GC-FID and (c-f) GC-MS.

10: 3-phenyl-2-propyn-1-ol

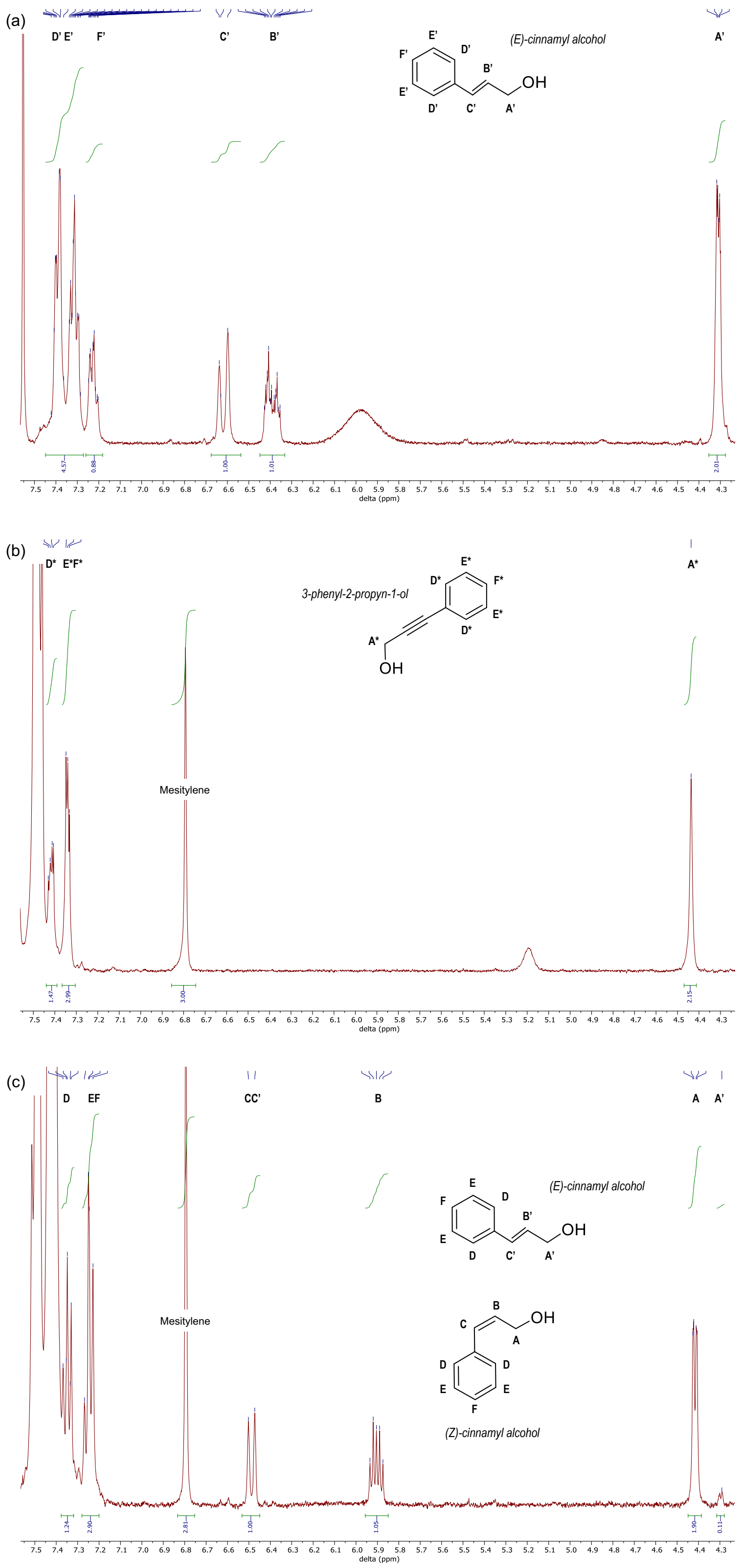

Fig. S37 ^{1}H NMR spectra of (a) (E)-cinnamyl alcohol ((*E*)-**10**H_2) standard in DMF and of crude samples of the electrolytic mixture of 3-phenyl-2-propyn-1-ol (**10**): (b) before electrolysis and (c) after 15 min. (*Z*)-cinnamyl alcohol ((*Z*)-**10**H_2) attributed from literature data.[13]

Table S5. Electrocatalytic hydrogenation of alkynes **S** with **Ni** at room temperature ([**Ni**] = 1 mM; [**S**] = 10 mM; [**BA**] = 100 mM; DMF; 0.1 M *n*Bu_4NPF_6; E_{app} = –1.93 V_{Fc}; 2.5 h). [a]*Time to full conversion of* ***S***. [b]*Not fully converted at 2.5 h.* [c]*NMR yields.*

S	Time (min)	Yield **S**H_2 (%)	TON	*Z*/*E*	FE (%)
**1**	40[a]	68.4 ± 5.7	6.8 ± 0.6	95:5	53.5 ± 3.0
	150	65.5 ± 4.8	6.5 ± 0.5	95:5	14.5 ± 0.6
**2**	20[a]	84.9 ± 3.1	8.5 ± 0.3	92:8	59.3 ± 10.6
	150	81.3 ± 2.5	8.1 ± 0.3	92:8	11.3 ± 0.2
**3**	30[a]	93.3 ± 0.9	9.3 ± 0.1	83:17	51.8 ± 0.6
	150	92.4 ± 0.8	9.2 ± 0.1	83:17	24.6 ± 1.2
**4**	50[a]	83.4	8.3	61:39	23.5
	150	81.2	8.2	61:39	12.2
**5**	20[a]	38.9	3.9	-	29.9
	150	36.2	3.6		4.3
**6**	40[a]	38.4 ± 3.6	3.8 ± 0.4	-	24.4 ± 0.7
	150	37.5 ± 4.5	3.7 ± 0.4	-	9.1 ± 2.5
**7**	150	45.0[b]	4.5	-	20.5
**8**	100[a]	85.5	8.5	70:30	46.1
	150	73.7	7.4	69:31	30.6
**9**	150	10.1[b]	1.0	63:37	4.8
**10**	15[a]	93.5[c]	9.3	95:5	97.9

3.2.5 Isolated yield for stilbene

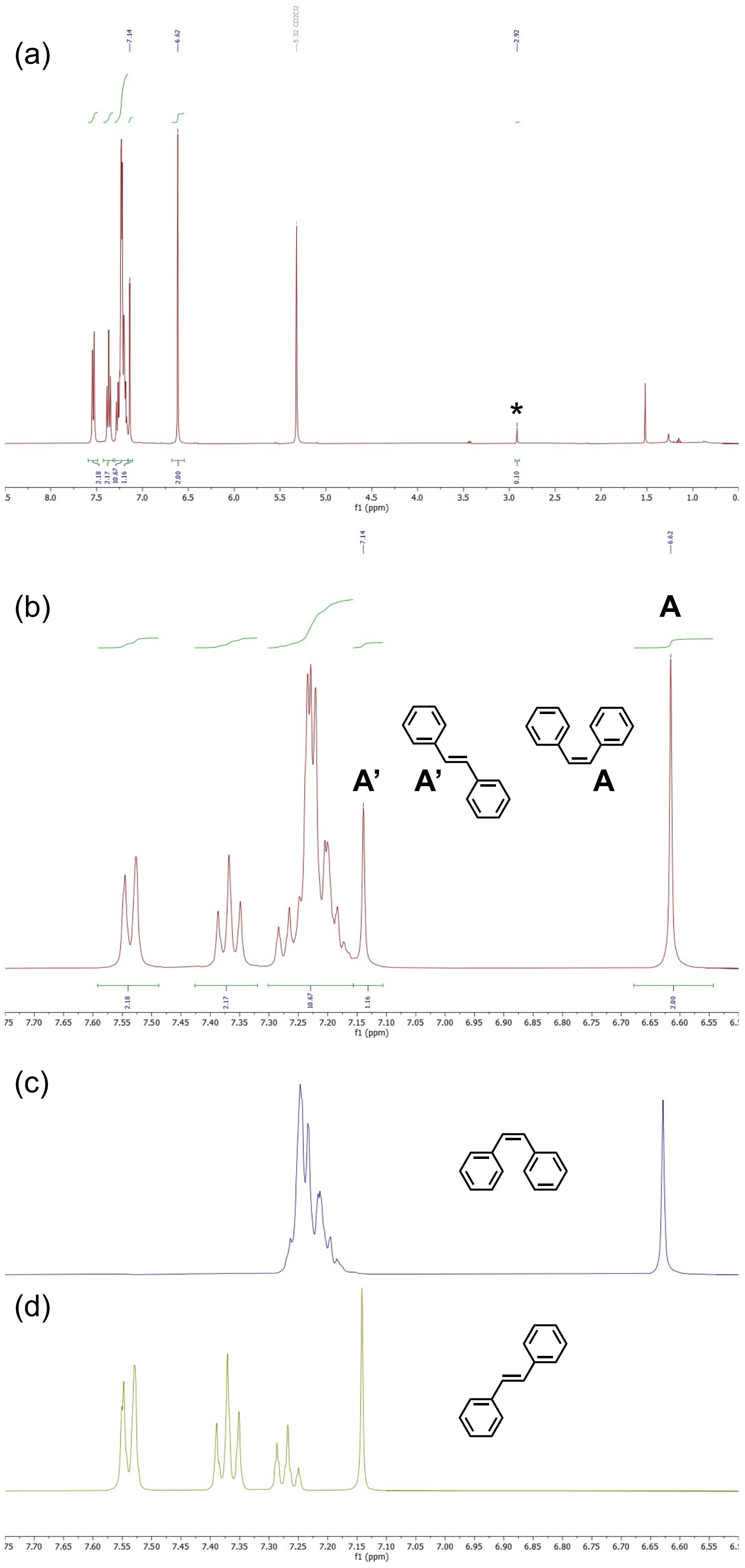

Fig. S38 ^{1}H NMR spectra (CD_2Cl_2) of (a,b) the crop isolated from the electrolysis of diphenylacetylene (**4**) and standard of (c) (*Z*)-stilbene ((*Z*)-**4**H_2) and (d) (*E*)-stilbene ((*E*)-**4**H_2).* corresponds to the overhydrogenated dibenzyl (**4**H_4) impurity. Isolated **4**H_2: 48.2 mg; 53% yield; *Z*/*E* 63:27; 98.5% purity (1.5% **4**H_4).

3.3 SEC analysis

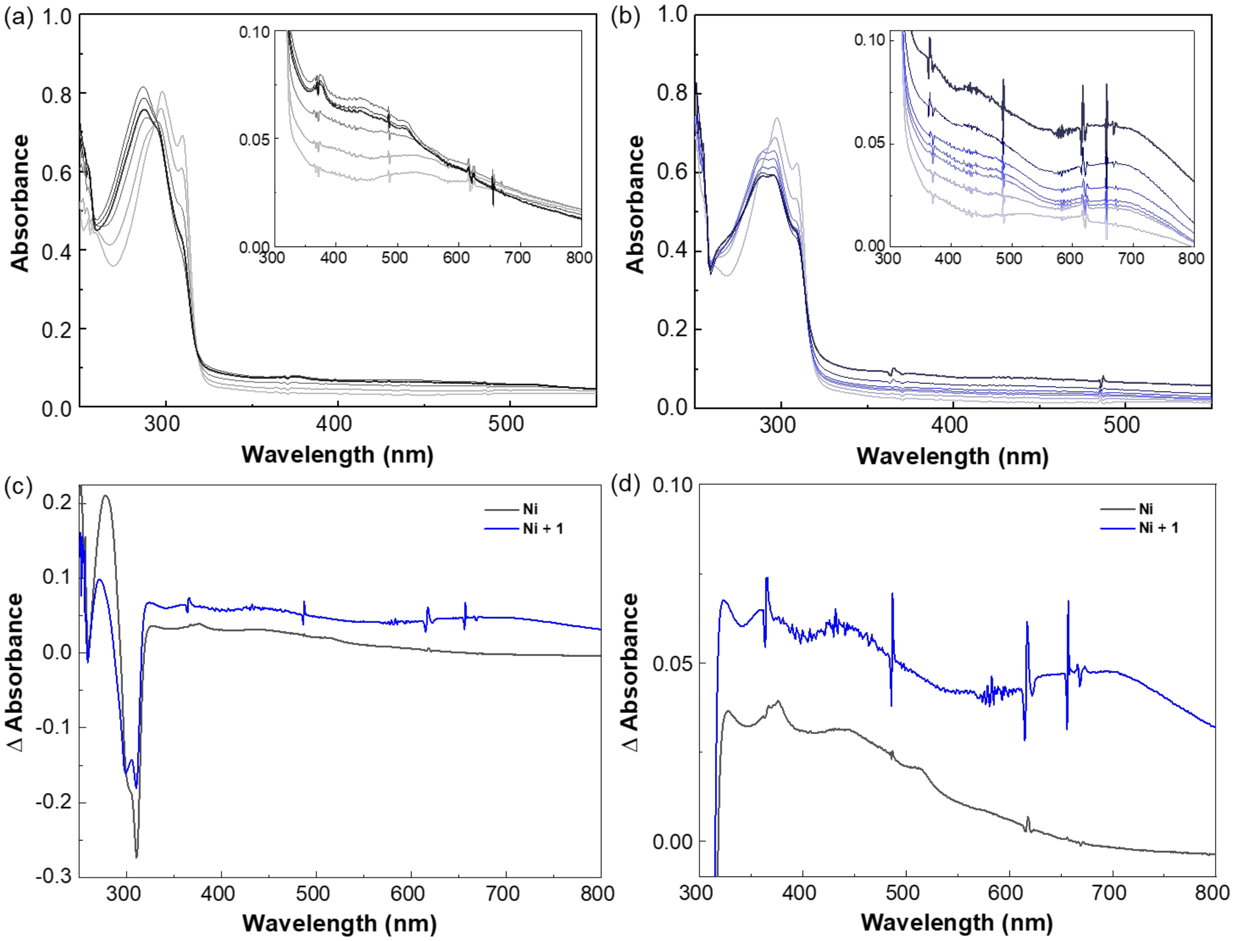

Fig. S39. UV-SEC measurements: (a-b) time-dependent UV absorbance of (a) **Ni** (black) and (b) **Ni** with **1** (blue), where lines become darker with increasing time, and (c-d) differential UV absorbance spectra obtained by subtraction of the UV spectrum recorded without applied potential from the one at 10 min under applied potential (E_{app}). [**Ni**] = 0.25 mM; [**1**] = 2.5 mM; DMF; 0.1 M nBu$_4$NPF$_6$; E_{app} = –1.93 V_{Fc}; 10 min.

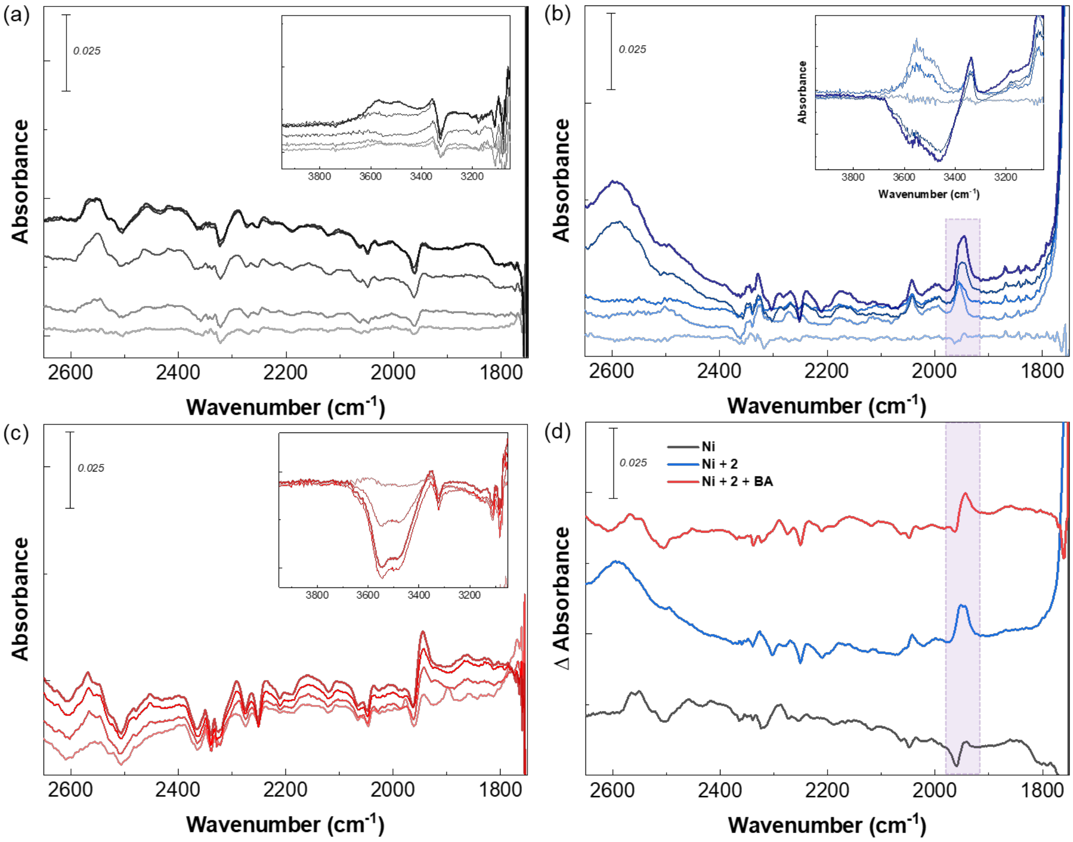

Fig. S40. IR-SEC data: (a-c) time-dependent IR absorbance for (a) **Ni** (black), (b) **Ni** with **2** (blue), and (c) **Ni** with **2** and **BA** (red), where spectra become darker with increasing time, and (d) differential IR absorbance spectra obtained by subtraction of the IR spectrum recorded without applied potential from the one at 5 min under applied potential (E_{app}). [**Ni**] = 10 mM; [**2**] = 100 mM; [**BA**] = 10 mM; DMF; 0.1 M *n*Bu_4NPF_6; E_{app} = –1.9 V vs. Ag; 5 min.

The IR-SEC experiment conducted with **Ni** and 1-phenyl-1-propyne **2** (used to avoid overlay with solvent signature) produces a band at 1924 cm^{-1} (Fig. S40b,d), which is not observed in the absence of **2**. This value is intermediate between the ones encountered for unsaturated C-C bond stretching in disubstituted alkynes (2200-2250 cm^{-1} region) and in 1,2-disubstituted alkenes (1600-1650 cm^{-1} region).[14-16] We thus attribute the band at 1924 cm^{-1} to the C=C stretching of the nickelacyclopropene ring in [Ni(bpy)**2**], as the hybridization is expected to deviate from triple bond character to substantial double bond character. This value is also close to the C=C stretching in the related tetramethylcyclopropene (1877 cm^{-1})[17] and ca 150 cm^{-1} blue-shifted compared to that proposed for the C=C bond stretching in the congener nickelacyclopropene ring of [Ni(bpy)**4**] (1770 cm^{-1}).[18]

3.4 Additional mechanistic discussion

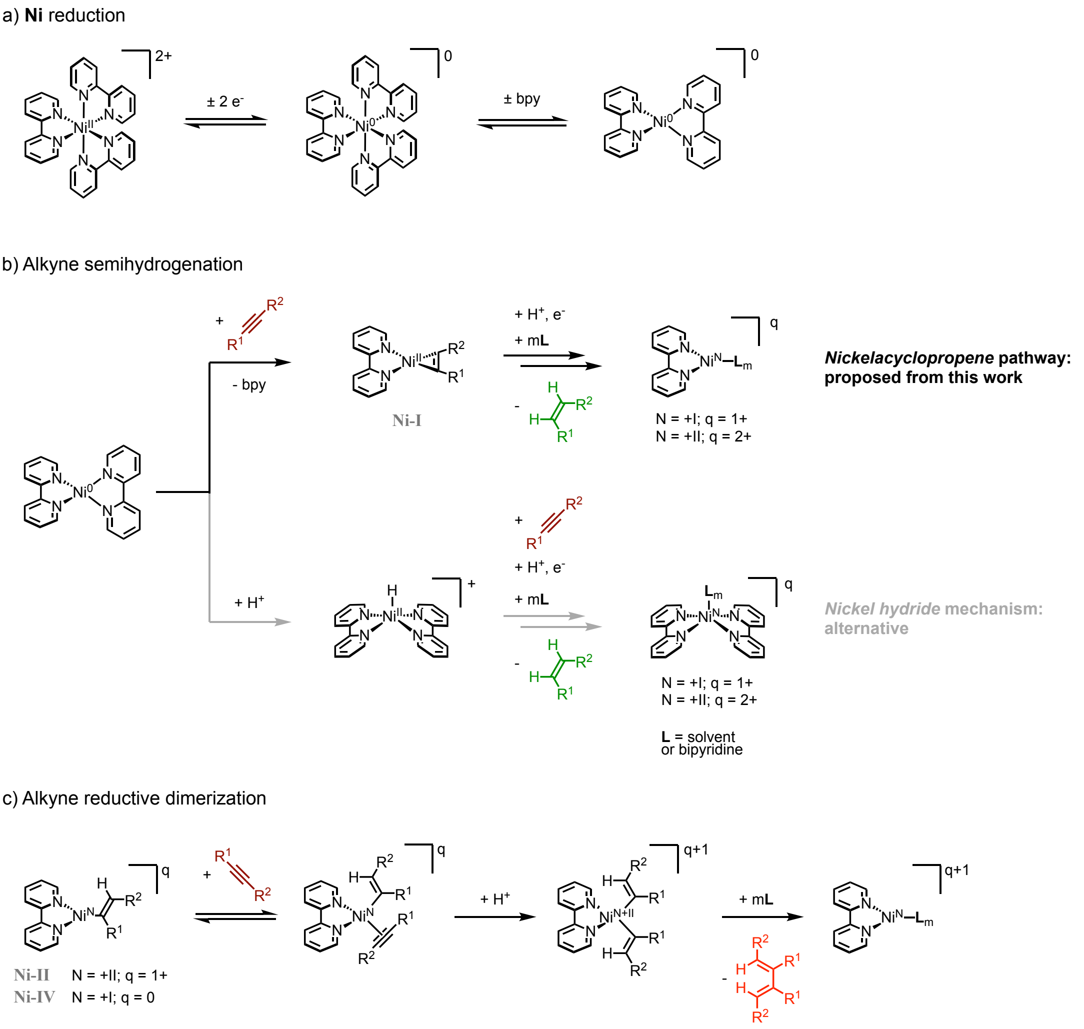

Fig. S41 Mechanistic schemes: a) **Ni** reduction, b) alkyne semihydrogenation via a nickelacyclopropene pathway as proposed in this work and via a nickel hydride pathway as possible alternative and c) possible route for alkyne reductive dimerization.

Using the stronger trifluoroacetic acid (**TFA**, pKa = 4.0 estimated in DMF from data in MeCN[5] transposed in DMF[19]), we found that adding the acid only (**Ni/TFA** 1:1-50; Fig. S10a) is sufficient to trigger electrocatalytic waves at potentials $E_{p,c}$ = –1.62 V_{Fc} and –1.87 V_{Fc}. This electrocatalytic behavior in the presence of only the proton source points to hydrogen evolution catalyzed by **Ni** and thus to the involvement of Ni-hydride species of the form $[Ni(bpy)_nH]^q$. The second electrocatalytic wave at $E_{p,c}$ = –1.87 V_{Fc} is magnified by introduction of **2** (**Ni/2/TFA** 1:1-100:50; Fig. S10b). This increase suggests that the reduced Ni-hydride is also competent to transfer the hydride to the triple C-C bond. The electrocatalytic activity towards alkyne is further assessed by electrolysis of **1** with TFA (**Ni/1/TFA** 1:10:100), which produces the alkene **1**H_2 in 38% yield and 3.8 TONs (Table S2). This result confirms that **TFA** is a suitable acid for alkyne semihydrogenation

electrocatalyzed by **Ni.** Altogether, a mechanism for alkyne semihydrogenation shuttling *via* a Ni hydride complex is thus more plausible with strong acids such as **TFA** (Fig. S41b).

An electrocatalytic wave developing from –1.80 V_{Fc} being also observed at high excess of **BA** vs **2** (**Ni/2/BA** 1:5:1-50; Fig. S8a) suggest that a Ni hydride mechanism may also operate with that acid, although at potentials more cathodic than the mechanism involving a nickelacyclopropene intermediate.

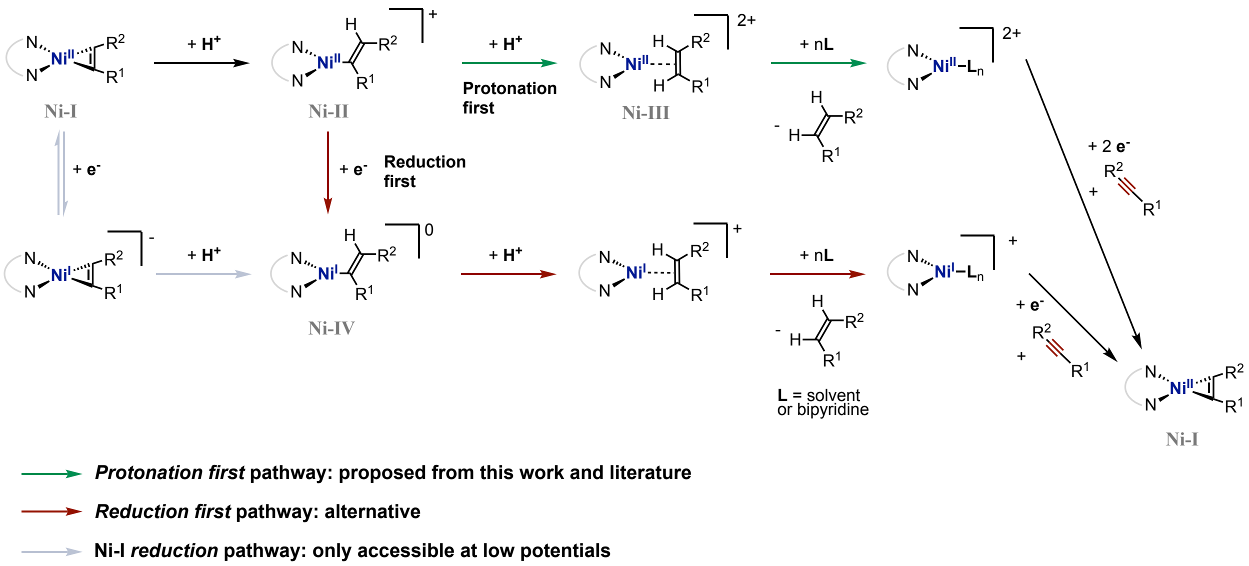

Fig. S42 Pathways for electrocatalytic alkyne semihydrogenation in the nickelacyclopropene mechanism.

Finally, we observed that phenylpropyne **2** added in increasing excess vs **Ni** and **BA** (**Ni/2/BA** 1:1-100:50) triggers a catalytic wave developing at the potential $E_{p,c}$ = –2.25 V_{Fc} (Fig. S9) close to the [Ni(bpy)**2**]$^{0/–}$ couple ($E_{1/2}$ = –2.19 V_{Fc}; Fig. S7) while electrocatalytic waves at more positive potentials are partially turned off. This observation shows that the pathway involving the reduction of nickelacyclopropene intermediate [Ni(bpy)**S**] (**Ni-I**) (Fig. S42) is actually accessible at high alkyne excess, although at very cathodic potential.

4. References

1. Hathaway, B. J.; Holah, D. G.; Underhill, A. E., 468. The preparation and properties of some bivalent transition-metal tetrafluoroborate–methyl cyanide complexes. *J. Chem. Soc.* **1962,** (0), 2444-2448.
2. Krejčik, M.; Daněk, M.; Hartl, F., Simple construction of an infrared optically transparent thin-layer electrochemical cell: Applications to the redox reactions of ferrocene, Mn2(CO)10 and Mn(CO)3(3,5-di-t-butyl-catecholate)−. *J. Electroanal. Chem.* **1991,** *317* (1), 179-187.
3. Lee, K. J.; McCarthy, B. D.; Dempsey, J. L., On decomposition, degradation, and voltammetric deviation: the electrochemist's field guide to identifying precatalyst transformation. *Chem. Soc. Rev.* **2019,** *48* (11), 2927-2945.
4. Kaeffer, N.; Morozan, A.; Fize, J.; Martinez, E.; Guetaz, L.; Artero, V., The Dark Side of Molecular Catalysis: Diimine–Dioxime Cobalt Complexes Are Not the Actual Hydrogen Evolution Electrocatalyst in Acidic Aqueous Solutions. *ACS Cat.* **2016,** *6* (6), 3727-3737.
5. Fourmond, V.; Jacques, P. A.; Fontecave, M.; Artero, V., H2 evolution and molecular electrocatalysts: determination of overpotentials and effect of homoconjugation. *Inorg. Chem.* **2010,** *49* (22), 10338-47.
6. Connelly, S. J.; Wiedner, E. S.; Appel, A. M., Predicting the reactivity of hydride donors in water: thermodynamic constants for hydrogen. *Dalton Trans.* **2015,** *44* (13), 5933-5938.
7. Joback, K. G.; Reid, R. C., ESTIMATION OF PURE-COMPONENT PROPERTIES FROM GROUP-CONTRIBUTIONS. *Chem. Eng. Commun.* **1987,** *57* (1-6), 233-243.
8. Brunner, E., Solubility of hydrogen in 10 organic solvents at 298.15, 323.15, and 373.15 K. *J. Chem. Eng. Data* **1985,** *30* (3), 269-273.
9. Abraham, M. H.; Acree Jr, W. E.; Cometto-Muñiz, J. E., Partition of compounds from water and from air into amides. *New J. Chem.* **2009,** *33* (10), 2034-2043.
10. Savéant, J.-M., Single Electron Transfer at an Electrode. In *Elements of Molecular and Biomolecular Electrochemistry*, John Wiley & Sons: 2006; pp 1-77.
11. Aggarwal, V. K.; Fulton, J. R.; Sheldon, C. G.; de Vicente, J., Generation of phosphoranes derived from phosphites. A new class of phosphorus ylides leading to high E selectivity with semi-stabilizing groups in Wittig olefinations. *J. Am. Chem. Soc.* **2003,** *125* (20), 6034-5.
12. Zhang, N.; Quan, Z. J.; Zhang, Z.; Da, Y. X.; Wang, X. C., Synthesis of stilbene derivatives via visible-light-induced cross-coupling of aryl diazonium salts with nitroalkenes using -NO2 as a leaving group. *Chem. Commun.* **2016,** *52* (99), 14234-14237.

13. Vyas, D. J.; Oestreich, M., Expedient access to branched allylic silanes by copper-catalysed allylic substitution of linear allylic halides. *Chem. Commun.* **2010,** *46* (4), 568-70.

14. Coates, J., Interpretation of Infrared Spectra, A Practical Approach. In *Encyclopedia of Analytical Chemistry*, 2006.

15. Lin-Vien, D.; Colthup, N. B.; Fateley, W. G.; Grasselli, J. G., CHAPTER 6 - Alkenes. In *The Handbook of Infrared and Raman Characteristic Frequencies of Organic Molecules*, Lin-Vien, D.; Colthup, N. B.; Fateley, W. G.; Grasselli, J. G., Eds. Academic Press: San Diego, 1991; pp 73-94.

16. Lin-Vien, D.; Colthup, N. B.; Fateley, W. G.; Grasselli, J. G., CHAPTER 7 - Acetylenes. In *The Handbook of Infrared and Raman Characteristic Frequencies of Organic Molecules*, Lin-Vien, D.; Colthup, N. B.; Fateley, W. G.; Grasselli, J. G., Eds. Academic Press: San Diego, 1991; pp 95-104.

17. Schrader, B., Vibrational spectroscopy of different classes and states of compounds. In *Infrared and Raman Spectroscopy: Methods and Applications*, 1995.

18. Eisch, J. J.; Ma, X.; Han, K. I.; Gitua, J. N.; Kruger, C., Mechanistic comparison of the nickel(0)-catalyzed homo-oligomerization and co-oligomerization of alkynes and nitriles. *Eur. J. Inorg. Chem.* **2001,** *2001* (1), 77-88.

19. Kütt, A.; Tshepelevitsh, S.; Saame, J.; Lõkov, M.; Kaljurand, I.; Selberg, S.; Leito, I., Strengths of Acids in Acetonitrile. *Eur. J. Org. Chem.* **2021,** *2021* (9), 1407-1419.
